# Picornavirus infection enhances aspartate by the SLC38A8 transporter to promote viral replication

**DOI:** 10.1371/journal.ppat.1011126

**Published:** 2023-02-03

**Authors:** Huisheng Liu, Zixiang Zhu, Qiao Xue, Fan Yang, Weijun Cao, Zhaoning Xue, Xiangtao Liu, Haixue Zheng

**Affiliations:** State Key Laboratory of Veterinary Etiological Biology; College of Veterinary Medicine, Lanzhou University, WOAH/National reference laboratory for foot-and-mouth disease; Lanzhou Veterinary Research Institute, Chinese Academy of Agricultural Sciences, Lanzhou, China; University of Maryland, UNITED STATES

## Abstract

Foot-and-mouth disease, a class of animal diseases, is caused by foot-and-mouth disease virus (FMDV). The metabolic changes during FMDV infection remain unclear. Here, PK-15 cells, serum, and tonsils infected with FMDV were analyzed by metabolomics. A total of 284 metabolites in cells were significantly changed after FMDV infection, and most of them belong to amino acids and nucleotides. Further studies showed that FMDV infection significantly enhanced aspartate *in vitro and in vivo*. The amino acid transporter solute carrier family 38 member 8 (SLC38A8) was responsible for FMDV-upregulated aspartate. Enterovirus 71 (EV71) and Seneca Valley virus (SVV) infection also enhanced aspartate by SLC38A8. Aspartate aminotransferase activity was also elevated in FMDV-, EV71-, and SVV-infected cells, which may lead to reversible transition between the TCA cycle and amino acids synthesis. Aspartate and SLC38A8 were essential for FMDV, EV71, and SVV replication in cells. In addition, aspartate and SLC38A8 also promoted FMDV and EV71 replication in mice. Detailed analysis indicated that FMDV infection promoted the transfer of mTOR to lysosome to enhance interaction between mTOR and Rheb, and activated PI3K/AKT/TSC2/Rheb/mTOR/p70S6K1 pathway to promote viral replication. The mTORC1 signaling pathway was responsible for FMDV-induced SLC38A8 protein expression. For the first time, our data identified metabolic changes during FMDV infection. These data identified a novel mechanism used by FMDV to upregulate aspartate to promote viral replication and will provide new perspectives for developing new preventive strategies.

## Author summary

Viruses can utilize cells to synthesize many metabolites required for self-replication. However, the impact of foot-and-mouth disease virus (FMDV) infection on host cellular metabolism remains unknown. Here, we analyzed the metabolomic profiles in FMDV-infected PK-15 cells and pigs for the first time. FMDV infection significantly enhanced aspartate, and the solute carrier family 38 member 8 transporter was responsible for this effect. FMDV infection activated the mTOR/p70S6K1 signaling axis to promote viral replication. This study revealed how FMDV uses amino acids to promote viral replication, which is important for guiding the design of new preventive measures against FMDV targeting the altered metabolic pathways.

## Introduction

Foot-and-mouth disease virus (FMDV), an Aphthovirus within the viral family *Picornaviridae*, is a single-stranded positive-sense RNA virus that causes foot-and-mouth disease (FMD) in domestic and wild cloven-hoofed animals worldwide, including swine, goats, sheep, cattle, deer, and camelids (1). There are seven known serotypes of FMDV (O, A, Asia1, C, SAT1, SAT2, and SAT3) and multiple subtypes [1]. FMDV contains a genome of approximately 8.5 Kb, which encodes a single polyprotein that is subsequently cleaved into four structural and eight non-structural proteins [1].

The virus-host interaction and its regulatory mechanism determine the pathogenicity of the virus [2]. The host’s innate immune response is the first line of defense against viral infection. FMDV always negatively regulates the host innate immune response to promote self-replication. For example, FMDV VP1 targets the mitochondrial antiviral signaling protein (MAVS) to inhibit type-I interferon signaling [3]. FMDV 2B or 2C reduce adaptor molecules’ protein expression, including retinoic-acid inducible gene I (RIG-I), laboratory of genetics and physiology 2 (LGP2), nucleotide-binding oligomerization domain 2 (NOD2), and receptor-interacting protein 2 (RIP2) in the innate immune signaling pathway to facilitate viral replication [4,5]. FMDV 3B protein interacts with RIG-I to block RIG-I-mediated immune signaling and antiviral response [6]. FMDV L^pro^ and 3C^pro^ inhibits host innate immune response to promote viral replication in different manners [7–9]. In addition to innate immunity, the interaction between virus and cellular metabolism also plays an important role in viral pathogenicity.

Cellular metabolism mainly involves glycolysis, the tricarboxylic acid cycle (TCA cycle), amino acid metabolism, lipid metabolism, and nucleotide metabolism. Metabolomics, an important means to identify metabolic changes in host cells, is a new method used to identify the special small metabolites of diseases in cancer and other diseases and has already been widely used in biological studies of humans, plants, and animals [10–14]. Glucose is metabolized via glycolysis into pyruvate, which can either be imported to mitochondria for the TCA cycle, or be catalyzed by lactate dehydrogenase (LDH) to generate lactate. Some amino acids regulate key metabolic pathways necessary for maintenance, growth, and reproduction, revealing the importance of amino acid metabolism [15]. The lysosome is the hub of amino acid sensing. Amino acids are sufficient to have an impact on cellular metabolism with important implications for mammalian target of rapamycin complex 1 (mTORC1), which is regulated by the phosphoinositide 3-kinase (PI3K)-protein kinase B (Akt) signaling pathway [16]. Addition of amino acids rapidly translocates mTORC1 to the lysosomal surface where it interacts with lysosome-associated membrane protein 2 (LAMP2) and the small GTPase ras homolog protein enriched in brain (Rheb) that is regulated by tuberous sclerosis complex (TSC) 1/2. The primary function of TSC1-TSC2 complex is considered as an important negative regulator of mTORC1 activation. In the absence of either TSC1 or TSC2, high levels of Rheb-GTP induce activation of mTORC1, leading to enhanced protein synthesis and cell growth by p70 ribosomal protein kinase 1 (p70S6K1) and ribosomal protein S6 (rpS6) [17–19]. In addition, there is a connection between amino acid metabolism and the TCA cycle. For example, aspartate aminotransferase (AST) promotes the mutual transformation of aspartate and α-ketoglutarate to oxaloacetate and glutamate, affecting the TCA cycle [20].

There’s no metabolic system for the virus. To facilitate virus replication in host cells, viruses can seize organelles to synthesize a large number of metabolites required for viral replication. For instance, Newcastle disease virus (NDV) [21], African swine fever virus (ASFV) [22], influenza A virus (IAV) [23], hepatitis B virus (HBV) [24], and human cytomegalovirus (HCMV) [25] have been proven to alter host cells metabolism for viral replication. Understanding the impact of viral infection on host cell metabolism will promote the knowledge of pathogenic mechanisms and contribute to advancing novel preventative measures that target the metabolism. Recently, the intrinsic mechanisms of the interaction between FMDV and host cell metabolism remain unknown.

In the present study, FMDV-infected PK-15 cells, serum, and tonsils were collected and subjected to metabolomic analysis using ultra-high-performance liquid chromatography/quadrupole time-of-flight tandem mass spectrometry (UHPLC-QTOF-MS, untargeted analysis) or UHPLC-MS (targeted analysis). The serum and tonsils from uninfected pigs were used as a control. The results showed that FMDV infection increased aspartate levels, both *in vitro* and *in vivo*. Aspartate and TCA cycle play important roles in FMDV, EV71, and SVV replication. FMDV, EV71, and SVV enhanced aspartate by promoting protein expression of the solute carrier family 38 member 8 (SLC38A8) transporter. In turn, SLC38A8 promoted FMDV, EV71, and SVV replication *in vitro* and *in vivo*. FMDV-induced increase in amino acids activated PI3K/AKT/TSC2/Rheb/mTOR/p70S6K1 signaling pathway to promote viral replication.

## Results

### A significant change of metabolites was observed in FMDV-infected cells

**Fig 1:**
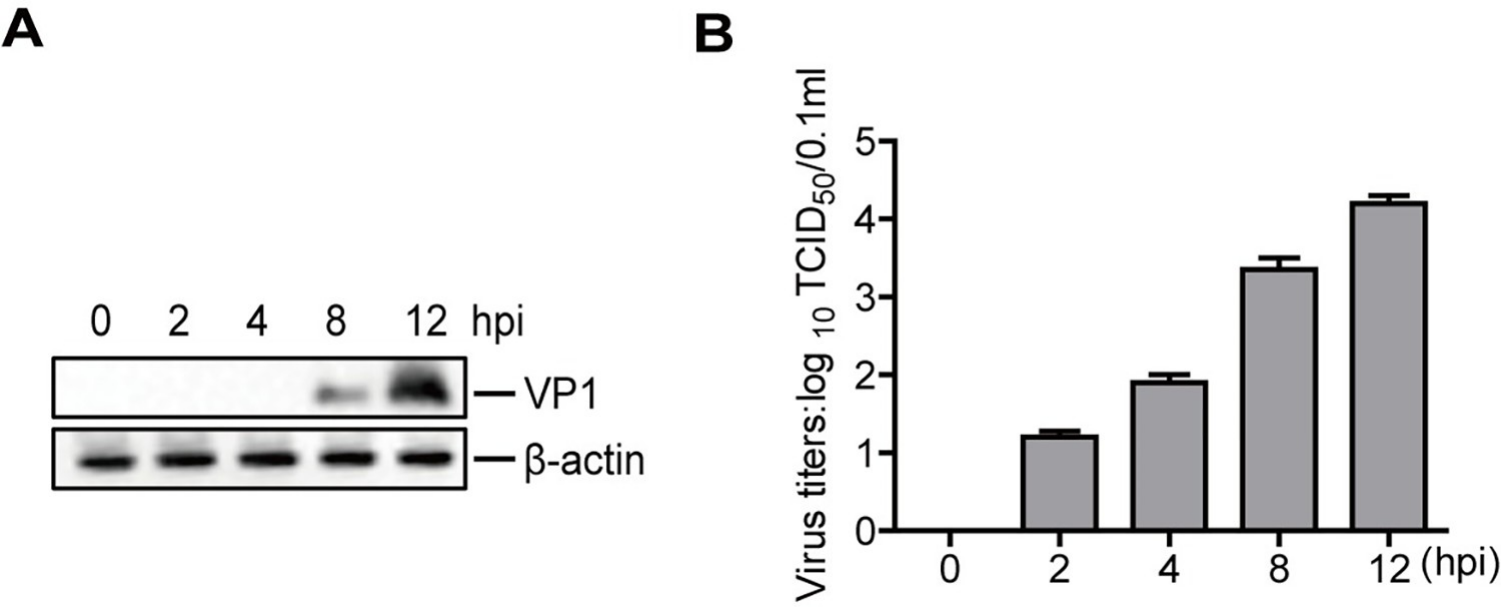
Detection of FMDV replication in PK-15 cells. PK-15 cells were infected with FMDV (MOI 0.5) for 0, 2, 4, 8, and 12 h. Expression of viral VP1 protein was detected by Western blotting (A). The cells treated with PBS were frozen and thawed three times to release the virus and then were centrifuged at 1000 rpm for 5 min. The viral titers in the supernatants were detected by TCID_50_ assay (B). n = 3. Results represent three independent experiments.

To analyze metabolites in PK-15 cells infected with FMDV, we first confirmed FMDV replication in PK-15 cells. The expression of VP1 protein was determined by Western blotting. VP1 protein increased over time (Fig 1A). Viral titers were also significantly upregulated as the infection progressed (Fig 1B). These results indicated the successful replication of FMDV in PK-15 cells.

Electrospray ionization is the source of UHPLC-QTOF-MS and consists of positive (POS) and negative (NEG) ion modes. The valid peaks were matched for 312 POS and 167 NEG metabolites. The ion peaks of metabolites were compared with the standard substances. A total of 284 metabolites were significantly changed during FMDV infection. Of them, 134 metabolites were significantly upregulated and 150 were significantly downregulated compared to the values recorded at 0 h. A significant difference was observed using the heatmap depicting hierarchical clustering of the metabolite data (S1 Fig). The levels of many amino acids were significantly increased, which have important influences on amino acid metabolism. Nucleotide metabolism also showed significant changes. These results indicated that FMDV infection induced significant differential metabolites.

To further confirm the changes in metabolites, the porcine corresponding pathways database was analyzed using the Kyoto Encyclopedia of Genes and Genomes (KEGG) metabolome database and metal analyst. Many altered metabolic pathways were observed during FMDV infection, which was shown using a bubble plot. As a whole, the altered metabolic pathways mainly included alanine, aspartate, and glutamate metabolism, glycine, serine, and threonine metabolism, pyruvate metabolism, and purine metabolism (S2 Fig).

### Analysis of metabolites change in FMDV-infected PK-15 cells

**Fig 2:**
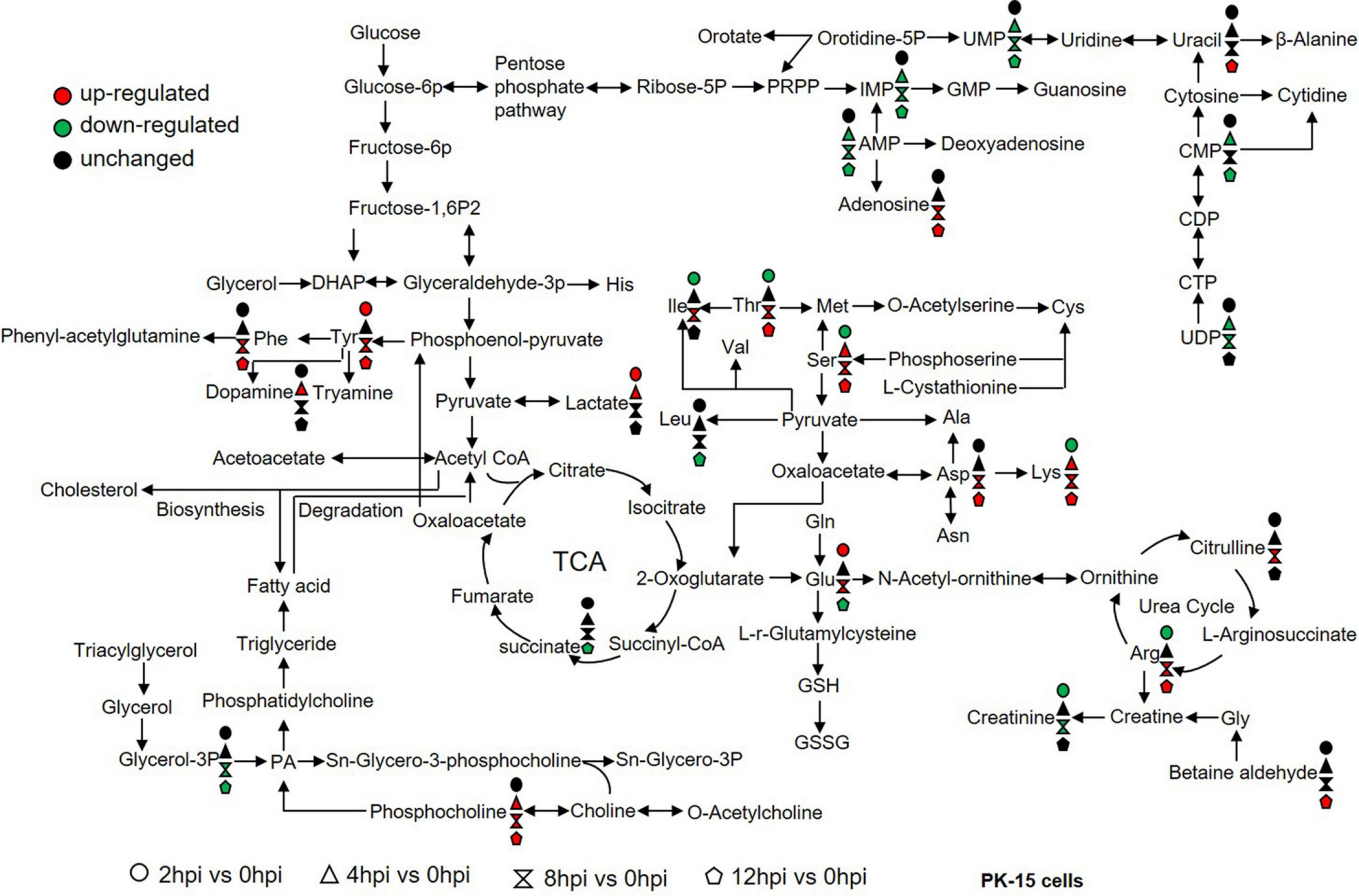
Schematic representation of altered metabolites in FMDV-infected PK-15 cells. Metabolomics of FMDV-infected PK-15 cells was performed using untargeted analysis. The level of aspartate was confirmed using both untargeted and targeted analysis. Many metabolites and associated metabolic pathways were significantly altered after FMDV infection. n = 6 samples at each time point. Fold change ˃1.5 and *p*-value <0.05 were used as the screening criteria for significant differential metabolites. Red, upregulation; green, downregulation.

The detailed metabolite change profiles in PK-15 cells infected with FMDV were obtained. Amino acids, an important substrate, are not only cellular signaling molecules but also regulators of gene expression and protein phosphorylation cascade. At 2 h post-infection (hpi), a few metabolites, including lactate, creatinine, lysine, serine, and tyrosine, were affected. At 4, 8, and 12 hpi, the levels of UMP, IMP, AMP, and CMP were significantly downregulated, and the levels of adenosine, serine, threonine, aspartate, lysine, phosphocholine, and arginine were significantly upregulated, which was consistent with the viral replication process. Meanwhile, at 12 hpi, the level of succinate in the TCA cycle was significantly downregulated. As a whole, FMDV infection significantly affected amino acid metabolism (Fig 2), which allowed them to play important roles in many key metabolic pathways during FMDV infection.

### Analysis of metabolites change in FMDV-infected pigs

The serum from FMDV-infected pigs was collected at 3 and 5 d post-infection (dpi) to analyze metabolite change in pigs. FMDV RNA was detectable in the blood [26]. The detailed metabolite change profiles in serum were also obtained by UHPLC-QTOF-MS. The valid peaks were matched for 200 POS and 206 NEG metabolites. The ion peaks of metabolites were compared with the standard substances. A total of 171 metabolites were significantly changed in FMDV-infected serum. Among them, 66 metabolites were significantly upregulated and 105 were significantly downregulated compared to the values recorded at 0 d.

**Fig 3:**
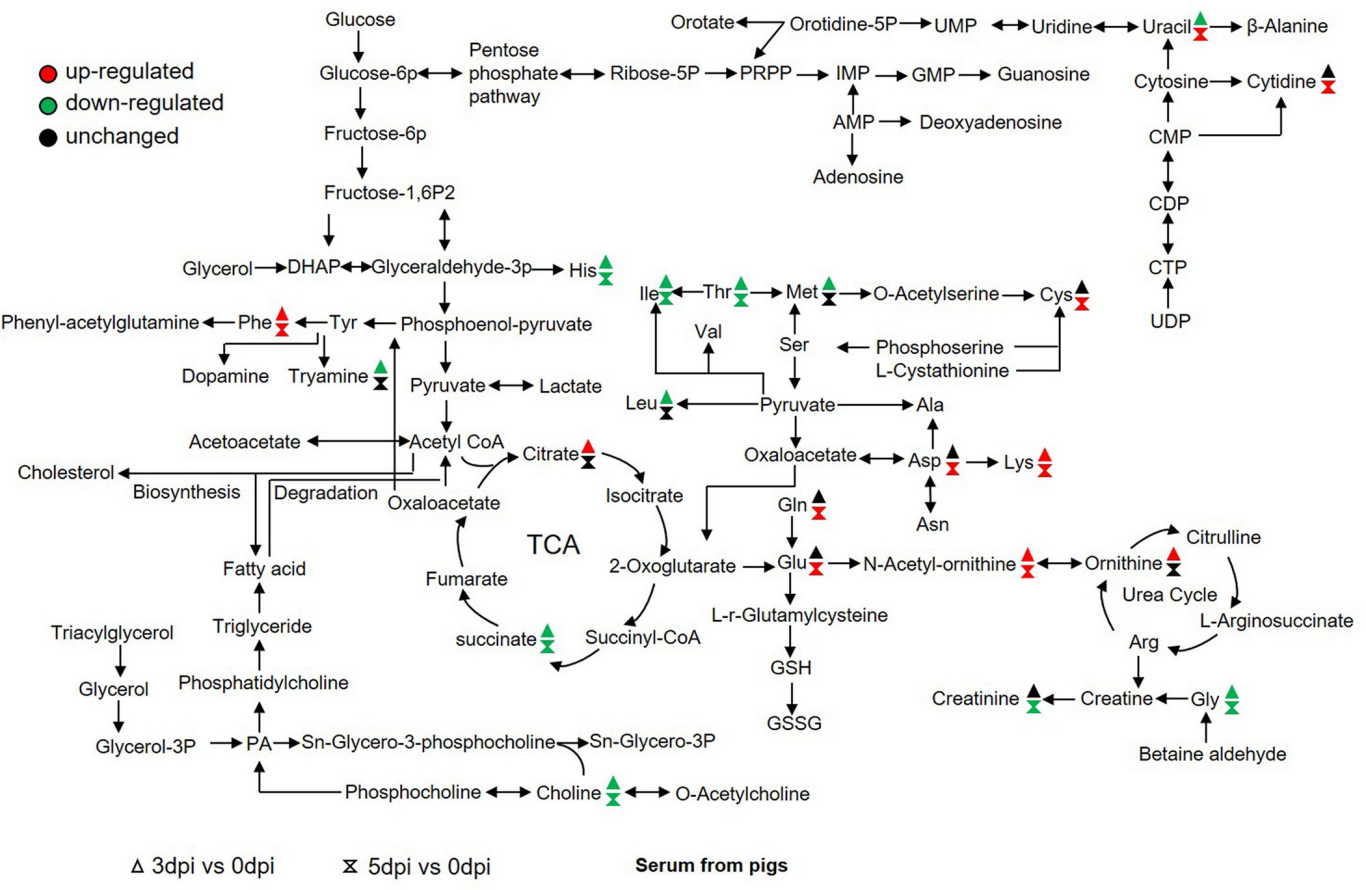
Schematic representation of altered metabolites in serum in FMDV-infected pigs. Metabolomics of serum in FMDV-infected pigs was performed using untargeted analysis. Many metabolites and the associated metabolic pathways were significantly altered in FMDV-infected pigs. n = 6 samples at each time point. Fold change ˃1.5 and *p*-value <0.05 were used as the screening criteria for significant differential metabolites. Red, upregulation; green, downregulation.

As shown in Fig 3, the levels of histidine, isoleucine, threonine, leucine, methionine, glycine, creatinine, choline, tyramine, and succinate were significantly downregulated, while the levels of aspartate, lysine, phenylalanine, glutamate, glutamine, cystine, N-Acetyl-ornithine, ornithine, cytidine, and citrate were significantly upregulated at 3 and/or 5 dpi. As a whole, amino acid metabolism in serum from FMDV-infected pigs was significantly affected, which was following that in FMDV-infected PK-15 cells.

### The amino acid metabolism in tonsils from FMDV-infected pigs

**Fig 4:**
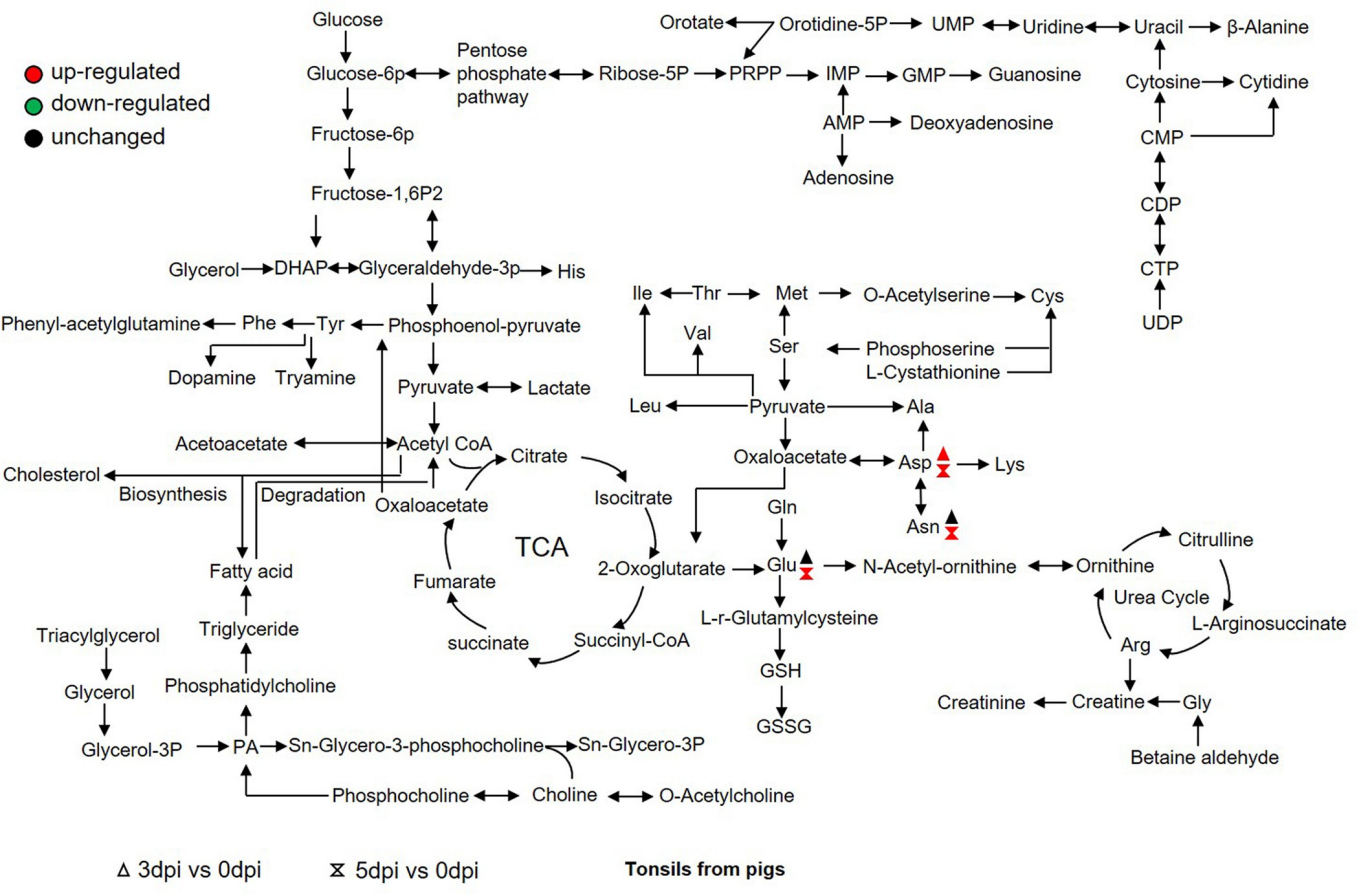
The amino acid metabolism in tonsils in FMDV-infected pigs. The tonsils in the FMDV-infected pigs were collected at 0, 3, and 5 dpi. The number of amino acids in the tonsils was quantified by targeted analysis. Some amino acids were significantly altered in the tonsils after FMDV infection. n = 5 samples at each time point. Red, upregulation; green, downregulation.

The epithelium of oropharyngeal tonsils is one of the critical sites for FMDV replication in pigs during the pre-viremic phase of infection [26,27]. Therefore, the oropharyngeal tonsils from the FMDV-infected pigs were collected, and FMDV RNA was detectable in the submandibular lymph nodes and tonsil tissues [26]. To further analyze the changes in amino acid metabolism during FMDV infection, the number of amino acids in tonsils was quantified. The ion peak area of the amino acid metabolites was detected using UHPLC-MS. The measured value was compared with the standard substance. At 3 dpi, only the level of aspartate was elevated compared to that at 0 dpi. The levels of other amino acids were unchanged. At 5 dpi, the aspartate, glutamate, and asparagine levels were significantly upregulated compared to that at 0 dpi (Figs 4 and S3). Both untargeted and targeted analysis results showed that the level of aspartate was significantly enhanced after FMDV infection. The aspartate may play an important role in FMDV replication.

### Aspartate, glutamate, and the TCA cycle promoted FMDV replication

**Fig 5:**
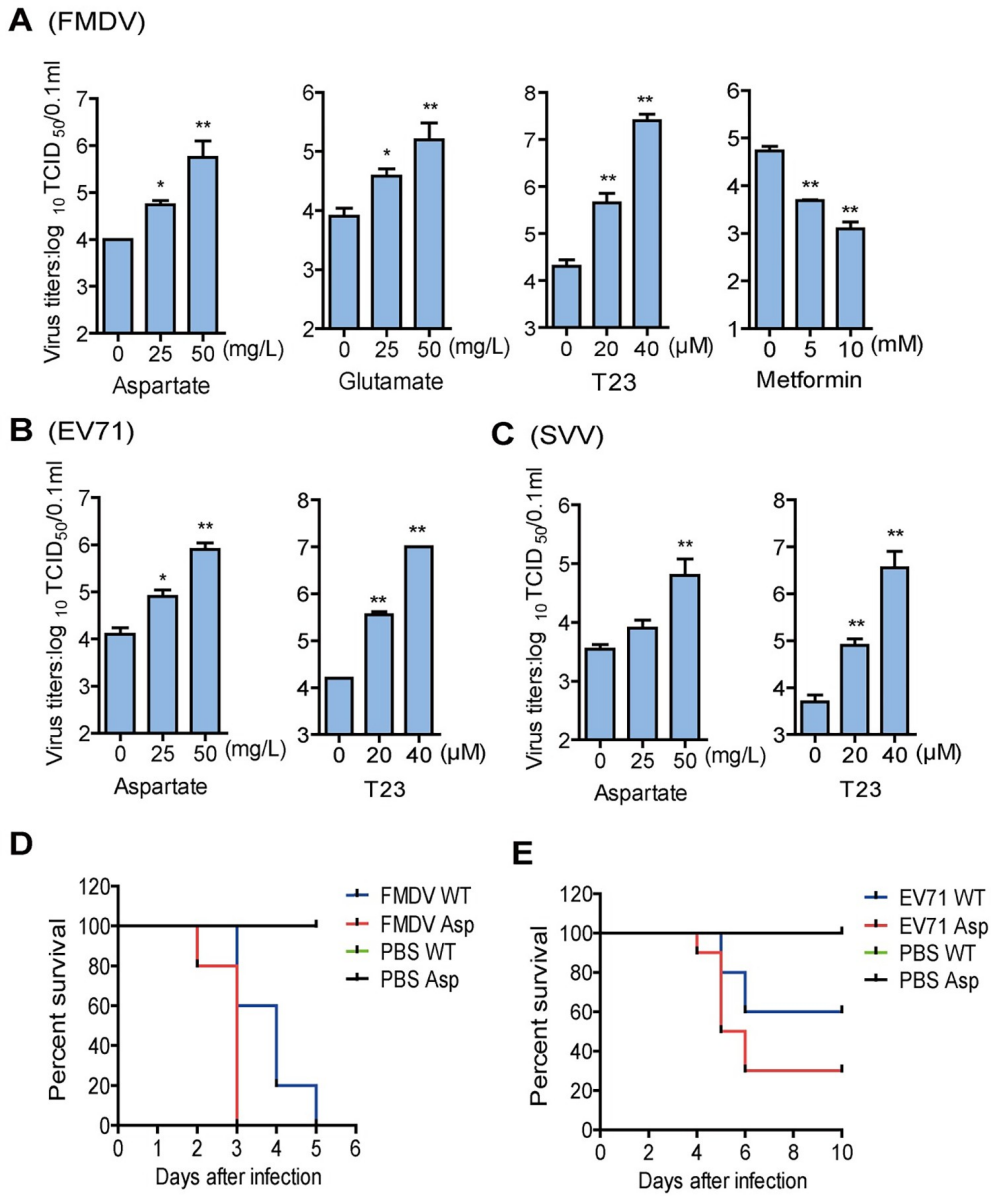
Aspartate and TCA cycle promoted FMDV, EV71, and SVV replication. PK-15 cells were infected with FMDV (MOI 0.5) for 12 h (A) or infected with SVV (MOI 0.5) for 16 h (C), and HEK-293T cells were infected with EV71 (MOI 0.5) for 24 h (B) in the presence of the indicated concentrations of aspartate, glutamate, T23, or metformin. The supernatants were collected and viral titers were detected by TCID_50_ assay. n = 3. Results represent three independent experiments. (D and E) The two-day-old WT mice were subcutaneously mock-injected or injected with 100 mg of aspartate. After 24 h, mice were inoculated with FMDV (10^8^ TCID^50^) (D) or EV71 (10^8^ TCID_50_) (E) at the same sites. The mortality of mice (n = 10 mice per group) was determined. Error bars show standard deviations. *, *P* < 0.05; **, *P* < 0.01.

As described above, FMDV infection induced an increase in aspartate. Thus, we detected the impact of aspartate on FMDV replication. PK-15 cells infected with FMDV were incubated with increasing concentrations of aspartate for 12 h. Viral titers were determined by TCID_50_ assay. Treatment with aspartate promoted FMDV replication in a dose-dependent manner (Fig 5A).

Aspartate aminotransferase (AST), a vitamin B6-dependent enzyme, can promote the mutual transformation of aspartate and α-ketoglutarate to oxaloacetate and glutamate, affecting the TCA cycle [28]. Therefore, the increase in aspartate may further impact glutamate and the TCA cycle. We further detected the impact of glutamate and the TCA cycle on FMDV replication. Tyrphostin 23 (T23) is a well-known inhibitor of protein tyrosine kinases, and the mitochondrial TCA cycle is strongly accelerated after T23 incubation [29]. Metformin treatment elevated lactate production and suppressed the TCA cycle [30]. Thus, T23 and metformin were used to promote and inhibit the TCA cycle in the following experiment. Our results showed that treatment with glutamate and T23 promoted FMDV replication in a dose-dependent manner, while treatment with metformin significantly suppressed FMDV replication in a dose-dependent manner, suggesting that the TCA cycle promoted FMDV replication (Fig 5A).

The family of picornaviridae consists of a variety of RNA viruses, including FMDV, enterovirus 71 (EV71), and Seneca Valley virus (SVV), which are closely related in virion structure and share a similar mechanism of replication [31]. Therefore, we also detected the impact of aspartate and the TCA cycle on EV71 and SVV replication. Treatment with aspartate or T23 significantly promoted EV71 replication in a dose-dependent manner (Fig 5B). Treatment with 25 mg/L of aspartate did not affect SVV replication, while treatment with 50 mg/L of aspartate significantly promoted SVV replication. Treatment with T23 significantly promoted SVV replication in a dose-dependent manner (Fig 5C). The cytotoxicity of T23 and metformin was determined by CCK8 assay. All the used doses of T23 and metformin showed no significant detectable cell death (S4A and S4B Fig). In addition, the impact of aspartate and glutamate on cell growth numbers was investigated using Automated Cell Counter, which showed that the addition of aspartate or glutamate did not increase cell numbers (S5A and S5B Fig).

We determined that SVV did not induce clinical symptoms and death in the mice infection experiments. Thus, we investigated the effect of aspartate on EV71- and FMDV-induced mice mortality by injecting aspartate into mice. The level of aspartate in the mice significantly increased at 24 h after injection (S6 Fig). Mice injected with aspartate were infected with FMDV or EV71. The WT mice infected with FMDV started to die at 3 dpi and all mice died by 5 dpi, while the mice treated with aspartate died more rapidly than FMDV-infected WT mice, which started to die at 2 dpi and all mice died by 3 dpi (Fig 5D). EV71-infected WT mice survived 60%, while EV71-infected mice treated with aspartate only survived 30%, suggesting that treatment with aspartate resulted in higher mortality of the mice infected with EV71 (Fig 5E). Altogether, these results indicated that cellular aspartate plays important roles in FMDV, EV71, and SVV replication.

### FMDV infection enhanced the protein expression of SLC38A8

**Fig 6:**
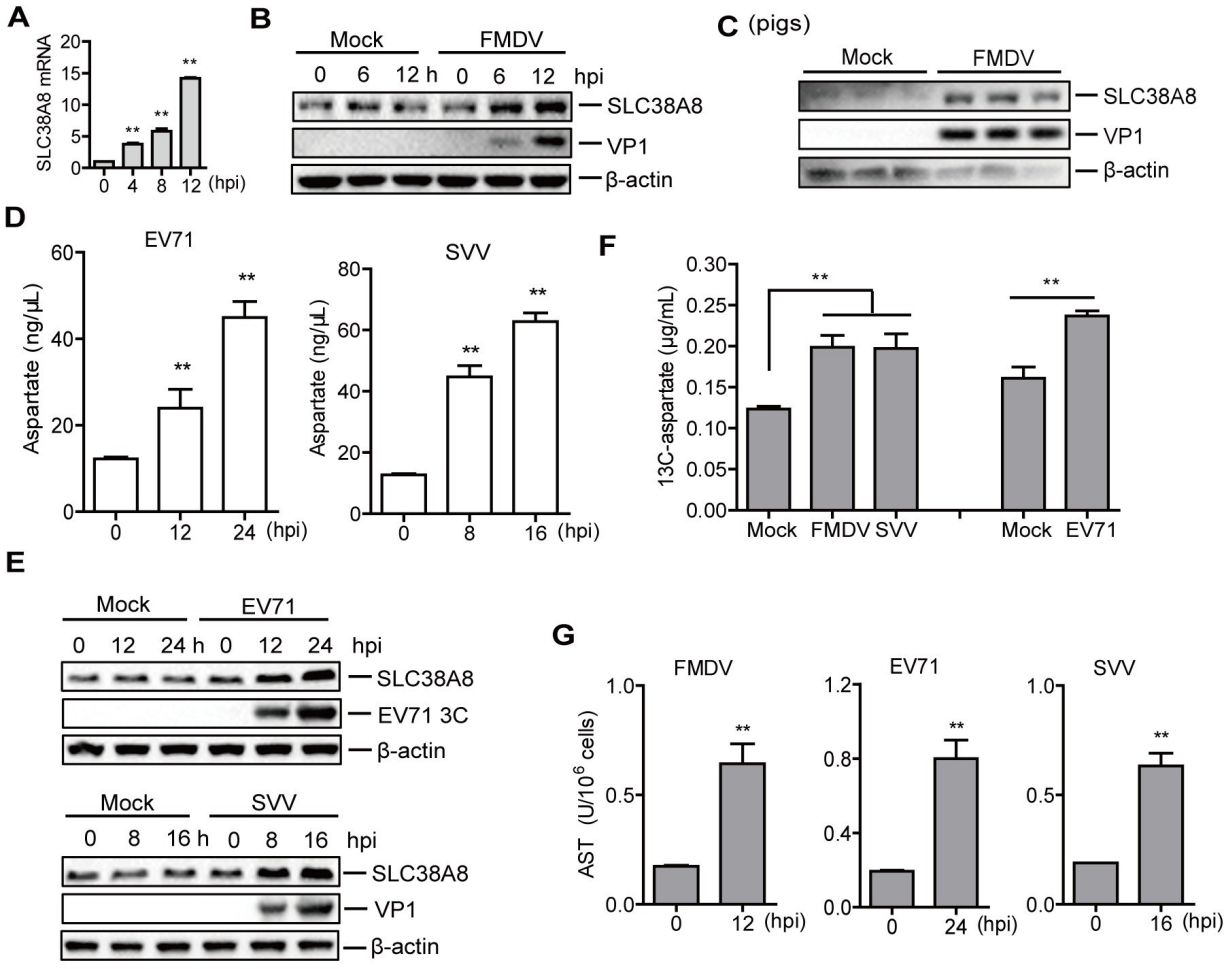
FMDV infection enhanced the protein expression of SLC38A8. (A) PK-15 cells were infected with FMDV (MOI 0.5) for 0, 4, 8, and 12 h. The cells were collected to extract mRNA. The mRNA level of SLC38A8 was determined by qPCR. n = 3. Results represent two independent experiments. (B) PK-15 cells were infected with FMDV (MOI 0.5) for 0, 6, and 12 h. The expression of the SLC38A8 and viral VP1 proteins was determined by Western blotting. (C) The tonsils from pigs that were mock-infected (n = 3) or infected (n = 3) with FMDV were collected. The expression of the SLC38A8 and viral VP1 protein in the tonsils was determined by Western blotting. (D and E) HEK-293T cells were infected with EV71 (MOI 0.5) for 0, 12, and 24 h. PK-15 cells were infected with SVV (MOI 0.5) for 0, 8, and 16 h. The levels of aspartate in the cells were detected using an aspartate detection Kit (D). n = 3. Results represent three independent experiments. Expression of the SLC38A8 and viral VP1 or 3C protein was determined by Western blotting (E). (F) PK-15 cells mock-infected and infected with FMDV (MOI 0.5) or SVV (MOI 0.5) were incubated with ^13^C-labeled aspartate (2 mM) for 12 h. HEK-293T cells mock-infected and infected with EV71 (MOI 0.5) were incubated with ^13^C-labeled aspartate (2 mM) for 24 h. The levels of ^13^C-aspartate in cells were detected and compared. n = 3. Results represent three independent experiments. (G) PK-15 cells were infected with FMDV (MOI 0.5) for 12 h (A) or infected with SVV (MOI 0.5) for 16 h. HEK-293T cells were infected with EV71 (MOI 0.5) for 24 h. The AST activity was determined by the kit. n = 3. Results represent three independent experiments. Error bars show standard deviations. *, *P* < 0.05; **, *P* < 0.01.

Amino acid transporters are membrane-bound solute carriers (SLC) transport proteins responsible for transporting amino acids into and out of cells or cellular organelles. Dysregulation of AATs leads to metabolic reprogramming and changes in intracellular amino acid levels [32]. Aspartate transporters contain a large number of proteins, including SLC1A1, SLC1A2, SLC1A3, SLC1A6, SLC1A7, SLC25A12, SLC25A13,SLC38A7, and SLC38A8 [33,34]. To explore the mechanism by which FMDV infection promotes the increase of aspartate, the mRNA expression of aspartate transporters was detected to screen amino acid transporters involved in aspartate metabolism during viral infection. FMDV infection promoted the mRNA expression of SLC38A8 (Fig 6A), while it did not affect the mRNA levels of SLC1A1, SLC1A2, SLC1A3, SLC1A6, SLC1A7, SLC25A12, SLC25A13, and SLC38A7 transporters (S7 Fig). Therefore, the protein level of SLC38A8 was further detected, which indicated that FMDV infection also promoted the protein expression of SLC38A8 in both PK-15 cells (Fig 6B) and pigs (Fig 6C).

As described above, cellular aspartate promoted EV71 and SVV replication. Therefore, we also detected the aspartate and the protein expression of SLC38A8 during EV71 and SVV infection. The level of aspartate was significantly increased during EV71 or SVV infection (Fig 6D). Meanwhile, EV71 and SVV enhanced the protein expression of SLC38A8 as the infection progressed (Fig 6E). The abundance of the proteins in Fig 6B and 6C, and 6E was quantified using ImageJ Software (S8A–S8C Fig).

To further investigate the impact of viral infection on aspartate uptake, we detected the aspartate uptake during infection using ^13^C-labeled aspartate. The results showed that the level of ^13^C-aspartate was significantly enhanced in FMDV-, EV71-, or SVV-infected cells compared to mock-infected cells (Fig 6F), suggesting that FMDV, EV71, and SVV infection promoted aspartate uptake.

The upregulated aspartate may lead to a reversible transition between the TCA cycle and amino acids synthesis using AST (aspartate aminotransferase, a vitamin B6-dependent enzyme). Therefore, the activity of AST was identified during FMDV, EV71, and SVV infection. The results showed that the AST activity was significantly enhanced in the FMDV-, EV71-, and SVV- infected cells compared to that in the mock-infected cells (Fig 6G). Altogether, these results indicated that FMDV, EV71, and SVV infection promoted the protein expression of the SLC38A8 transporter and enhanced the activity of AST. The level of aspartate was significantly enhanced after EV71 and SVV infection.

### SLC38A8 was responsible for FMDV-induced aspartate *in vitro*

**Fig 7:**
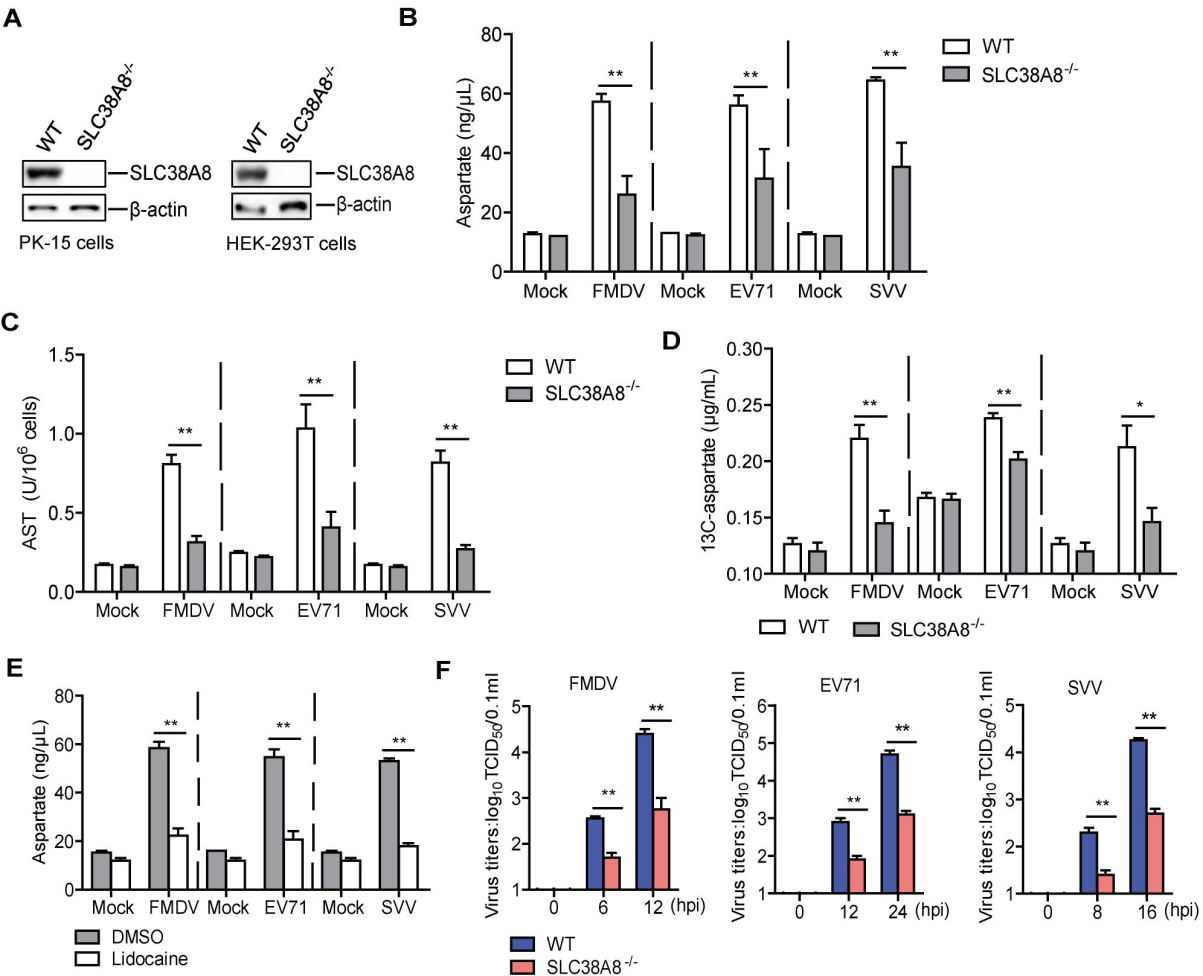
SLC38A8 was responsible for FMDV-induced aspartate *in vitro*. (A) The expression of SLC38A8 in the WT and SLC38A8^-/-^ PK-15 cells or HEK-293T cells was detected by Western blotting. (B and C) WT and SLC38A8^-/-^ PK-15 cells were mock-infected or infected with FMDV (MOI 0.5) for 12 h or SVV (MOI 0.5) for 16 h. WT and SLC38A8^-/-^ HEK-293T cells were mock-infected or infected with EV71 (MOI 0.5) for 24 h. The level of aspartate in the cells was detected using an aspartate detection Kit (B). The activity of AST was determined by kit (C). n = 3. Results represent three independent experiments. (D) WT and SLC38A8^-/-^ PK-15 cells infected with FMDV (MOI 0.5) or SVV (MOI 0.5) were incubated with ^13^C-labeled aspartate (2 mM). WT and SLC38A8^-/-^ HEK-293T cells infected with EV71 (MOI 0.5) were incubated with ^13^C-labeled aspartate (2 mM). The levels of ^13^C-aspartate in cells were detected and compared. n = 3. Results represent three independent experiments. (E) PK-15 cells were infected with FMDV (MOI 0.5) for 12 h or SVV (MOI 0.5) for 16 h, and HEK-293T cells were infected with EV71 (MOI 0.5) for 24 h in the absence and presence of the lidocaine (100 μM). The level of aspartate in the cells was detected using an aspartate detection Kit. n = 3. Results represent three independent experiments. (F) WT and SLC38A8^-/-^ PK-15 cells were infected with FMDV (MOI 0.5) for 0, 6, and 12 h or SVV (MOI 0.5) for 0, 8, and16 h. WT and SLC38A8^-/-^ HEK-293T cells were infected with EV71 (MOI 0.5) for 0, 12, and 24 h. The viral titers in the supernatants were detected by TCID_50_ assay. n = 3. Results represent three independent experiments. Error bars show standard deviations. *, *P* < 0.05; **, *P* < 0.01.

To determine the impact of SLC38A8 on FMDV-, EV71-, and SVV-induced aspartate, we established the SLC38A8^-/-^ PK-15 and HEK-293T cells (Fig 7A). The knockout of SLC38A8 did not affect cell growth numbers compared to the WT cells (S5C Fig). WT and SLC38A8^-/-^ PK-15 cells were mock-infected or infected with FMDV and SVV, and WT and SLC38A8^-/-^ HEK-293T cells were mock-infected or infected with EV71. The cells were collected to detect the aspartate and the AST activity. FMDV-, EV71-, and SVV-induced aspartate and the AST activity were significantly decreased in the SLC38A8^-/-^ cells compared to that in the WT cells (Fig 7B and 7C).

To further determine the impact of SLC38A8 on FMDV-, EV71-, and SVV-induced aspartate uptake, ^13^C-labeled aspartate uptake in WT and SLC38A8^-/-^ cells was assessed during viral infection. There was no change in ^13^C-labeled aspartate level in mock-infected WT and SLC38A8^-/-^ cells, while the level of ^13^C-labeled aspartate was significantly decreased in FMDV-, EV71-, or SVV-infected SLC38A8^-/-^ cells compared to that of FMDV-, EV71-, or SVV-infected WT cells (Fig 7D).

SLC38A8 transports aspartate using a Na^+^-dependent transport mechanism [34]. Lidocaine, a Na^+^ channels inhibitor [35], was selected to block the Na^+^ channels for suppressing the SLC38A8 functions. All doses of lidocaine used in this study did not induce significant detectable cell death (S3C Fig). Cells incubated with DMSO or lidocaine were infected with FMDV, EV71, and SVV. The level of aspartate was determined using an aspartate detection kit. FMDV-, EV71-, and SVV-induced aspartate decreased in the lidocaine-treated cells compared to those in the DMSO-treated cells (Fig 7E).

The impact of SLC38A8 on FMDV, EV71, and SVV replication was also evaluated. WT and SLC38A8^-/-^ cells were infected with FMDV, EV71, and SVV. At the indicated time points, viral titers were assessed and compared. FMDV, EV71, and SVV titers were significantly reduced in the SLC38A8^-/-^ cells than that in the WT cells (Fig 7F). Altogether, these results indicated that the SLC38A8-mediated Na^+^-dependent transport mechanism was involved in FMDV-, EV71-, and SVV-induced aspartate. SLC38A8 promoted FMDV, EV71, and SVV replication in cells.

### SLC38A8 was responsible for FMDV-induced aspartate *in vivo*

**Fig 8:**
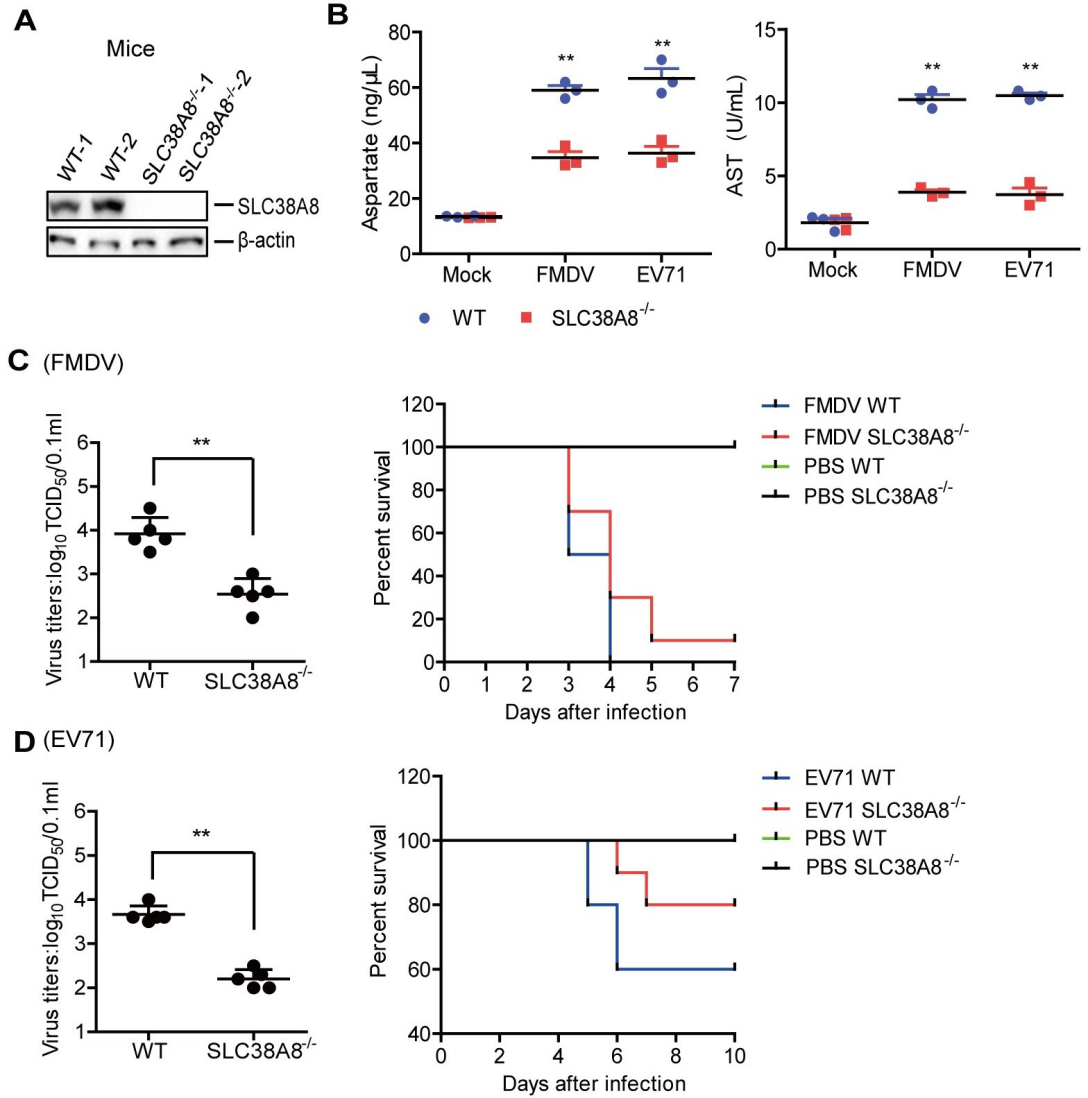
SLC38A8 was responsible for FMDV-induced aspartate *in vivo*. (A) The expression of SLC38A8 in the WT and SLC38A8^-/-^ mice was detected by Western blotting. (B) The three-day-old WT and SLC38A8^-/-^ mice (n = 3) were subcutaneously infected with FMDV (10^8^ TCID^50^) or EV71 (10^8^ TCID_50_). At 2 dpi, The levels of aspartate and the activity of AST in the serum were determined by kit. (C and D) The three-day-old WT and SLC38A8^-/-^ mice were subcutaneously infected with FMDV (10^8^ TCID^50^) (C) or EV71 (10^8^ TCID_50_) (D). At 2 dpi, the viral titers in the mice carcasses without the head, tail, limbs, and viscerawere detected using TCID_50_ assay (n = 5). The mortality of mice (n = 10) was determined. Error bars show standard deviations. *, *P* < 0.05; **, *P* < 0.01.

We further investigated the function of SLC38A8 on FMDV-, EV71-, and SVV-induced aspartate in SLC38A8^-/-^ mice. The deficiency of SLC38A8 protein in SLC38A8^-/-^ mice was confirmed by western blotting (Fig 8A). The abundance of SLC38A8 was quantified using ImageJ Software (S8D Fig). WT and SLC38A8^-/-^ mice were infected with FMDV or EV71. The level of aspartate and the AST activity were assessed and compared. Similar to the results in cells, FMDV- and EV71-induced aspartate levels and the AST activity were significantly decreased in the SLC38A8^-/-^ mice compared to that in the WT mice (Fig 8B).

The impact of SLC38A8 on FMDV and EV71 replication in mice and FMDV- and EV71-induced mice mortality was evaluated as well. FMDV and EV71 titers were significantly decreased in the SLC38A8^-/-^ mice compared to that in the WT cells (Fig 8C and 8D, left). The WT mice infected with FMDV started to die at 3 dpi and all mice died by 4 dpi, while the SLC38A8^-/-^ mice infected with FMDV started to die at 3 dpi and survived 30% at 4 dpi and survived 10% at 7 dpi, indicating that SLC38A8 deficiency decelerated FMDV-induced the death of mice (Fig 8C, right). The WT mice infected with EV71 survived 60%, while the SLC38A8^-/-^ mice infected with EV71 survived 80% at 10 dpi, suggesting that SLC38A8 deficiency resulted in lower mortality of the mice infected with EV71 (Fig 8D, right). Altogether, these results indicated that SLC38A8 was involved in FMDV- and EV71-induced aspartate in mice. SLC38A8 promoted FMDV and EV71 replication *in vivo*.

### FMDV infection promoted the transfer of mTOR to the lysosome and enhanced interaction between mTOR and Rheb

**Fig 9:**
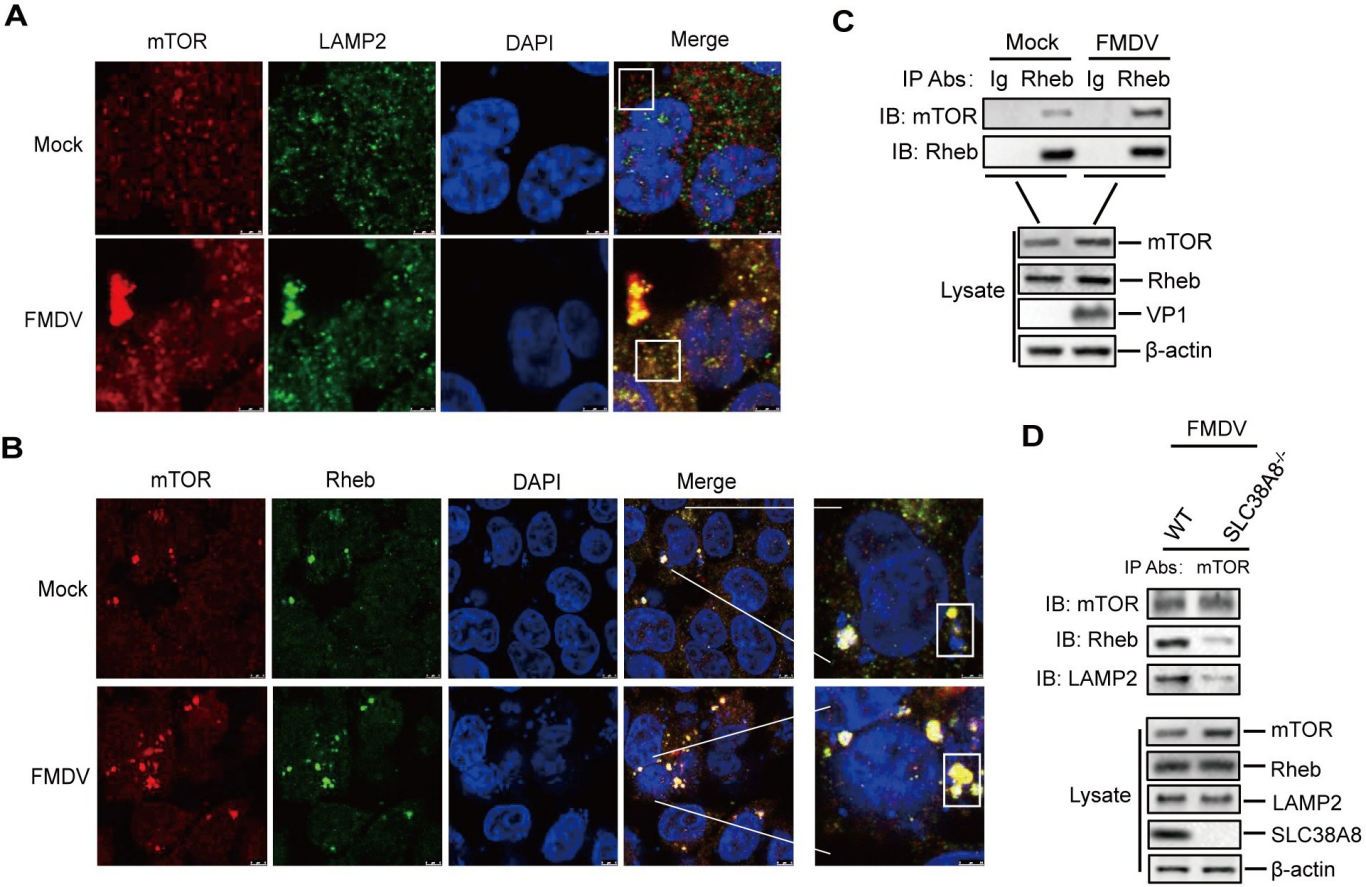
FMDV infection enhanced interaction between mTOR and Rheb. (A and B) PK-15 cells were mock-infected or infected with FMDV (0.5 MOI) for 12 h. The expression of mTOR, LAMP2, and Rheb was detected by IFA. Cells were double-immunostained for LAMP2 (green), Rheb (green), and mTOR (red); cellular nuclei were counterstained with 4’,6-diamidino-2-phenylindole (DAPI) (blue). The plots within the white boxes were used for Pearson’s co-localization coefficient analysis using ImageJ Software. (C and D) PK-15 cells were mock-infected or infected with FMDV (0.5 MOI) for 12 h. The cell lysates were immunoprecipitated with anti-Rheb (C) or anti-mTOR (D) antibodies, and then subjected to Western blotting analysis.

The lysosome is a hub of amino acid sensing. Amino acids can stimulate mTORC1 activity by translocating mTOR to the lysosomal surface where it interacts with the LAMP2 (a marker for lysosomes) and small GTPase Rheb, resulting in the activation of Rheb and mTORC1 [16,36,37]. FMDV infection induced increase of a large number of amino acids. However, whether FMDV-induced upregulation of amino acids also enhanced mTORC1 activity is unclear. Therefore, we detected the intracellular localization of mTOR by indirect immunofluorescence assay (IFA) during FMDV infection. No obvious interaction between mTOR and LAMP2 was observed in the mock-infected cells (Pearson’s co-localization coefficient: 0.7). FMDV infection promoted the transfer of mTOR to the lysosome, and the interaction between mTOR and LAMP2 was observed (Pearson’s co-localization coefficient: 0.91) (Fig 9A). Further studies showed an interaction between mTOR and Rheb in the mock-infected cells (Pearson’s co-localization coefficient: 0.95), and FMDV infection enhanced the interaction between mTOR and Rheb (Pearson’s co-localization coefficient: 0.98) (Fig 9B).

We further investigate the interaction between mTOR and Rheb in the context of viral infection using a coimmunoprecipitation assay. PK-15 cells were mock-infected and infected with FMDV. The cell lysates were immunoprecipitated with anti-Rheb antibody and analyzed by Western blotting. The results showed that the interaction between mTOR and Rheb was enhanced in the FMDV-infected cells compared to that in the mock-infected cells (Fig 9C). Additionally, FMDV-induced interaction between mTOR and Rheb was reduced in the SLC38A8^-/-^ cells compared to the WT cells (Fig 9D). The abundance of the proteins in Fig 9 was quantified by densitometric analysis using ImageJ Software (S9 Fig). Altogether, these results indicated that FMDV-induced upregulation of amino acids promoted the transfer of mTOR to the lysosome and increased interaction between mTOR and Rheb.

### FMDV infection activated PI3K/AKT/TSC2/Rheb/mTOR/p70S6K1 signaling axis to promote viral replication

**Fig 10:**
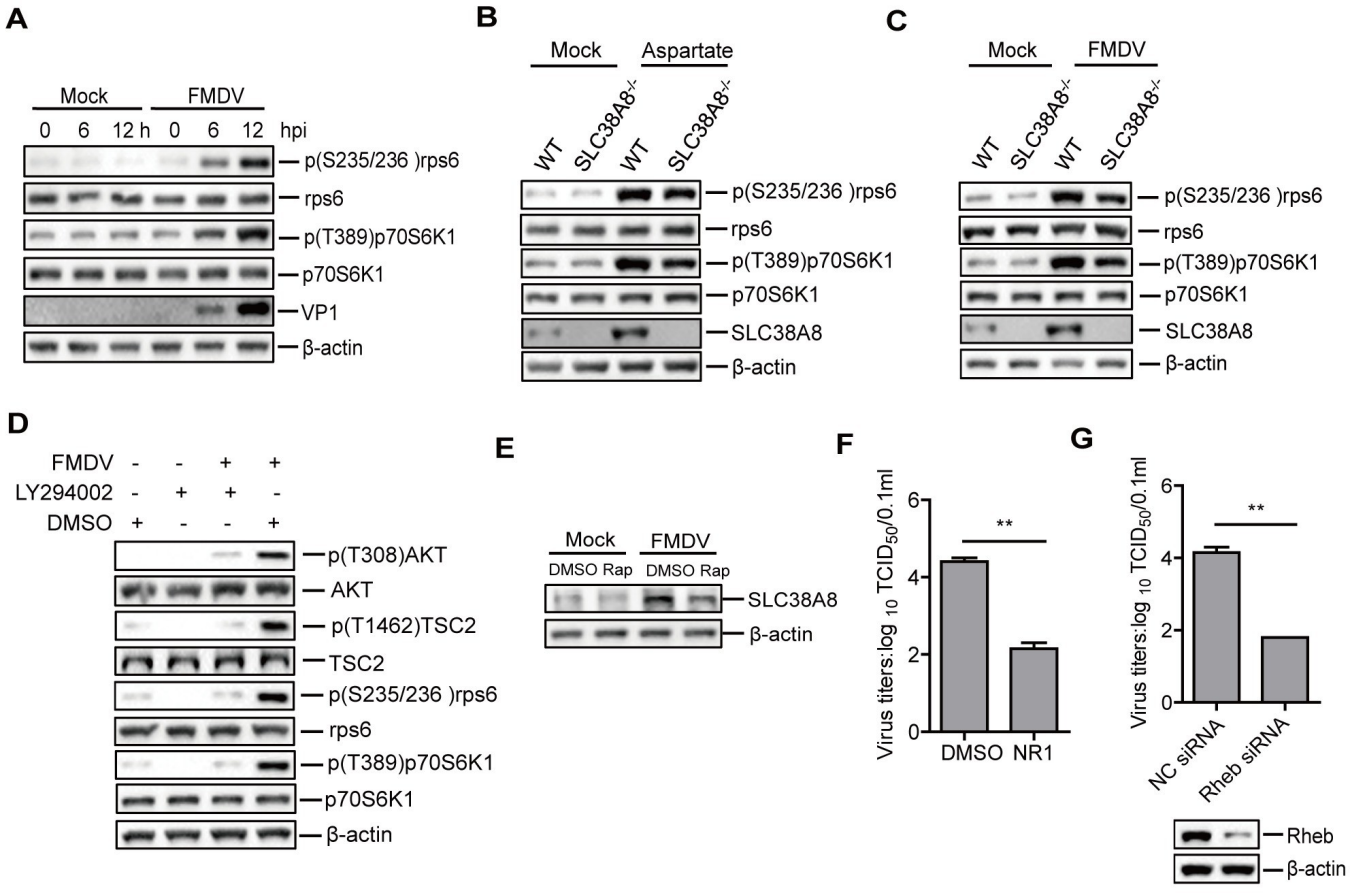
FMDV infection activated PI3K/AKT/TSC2/Rheb/mTOR/p70S6K1 signaling axis to promote viral replication. (A) PK-15 cells were mock-infected or infected with FMDV (0.5 MOI) for 0, 6, and 12 h. Expression of the p70S6K1, p(T389)p70S6K1, rps6, p(S235/236) rps6, and viral VP1 protein was determined by Western blotting. (B and C) WT and SLC38A8^-/-^ PK-15 cells were incubated with aspartate (B) or infected with FMDV for 12 h (C). The cells were collected and expression of the SLC38A8, p70S6K1, p(T389)p70S6K1, rps6, and p(S235/236) rps6 protein was determined by Western blotting. (D) PK-15 cells were mock-infected or infected with FMDV (0.5 MOI) in the absence and presence of PI3K specific inhibitor LY294002 (20 μM). The cells were collected and subjected to Western blotting analysis. (E) Mock-infected and FMDV-infected PK-15 cells were incubated with DMSO or rapamycin (2 μM), and expression of the SLC38A8 was determined by Western blotting. (F) PK-15 cells were infected with FMDV (0.5 MOI) in the absence and presence of Rheb-specific inhibitor NR1 (30 μM). The viral titers in the supernatants were detected by TCID_50_ assay. n = 3. Results represent three independent experiments. (G) PK-15 cells were transfected with 250 nM of Rheb siRNA or NC siRNA. At 36 h after transfection, cells were infected with FMDV (0.5 MOI) for 12 h. The viral titers in the supernatants were detected by TCID_50_ assay. n = 3. Results represent three independent experiments. Error bars show standard deviations. *, *P* < 0.05; **, *P* < 0.01.

p70 ribosomal protein kinase 1 (p70S6K1) and ribosomal protein S6 (rpS6) are two downstream effectors of the mTORC1 signaling network, which integrate nutrient and mitogen signals to regulate protein translation, cell growth, cell size, and cell division [38]. The phosphorylation of p70S6K1 and rpS6 is necessary to maintain their function [39]. In the present study, we detected the status of p70S6K1 and rpS6 in the mock-infected- and FMDV-infected cells. FMDV infection did not affect the protein expression of p70S6K1 and rpS6, while it enhanced the phosphorylation of p70S6K1 and rpS6 as the infection progressed (Fig 10A). Amino acid stimuli can promote the phosphorylation of p70S6K1 and rpS6, resulting in an increase in protein synthesis [40]. Our results indicated that the addition of aspartate triggered the phosphorylation of p70S6K1 and rpS6, and the phosphorylation of p70S6K1 and rpS6 was decreased in the SLC38A8^-/-^ cells compared to that in the WT cells (Fig 10B). Meanwhile, in the FMDV-infected cells, the phosphorylation of p70S6K1 and rpS6 was also decreased in the SLC38A8^-/-^ cells compared to that in the WT cells (Fig 10C), suggesting that FMDV-induced the upregulation of aspartate was responsible for the increase of the phosphorylation of p70S6K1 and rpS6.

As described above, amino acid stimulation enhanced interaction between Rheb and mTOR, resulting in the activation of the mTOR/p70S6K1 signaling axis. However, the upstream pathway by which Rheb is activated remains unclear. On the lysosomal membrane, the growth-factor-induced PI3K-Akt pathway phosphorylates TSC2, the GTPase-activating protein (GAP) for Rheb, and induces TSC2 dissociation from the lysosome, providing a permissive condition for Rheb activation and thereby resulting in the activation of mTORC1 [41–43]. Therefore, the PI3K/Akt/TSC2/Rheb pathway was examined during FMDV infection to determine the upstream intracellular signaling pathways involved in the activation of Rheb. The results showed that FMDV infection promoted the phosphorylation of AKT, TSC2, p70S6K1, and rpS6, while the addition of LY294002, a potent and specific inhibitor of PI3K, inhibited FMDV-induced phosphorylation of AKT, TSC2, p70S6K1 and rpS6 (Fig 10D), suggesting inhibition of Akt/TSC2/Rheb pathway by PI3K inhibitor. These results indicated that the upstream intracellular PI3K/Akt/TSC2/Rheb pathway was involved in mTOR/p70S6K1 activation in the FMDV-infected cells.

The mTORC1 signaling network is associated with amino acid transporters gene expression [44]. Therefore, the impact of the mTORC1 pathway on FMDV-induced SLC38A8 protein expression was evaluated using rapamycin, a specific inhibitor of mTOR. The results showed that adding rapamycin inhibited FMDV-induced SLC38A8 protein expression (Fig 10E), suggesting that FMDV promoted the expression of SLC38A8 by the mTORC1 signaling pathway.

The Rheb/mTOR/p70S6K1 pathway plays an important role in protein translation, which may be involved in FMDV replication. The GTPase activity of Rheb is essential for the mTOR/p70S6K1 pathway and cellular survival [45]. Therefore, the impact of Rheb on FMDV replication was assessed using Rheb inhibitor NR1 [46], suggesting that FMDV replication was significantly decreased in the NR1-treated cells compared to that in the DMSO-treated cells (Fig 10F). In addition, FMDV replication was significantly inhibited in the Rheb siRNA-transfected cells compared to that in the NC siRNA-transfected cells (Fig 10G). The addition of LY294002, rapamycin, or NR1 did not induce significant detectable cell death (S4D–S4F Fig). The abundance of the proteins in Fig 10 was quantified by densitometric analysis using ImageJ Software (S10 Fig). Altogether, these results indicated that FMDV induced the activation of the PI3K/AKT/TSC2/Rheb pathway, and the upregulation of amino acids enhanced the interaction of mTOR-Rheb to activate the mTOR/p70S6K1 signaling axis to promote protein translation for viral replication.

## Discussion

Recently, metabolomics has already been widely used to understand metabolites change during viral infection. For instance, Newcastle disease virus (NDV) degrades SIRT3 via PINK1-PRKN-dependent mitophagy to enhance glycolysis to promote viral replication [21]; classical swine fever virus (CSFV) infection decreases pyruvate levels while promoting lactate release in pigs and PK-15 cells, and lactate dehydrogenase B inhibition promoted CSFV growth via mitophagy [47]; African swine fever virus (ASFV) infection promotes host TCA cycle and induces an increase in lactate level, resulting in low expression of IFN-β and increased ASFV replication [22]; and glycolysis is significantly altered during dengue virus (DENV) infection, and pharmacologically inhibiting the glycolysis reduced DENV RNA synthesis and infectious virion production [48]. Although viruses can regulate cellular metabolism to promote their replication, different viruses regulate cell metabolism in different ways. It can be used to identify metabolites in diseases and has become an important strategy for further screening biomarker molecules and designing novel vaccines targeting the altered metabolic pathways. Here, we first analyzed the metabolomic profiles of FMDV-infected PK-15 cells and pigs using UHPLC-QTOF-MS or UHPLC-MS. There were significant changes in amino acid metabolism, which partly reflect the intracellular contributions to understanding how FMDV regulates host amino acids to promote self-replication. Our data provide new viewpoints on the FMDV-host interactions, which may help understand the infection and pathogenesis of FMDV.

Glycolysis, TCA cycle, and lipid metabolism are intracellular energy metabolism, which plays important roles in energy balance, body diseases and viral replication [49]. For instance, the lipid exchange promotes the formation of virus factories during ASFV infection, revealing the importance of lipid metabolism in ASFV infection [50]; all the steps of the hepatitis C virus (HCV) life cycle are affected by host lipid metabolism [51,52]; glycolysis and glutaminolysis, but not fatty acid β-oxidation, are essential energy sources for Marek’s disease virus (MDV) replication [53]; Vaccinia virus (VACV) infection promotes TCA cycle independent of glutaminolysis, and the increase of citrate level depends on VACV-encoded viral growth factor (VGF) and STAT3 signaling [54]; during human cytomegalovirus (HCMV) infection, glucose carbon is not completely inhibited by TCA cycle for energy, and glutamine is converted to α-ketoglutarate to maintain the TCA cycle and ATP production for viral replication, confirming the importance of the TCA cycle in HCMV replication [55]. However, the TCA cycle is not essential for infectious spleen and kidney necrosis virus (ISKNV) replication [56]. These results showed that the TCA cycle plays multiple roles in viral replication. In the present study, our results showed that promotion of the TCA cycle by T23 significantly facilitated FMDV, EV71, and SVV replication, revealing the importance of the TCA cycle in picornavirus infection. However, our data showed that FMDV infection did not significantly affect metabolites in the TCA cycle using untargeted analysis. Therefore, the interaction between FMDV infection and the TCA cycle deserves further investigation in future studies.

In addition to glycolysis, TCA cycle, and lipid metabolism, amino acid metabolism is also important in cells. Amino acids are used to synthesize proteins and intermediate metabolites of the biosynthetic pathways. They are classified as nutritionally essential or nonessential for animals and humans. Metabolic disorders of amino acids contribute to plenty of diseases [57]. The impact of viral infection on amino acid metabolism has been described. For example, SARS-CoV-2 infection alters tryptophan, arginine and glutamine metabolisms to promote viral replication [58]; amino acid metabolism is affected in Kaposi’s sarcoma-associated herpesvirus (KSHV)-infected cells, and KSHV infection induces mondoA, a Myc family member, to promte glutamine uptake and glutaminolysis to produce TCA cycle intermediates [59,60]. NDV infection alters the amino acid metabolic pathway, and the levels of isoleucine, serine, tyrosine, methionine, threonine, and alanine are significantly promoted, suggesting that these amino acids may play an important role in NDV replication [61]. In the present study, we found that the level of aspartate was significantly upregulated both in cells and in pigs during FMDV infection. Detailed analyses determined that aspartate promoted FMDV, EV71, and SVV replication *in vitro and in vivo*, demonstrating that the increased amount of aspartate contributed to the rapid FMDV, EV71, and SVV replication. Addition of aspartate did not cause a strong promotion effect on the death of mice infected with FMDV, which may be due to that a high dose of FMDV was used in the infection assay or the limited absorption of aspartate to the tissues in mice. We have analyzed the amino acids composition of viral proteins of FMDV, EV71, and SVV, which showed that the amount of aspartate is abundant (S11 Fig), suggesting that viral protein synthesis requires a large number of aspartate. In addition to being a constituent of proteins, aspartate serves as a central building block for many metabolic processes, such as the biosynthesis of other amino acids, nucleotides, nicotinamide adenine dinucleotide (NAD), intermediate metabolites of the TCA cycle and glycolysis pathway [62,63]. The virus might hijack the aspartate supplies for virion production, resulting in the inhibition of activation of host antiviral response. These may be the reasons why aspartate is important for picornavirus replication. In addition, the concentrations of exogenous aspartate and glutamate used in this study were similar to the aspartate (approximately 0.03–1.3 mM) and glutamate (approximately 0.19–1 mM) physiological concentrations [64,65], which could represent the impact of aspartate and glutamate on viral replication. The levels of glutamate and aspartate were significantly changed in EV71-infected Vero cells, and EV71 infection induced the decrease of aspartate [66]. Our data indicated that EV71 infection enhanced aspartate, which was inconsistent with previous results [66]. The difference in aspartate may be due to different cells.

Amino acid transporters are membrane-bound SLC transporters and approximately 430 SLCs have been reported. Of them, SLC1, SLC3, SLC6, SLC7, SLC15, SLC17, SLC18, SLC25, SLC32, SLC36, and SLC38 have been identified as amino acid transporters [67]. The SLC38 family contains 11 members, SLC38A1-SLC38A11. SLC38A8, also known as SNAT8, prefers to transport arginine, alanine, histidine, glutamine, and aspartate using a Na^+^-dependent transport mechanism [34]. Most amino acid transporters show tissue-specific and developmental stage-specific expression in normal cells. However, many tumor cells express high levels of specific amino acid transporters to meet the rapid growth of cells [67]. Deleting some amino acid transporters can inhibit the growth of tumor cells, suggesting that amino acid transporters may be a therapeutic target for cancer [68]. The association between viruses and amino acid transporters has also been reported. Several amino acid transporters have been identified as host factors affecting the viral life cycle and cellular response to infection. For instance, deletion of an amino acid transporter in the midgut membrane inhibits bombyx parvo-like virus (BmDNV-2) replication [69]; xCT is an inducible subunit of a membrane-bound amino acid transporter. KSHV-encoded microRNAs upregulate xCT expression to promote viral replication [70]; a Na^+^-dependent neutral-amino acid transporter mediates feline and baboon endogenous retroviruses infection [71]; ASCT2, a member of a glutamate transporter superfamily, is an auxiliary receptor for baboon endogenous retrovirus (BaEV) [72]. Here, we identified the amino acid transporter for the first time during FMDV infection and showed that SLC38A8 was responsible for FMDV-induced aspartate. SLC38A8 is involved in the transport of arginine, alanine, histidine, glutamine, and aspartate. However, FMDV infection promoted the level of aspartate, but not arginine, alanine, histidine, or glutamine in cells and pigs. The cellular microenvironment can affect the uptake of specific nutrients despite conserving key regulatory pathways [73]. Viral infection is a complicated process, which may affect cellular microenvironment or activate a series of signaling pathways to alter the affinity of SLC38A8 for amino acids, resulting in high levels of aspartate uptake by SLC38A8 during FMDV infection. The levels of aspartate are decreased in the SLC38A8^-/-^ cells compared to that in the WT cells, but the reduction is approximately 50%, suggesting that other mechanisms and/or transporters may regulate the levels of aspartate in cells during FMDV infection. However, aspartate plays important role during FMDV replication, and knockout of SLC38A8 significantly inhibited FMDV replication, suggesting that SLC38A8-mediated aspartate uptake is critical for FMDV replication. Further studies on the other manners of aspartate regulation could help clarify the complicated mechanisms of metabolism change during FMDV infection.

The expression of amino acid transporters may be regulated by multiple factors. Myc oncogenes regulate multiple aspects of tumor metabolism, inducing tumor cells to take up glucose and amino acids. MYC selectively activates SLC7A5 and SLC43A1 transcription through direct binding to E box elements in both genes, but it did not affect the expression of SLC38A8 [74]. The activation of mTORC1 can promote some amino acid transporters expression, and the mTORC1 pathway is associated with the expression of SLC38A8 [44,75]. Here, our data showed that inhibition of the mTORC1 signaling pathway decreased SLC38A8 expression in FMDV-infected cells, revealing the importance of the mTORC1 signaling pathway in regulating SLC38A8 expression during FMDV infection.

mTORC1 activation can be induced by amino acids, such as arginine, glutamine, and leucine, accompanied by the dynamic lysosomal localization of the mTOR and TSC complexes. Amino acids regulate mRNA translation via an mTOR-dependent pathway [76,77]. Here, we investigated the mechanism by which amino acids activated downstream signaling pathways to promote FMDV replication. Our data indicated that FMDV-induced upregulation of amino acids promoted the transfer of mTOR to the lysosome and increased interaction between mTOR and Rheb, resulting in the activation of Rheb/mTOR/p70S6K1/rps6 signaling axis to promote viral replication. Detailed analysis showed that Rheb protein was activated by PI3K/AKT/TSC2 pathways during FMDV infection. The PI3K/AKT pathway can be regulated by many viruses, including NDV [39], DENV [78], and rotaviruses [79]. The previous results showed FMDV entry via macropinocytosis is independent of PI3K [80]. In the present study, the regulatory relationship between FMDV and PI3K was further elucidated, revealing the importance of PI3K on FMDV-induced amino acid metabolism.

**Fig 11:**
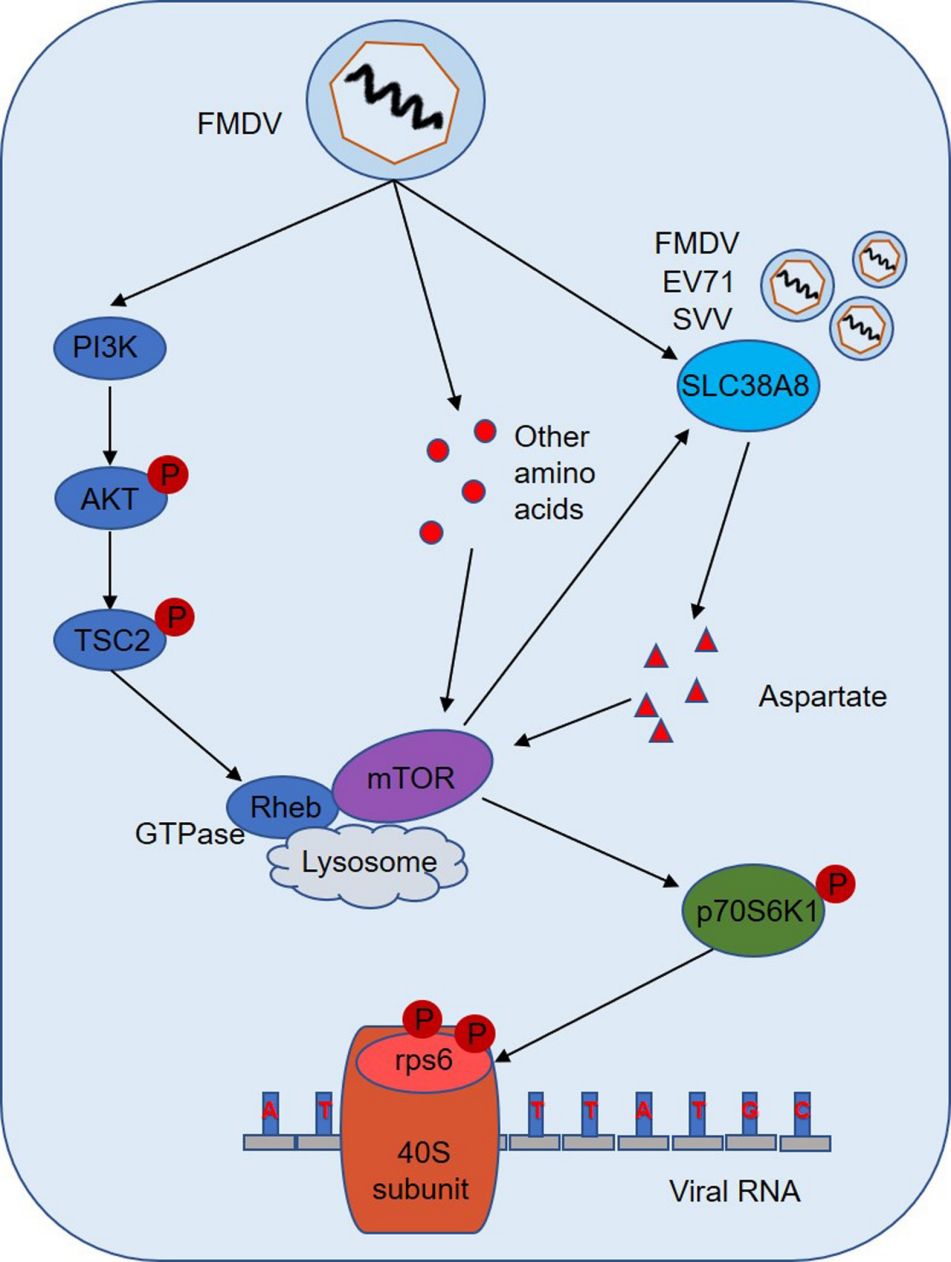
Schematic overview of the working model of how FMDV infection regulates host amino acid metabolism. FMDV infection significantly promoted the level of aspartate *in vitro and in vivo*. Cellular aspartate plays an important role in FMDV replication. Detailed analysis showed that FMDV enhanced aspartate by SLC38A8 transporter. EV71 and SVV infection also promoted aspartate by SLC38A8. On one hand, FMDV infection activated Rheb via PI3K/AKT/TSC2 pathways. On the other hand, the upregulation of amino acids enhanced the interaction between mTOR and Rheb to activate the mTOR/p70S6K1 signaling axis to promote viral replication. The mTORC1 signaling pathway was responsible for FMDV-induced SLC38A8 protein expression.

In conclusion, FMDV infection promoted the level of aspartate *in vitro and in vivo*. Cellular aspartate plays an important role in FMDV replication. Detailed analysis showed that FMDV enhanced aspartate by SLC38A8 transporter *in vitro and in vivo*. EV71 and SVV infection also promoted aspartate by SLC38A8. On the one hand, FMDV infection activated Rheb via PI3K/AKT/TSC2 pathways. On the other hand, the upregulation of amino acids enhanced the interaction between mTOR and Rheb to activate the mTOR/p70S6K1 signaling axis to promote viral replication. The mTORC1 signaling pathway was responsible for FMDV-induced SLC38A8 protein expression (Fig 11).

## Materials and methods

### Ethics statement

SLC38A8^-/-^ mice (C57BL/6) were purchased from Cyagen Biosciences, China and maintained in the specific pathogen-free (SPF) animal facility of Lanzhou Veterinary Research Institute with free access to food and water. Mouse experimental work was performed using 3-day-old mice, and was age- and sex-matched in each experiment. All animals were handled in strict accordance with good animal practice according to the Animal Ethics Procedures and Guidelines of the People’s Republic of China, and the study was approved by the Animal Ethics Committee of Lanzhou Veterinary Research Institute of the Chinese Academy of Agricultural Sciences (Licence no. SYXK (GAN) 2010–003).

### Cells and viruses

PK-15, BHK-21, RD, and HEK-293T cells were cultured in Dulbecco’s modified Eagle medium (DMEM, Gibco) supplemented with 10% heat-inactivated fetal bovine serum (Gibco) and maintained at 37°C (5% CO_2_). FMDV type O strain O/BY/CHA/2010 was used for the viral challenge. The EV71 and SVV strains prepared in our laboratory were used for the viral challenge [4]. Viral infection experiments were carried out as described previously [81].

### Titration of FMDV and EV71 from mice

Viruses from mice carcasses without the head, tail, limbs, and viscera were isolated, as described previously [82]. Briefly, to quantify viral particles, part of the carcass was weighed and homogenized using disposable tissue grinders (VWR, Radnor, PA) in DMEM supplemented with 1% penicillin-streptomycin-neomycin antibiotic mixture, 2.5 μg/mL fungizone, and 1% L-Glutamax (ThermoFisher, Waltham, MA). Titration of FMDV and EV71 was performed using BHK-21 and RD cells, respectively, as described previously [82,83].

### Preparation of aspartate and glutamate medium

According to the manufacturer’s instructions, aspartate (SA8560) and glutamate (YZ-100023), purchased from Solarbio, Beijing, China, were dissolved into DMEM medium to a final concentration of 100 mg/L. To assess the impact of aspartate and glutamate on viral replication, the cells were pre-treated with different doses of aspartate or glutamate for 2 h prior to viral infection.

### Antibodies

The commercial antibodies used in this study included an anti**-**β**-**actin monoclonal antibody (Thermo Scientific, Waltham, MA, USA), anti-mTOR monoclonal antibody (Cell Signaling Technology, Beverly, MA, USA), anti-LAMP2 monoclonal antibody (Cell Signaling Technology), anti-Rheb monoclonal antibody (Cell Signaling Technology), anti-AKT polyclonal antibody (ABclonal Technology Co.,Ltd, Wuhan, China), anti-p-AKT polyclonal antibody (ABclonal), anti-rps6 polyclonal antibody (ABclonal), anti-p-rps6 polyclonal antibody (ABclonal), anti-p70S6K1 polyclonal antibody (ABclonal), anti-p-p70S6K1 polyclonal antibody (ABclonal), anti-TSC2 polyclonal antibody (ABclonal), anti-p-TSC2 polyclonal antibody (ABclonal), anti-EV71 3C polyclonal antibody (ABclonal), Anti-FMDV and SVV VP1 polyclonal antibody was prepared in our laboratory. Anti-SLC38A8 polyclonal antibody was produced in rabbits by immunization with porcine SLC38A8 protein.

### Sample collection

PK-15 cells (10^7^ cells) were mock-infected or infected with FMDV upon the same incubation time in the same culture medium and were collected using cell scrapers. Then, the cells were centrifuged at 12,00 rpm for 5 min at 4°C. The precipitated cells were resuspended with ice-cold PBS and were rapidly quenched using a fivefold volume of ice-cold quenching solution (60% aqueous methanol, 0.85% (w/v) ammonium bicarbonate, pH 7.4). Subsequently, the samples were centrifuged to remove the supernatant, and the pellet was quickly frozen using liquid nitrogen. To identify the FMDV infection, the cell culture supernatants were collected for virus titration, and the cells were lysed for Western blotting analysis. The same number of serum and oropharyngeal tonsils samples from FMDV-infected pigs were used to detect metabolites using metabolomics.

### Metabolite extraction and data acquisition through LC-MS analysis

Metabolite extraction and data acquisition through LC-MS analysis were performed as described in our previous study [22]. Briefly, 50 μL pre-cooled methanol/acetonitrile/water solution (2:2:1, v/v) was added to the samples and mixed by vortexing for 20 s. Then, the samples were treated with ultrasound and centrifuged at 14,000 rpm for 20 min. The supernatant was vacuum-dried. During mass spectrometry, 100 μl acetonitrile-water solution (acetonitrile:water = 1:1, v/v) was added to the samplescentrifuged at 14000g for 15 min. The supernatant was separated by Agilent 1290 Infinity LC ultra-high-pressure liquid chromatography (UHPLC), with a column temperature of 25°C, a flow rate of 0.5 mL/min, and a sample volume of 2 μL. The samples were placed in a 4°C autosampler and analyzed randomly. The electrospray ionization (ESI) positive and negative ion modes were used for mass spectrometer (MS) detection. The spectrogram was collected using AB Triple TOF6600 mass spectrometer (AB SCIEX, Danaher, Washington, DC, USA).

### Amino acid metabolism analysis

The tonsils samples from FMDV-infected pigs were used for amino acid metabolism analysis (also called targeted analysis). The ESI positive ion modes were selected. The spectrogram was detected by a 5500 QTRAP mass spectrometer (AB SCIEX). The parameters of the mass spectrometer are: source temperature: 500°C, ion Source Gas1 (Gas1):40, Ion Source Gas2(Gas2): 40, Curtain gas (CUR): 30, ionSapary Voltage Floating (ISVF) 5500 V. The ion peaks of metabolites were compared with the standard substances.

### Metabolites analysis using kits

The activity of AST in the PK-15 cells and serum was detected using an aspartate aminotransferase (AST/GOT) activity detection kit (MAK055, Sigma-Aldrich, St. Louis, MO, USA). The level of aspartate was detected using an aspartate detection kit (MAK095, Sigma-Aldrich).

### ^13^C-labelled aspartate assay

The labelled aspartate (489999, Sigma-Aldrich: aspartate-4-^13^C with a ^13^C fraction of 99%) experiments were performed as described previously [84]. Briefly, cells incubated with ^13^C-aspartate were mock-infected or infected with FMDV, EV71, or SVV. At the indicated time points, the samples were collected. After that, 500 μL of cold extraction buffer (methanol:acetonitrile:water = 2:2:1, v/v/v) was added to each sample. Samples were sonicated for 2 minutes and centrifuged at 14,000 g for 5 min at 4°C. Supernatants were lyophilized and reconstituted in 50 μL of methanol-water (1:1, v/v). Then, the level of ^13^C-aspartate was detected using HPLC and was quantified by comparison with the standard substance.

### Indirect immunofluorescence assay

Cells cultured on Nunc glass-bottom dishes (Thermo Fisher Scientific) were mock-infected or infected with FMDV. The cells were fixed with formaldehyde overnight at 4°C and were incubated with 5% normal bovine serum and 1% Triton-X and appropriate primary antibodies (1:200) overnight at 4°C. The fluorochrome-conjugated secondary antibodies (1:500) were used to react with the primary antibodies in the dark for 2 h at room temperature. After washing with PBS three times, the cells were stained with 4’,6-diamidino-2-phenylindole (DAPI) for 10 minutes to show the nuclei. The fluorescence was visualized using a Nikon Eclipse 80i fluorescence microscope. The images were captured using NIS Elements F 2.30 software.

### Coimmunoprecipitation and Western blotting analysis

PK-15 cells cultured in 10-cm dishes were mock-infected or infected with FMDV. The collected cells were lysed and immunoprecipitated as described previously [85]. The target proteins were analyzed by 10% sodium dodecyl sulfate-polyacrylamide gel electrophoresis (SDS-PAGE) and transferred to nitrocellulose membranes (Millipore, Bedford, MA, USA), which were blocked with 5% skim milk in TBST and treated with appropriate primary (1:1000) overnight at 4°C and secondary antibodies (1:10000) at room temperature for 2 h. The antibody-antigen complexes were visualized using enhanced chemiluminescence detection reagents (Share-bio Biotechnology, Shanghai, China). The abundance of proteins was determined by densitometric analysis using ImageJ Software.

### Knockdown of Rheb using siRNA

The small interfering RNA (siRNA) used in this study were designed and synthesized by Gene Pharma (Shanghai, China). Knockdown of endogenous Rheb in PK-15 cells was performed by transfection of Rheb siRNA. Negative control (NC) siRNA was used as a negative control. According to the manufacturer’s protocol, the siRNA was transfected with Lipofectamine 2000 (Invitrogen). The porcine Rheb siRNA sequence was F: GUCAUCCAUGGCAAAUUGUTT, R: ACAAUUUGCCAUGGAUGACTT.

### Establishment of knockout cell lines using CRISPR/Cas9 system

The SLC38A8 knockout cell line was established as described previously [86]. The small guide RNAs (sgRNA) target SLC38A8 were designed using the online CRISPR design tool (http://crispr.mit.edu/). The sgRNA was inserted into the pLentiCRISPR plasmid with the puromycin selection gene. The constructs were transfected to PK-15 cells using Lipofectamine 3000 (Thermo Fisher Scientific). Then, the cells were incubated with puromycin (2 μg/mL) to obtain stable knockout cells. After confirmation of the activity of the designed sgRNA using the T7 Endonuclease I (NEB), the KO cell lines were confirmed by Western blotting. Control cells were transfected with the empty vector. The porcine SLC38A8 sgRNA sequences are: TCATGCGGAGAAGCCAGTGT. The human SLC38A8 sgRNA sequences are: ATCCTCATGAAGTCCGCGCT.

### RNA extraction and quantitative PCR

Total RNAs in the PK-15 cells were extracted using TRIzol reagent (Invitrogen). The Moloney murine leukemia virus reverses transcriptase (Promega, Madison, WI, USA), random hexamer primers (TaKaRa, Shiga, Japan), and total RNAs were used to synthesize cDNAs. The cDNAs, SYBR Premix Ex Taq reagents (TaKaRa, Dalian, China), and Mx3005P qPCR System (Agilent Technologies, Palo Alto, CA, USA) was used to detect host mRNA levels. The *GAPDH* gene was used as an internal control. The relative expression of mRNA was calculated based on the comparative cycle threshold (CT) (2^−ΔΔCT^) method [87]. The qPCR primer sequences are listed in Table 1.

**Table 1:** The qPCR primers used in this study.

Primers	Sequences(5’-3’)	Target gene
SLC1A1-F	ATCCACTCCATTGTTATTCTGC	porcine SLC1A1 gene
SLC1A1-R	CTCTTGTCCACCTGGTTCTTCT	
SLC1A2-F	GGGGCAGTATGTGGAGGATTT	porcine SLC1A2 gene
SLC1A2-R	TGCTTCTTGAGTTTGGGGTTC	
SLC1A3-F	GCCCCTCCTCTACTTCTTGGT	porcine SLC1A3 gene
SLC1A3-R	TGCTGATGGTGATAATCTGTC	
SLC1A6-F	GTCATTGTGCTCACCTCGGTT	porcine SLC1A6 gene
SLC1A6-R	CCTGGACGCCCCCTTCTCTTG	
SLC1A7-F	CACTTCCGTGGGTCTTCCTAC	porcine SLC1A7 gene
SLC1A7-R	ACACTCTTCACACAGCCGTTC	
SLC25A12-F	AAGAGTGGAAATGGAGAGGTG	porcine SLC25A12 gene
SLC25A12-R	CGATTATGCCCAAAATGTAGC	
SLC25A13-F	CAAGCGAAGATGGGCAGATTA	porcine SLC25A13 gene
SLC25A13-R	ACTGGGGAGAGGACCGAAACA	
SLC38A7-F	AGTGGTGTGGGCTGTGTGTG	porcine SLC38A7 gene
SLC38A7-F	GCTGATGGTGAACTTGCGGT	
SLC38A8-F	GACGCTGCTCTCCCTGCTGCT	porcine SLC38A8 gene
SLC38A8-R	TTCGTGACACTGAAATCCAAA	
GAPDH-F	ACATGGCCTCCAAGGAGTAAGA	porcine GAPDH gene
GAPDH-R	GATCGAGTTGGGGCTGTGACT	

### Cell counting assay

The cell growth numbers of WT or SLC38A8^-/-^ cells and the impact of different doses of aspartate and glutamate on cell growth numbers were assessed using an Automated Cell Counter (RuiYu Biotech Co.Ltd, Shanghai, China), according to the manufacturer’s instructions.

### CCK-8 assay

CCK-8 assay was performed using CCK-8 Cytotoxicity Assay Kit (CA1210, Solarbio) to detect cell viability. Approximately 10^4^ cells were seeded in 96-well plates with 100 μl medium each well. After 12 h incubation, different doses of inhibitors (MedChemExpress, Monmouth Junction, NJ), including T23 (HY-15644), metformin (HY-B0627), lidocaine (HY-B0185), LY294002 (HY-10108), rapamycin (HY-10219), and NR1 (HY-124798) were added respectively. Each well was incubated with 10 μL of CCK-8 solution for 4 h away from light before measuring the absorbance at 450 nm using a spectrofluorometer (Thermo Scientific).

### Statistical analysis

The raw data for peak alignment, calibration, and retention time peak area extraction were analyzed by XCMS software (https://xcmsonline.scripps.edu/index.php). The accurate mass matching (<25 ppm) was used to identify metabolite structure. Ion peaks with missing values >50% in the data group were ignored. Multidimensional statistical analysis, including principal component analysis, supervised partial least squares discriminant analysis (PLS-DA), and orthogonal partial least squares discriminant analysis (OPLS-DA), were performed using SIMCA-P 14.1 software package (Sartorius Stedim Data Analytics AB, Umea, Sweden). Variable importance for the projection (VIP) was obtained from the OPLS-DA model. OPLS-DA VIP>1 and *p*-value <0.05 were used as the screening criteria for significant differential metabolites. The cluster maps were generated using the R program and the metabolic pathways were found using the KEGG enrichment analysis. Single-dimensional statistical analysis was performed using SPSS Statistics for Windows, Version 17.0 (SPSS Inc., Chicago, IL, USA). The student’s *t*-test and and variation multiple analyses were used for a comparison of the raw data. A *p*-value <0.05 was considered statistically significant (*); a *p*-value <0.01 was considered statistically highly significant (**).

## Supporting information

S1 Fig Significant changes in metabolites during FMDV infection. Heatmap of hierarchical clustering analysis of differential metabolites. Each column represents one sample, and each row represents one differential metabolite. Red, upregulation; blue, downregulation; F, FMDV. n = 6 samples at each time point.
(TIF)

S2 Fig Bubble plots of the metabolic pathway analysis in FMDV-infected cells. Each bubble in the bubble diagram represents a metabolic pathway. The larger the bubble, the more metabolites number is. The X-axis represents a pathway impact value in the topology analysis, and the size is positively correlates with the influence factor. The Y-axis represents the p-value of the metabolic pathway in the enrichment analysis. The darker the color, the smaller the P-value, indicating the more significance for the enrichment degree. The top 20 metabolic pathways with the highest significance according to the P-value were shown.
(TIF)

S3 Fig The amount of amino acids in tonsils in FMDV-infected pigs. The tonsils in the FMDV-infected pigs were collected at 0, 3, and 5 dpi. The amount of amino acids in the tonsils was quantified by targeted analysis. Values are chromatographic peak area. n = 5 samples at each time point.
(TIF)

S4 Fig All the indicated doses of inhibitors did not induce significant cell death. PK-15 and HEK-293T cells were seeded in six-well plates, and the monolayer cells were maintained in the presence or absence of the T23 and lidocaine for 24 h, and metformin, LY294002, rapamycin, or NR1 for 16 h, respectively. The cytotoxicity of the indicated doses of inhibitors was measured by CCK-8 assay. n = 8. Results represent two independent experiments.
(TIF)

S5 Fig The impact of aspartate, glutamate, and SLC38A8 on the number of cells. (A and B) PK-15 or HEK-293T cells were seeded in six-well plates, and the monolayer cells were maintained in the presence of aspartate or glutamate (0, 25, and 50 mg/L) for 0, 12, 24, and 48 h. The cell growth numbers were detected using an Automated Cell Counter. n = 6. Results represent two independent experiments. (C) WT and SLC38A8^-/-^ PK-15 or HEK-293T cells were cultured for 0, 12, 24, and 48 h. The cell growth numbers were detected using an Automated Cell Counter. n = 6. Results represent two independent experiments.
(TIF)

S6 Fig The level of aspartate in mice. The two-day-old WT mice (n = 3 mice per group) were subcutaneously mock-injected or injected with 100 mg of aspartate for 24 h. The levels of aspartate in mice carcasses without the head, tail, limbs, and viscera were detected using an aspartate detection Kit.
(TIF)

S7 Fig The impact of FMDV infection on the expression of amino acid transporter. PK-15 cells were infected with FMDV (MOI 0.5) for 0, 4, 8, and 12 h. The cells were collected to extract mRNA. The mRNA levels of SLC1A1, SLC1A2, SLC1A3, SLC1A6, SLC1A7, SLC25A12, SLC25A13, and SLC38A7 were determined by qPCR. n = 3. Results represent two independent experiments.
(TIF)

S8 Fig The abundance of SLC38A8 protein in Fig 6 and Fig 8. The abundance of SLC38A8 protein in Fig 6 and Fig 8 were quantified by densitometric analysis using ImageJ Software. n = 3. Results represent three independent experiments. A, B, and C represent the quantitative result of the B, C, and E of Fig 6, respectively. D represents the quantitative result of A of Fig 8.
(TIF)

S9 Fig The abundance of mTOR, Rheb, or LAMP2 protein in Fig 9. The abundance of the proteins in Fig 9 was quantified by densitometric analysis using ImageJ Software. n = 3. Results represent three independent experiments. A and B represent the quantitative result of C and D of Fig 9, respectively.
(TIF)

S10 Fig The abundance of the proteins in Fig 10. The abundance of rps6, p70S6K1, AKT, TSC2, Rheb, and SLC38A8 protein in Fig 10 was quantified by densitometric analysis using ImageJ Software. n = 3. Results represent three independent experiments. A, B, C, D, E, and F represent the quantitative result of A, B, C, D, E, and G of Fig 10, respectively.
(TIF)

S11 Fig The amino acid composition of viral proteins of FMDV, EV71, and SVV. The amino acid composition of viral proteins of FMDV, EV71, and SVV was analyzed (n = 3), according to the amino acid sequence at NCBI (https://www.ncbi.nlm.nih.gov/). FMDV: GenBank No. JQ900581.1, KY234502.1, and MN389541.1. EV71: GenBank No. EU812515.1, AF302996.1, and HQ611148.1. SVV: GenBank No. MT457474.1, MK170054.1, and MK170056.1. A: Alanine, L: Leucine, V: Valine, T: Threonine, G: Glycine, D: Aspartate, K: Lysine, P: Proline, E: Glutamate, S: Serine, F: Phenylalanine, I: Isoleucine, R: Arginine, N: Asparagine, Y: Tyrosine, Q: Glutamine, H: Histidine, M: Methionine, C: Cystine, W: Tryptophan.
(TIF)

S1 Data Excel spreadsheet containing, in separate sheets, the underlying numerical data for Figure panels 1B, 5A-E, 6A, 6D-G, 7B-F, 8B-D, 10F, 10G, s3, s4, s5, s6, s7, s8, s9, s10, and s11. (XLSX)

S2 Data This file contains two independent experiments for Figs 6B, 6C, 6E, 8A, 9C, 9D, 10A, 10B, 10C, 10D, 10E, and 10G. (PDF)

S3 Data This file contains unadjusted Western blot images for Figs 1A, 6B, 6C, 6E, 7A, 8A, 9C, 9D, 10A, 10B, 10C, 10D, 10E, and 10G. (PDF)

S4 Data This file contains unadjusted Western blot images for two independent experiments. (PDF)
