# Supplementary figures and images for: Picornavirus infection enhances aspartate by the SLC38A8 transporter to promote viral replication
Source: PLoS Pathog. 2023 Feb 3;19(2):e1011126. doi: 10.1371/journal.ppat.1011126 (PMC9931120; doi:10.1371/journal.ppat.1011126)

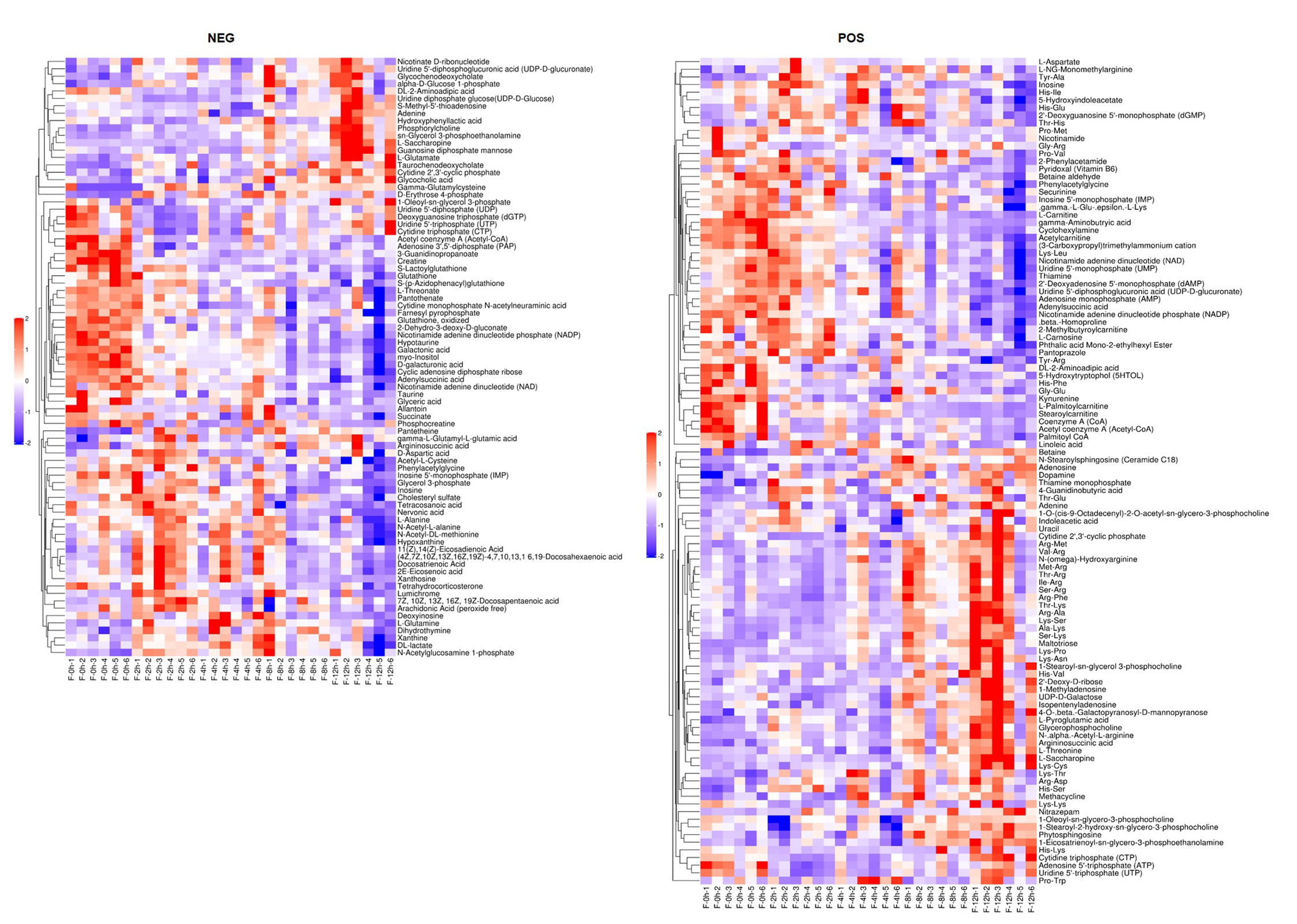

Supplement: S1 Fig — Heatmap of hierarchical clustering analysis of differential metabolites. Each column represents one sample, and each row represents one differential metabolite. Red, upregulation; blue, downregulation; F, FMDV. n = 6 samples at each time point. (TIF) [file ppat.1011126.s001.tif]

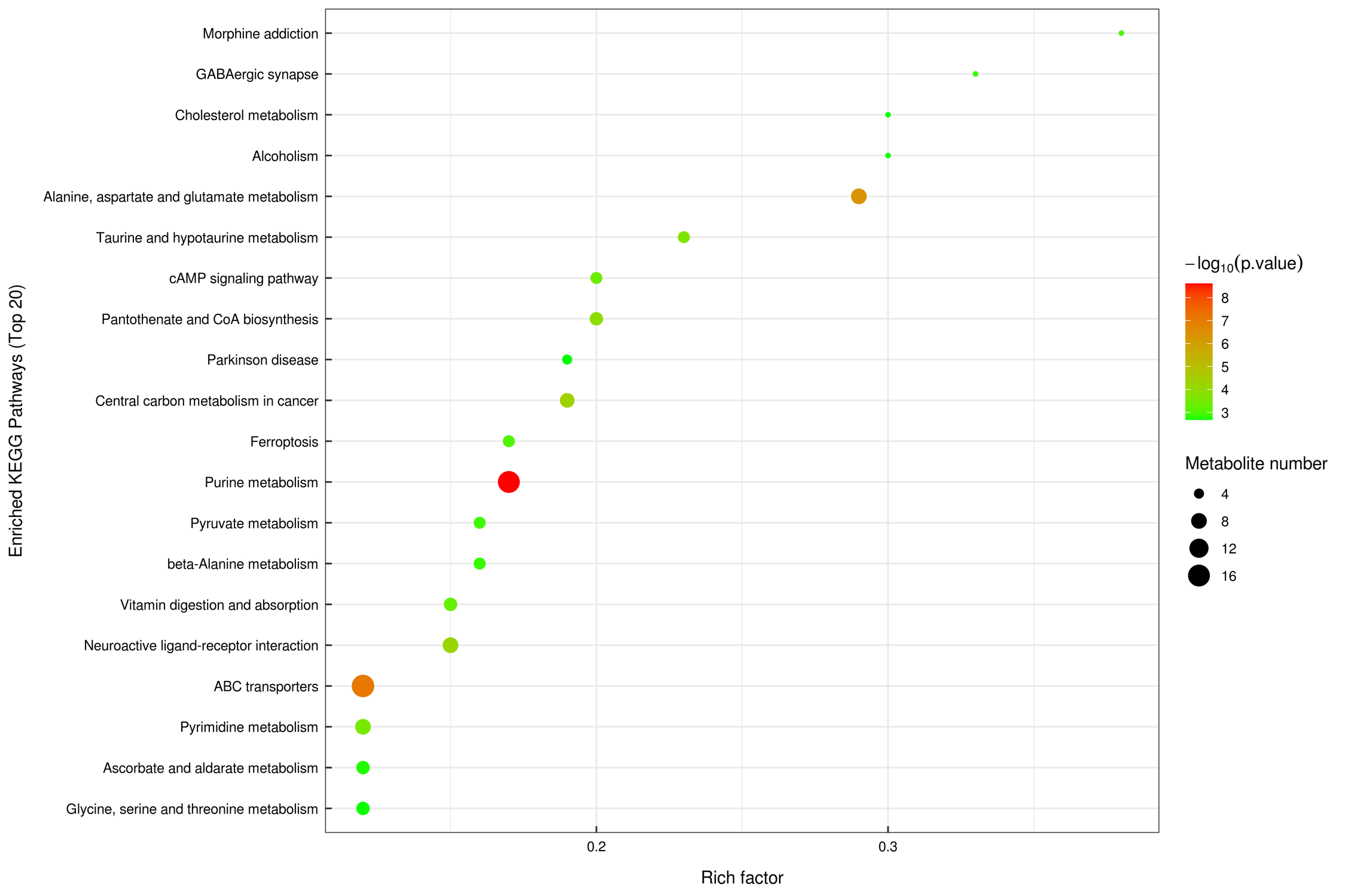

Supplement: S2 Fig — Each bubble in the bubble diagram represents a metabolic pathway. The larger the bubble, the more metabolites number is. The X-axis represents a pathway impact value in the topology analysis, and the size is positively correlates with the influence factor. The Y-axis represents the p-value of the metabolic pathway in the enrichment analysis. The darker the color, the smaller the P-value, indicating the more significance for the enrichment degree. The top 20 metabolic pathways with the highest significance according to the P-value were shown. (TIF) [file ppat.1011126.s002.tif]

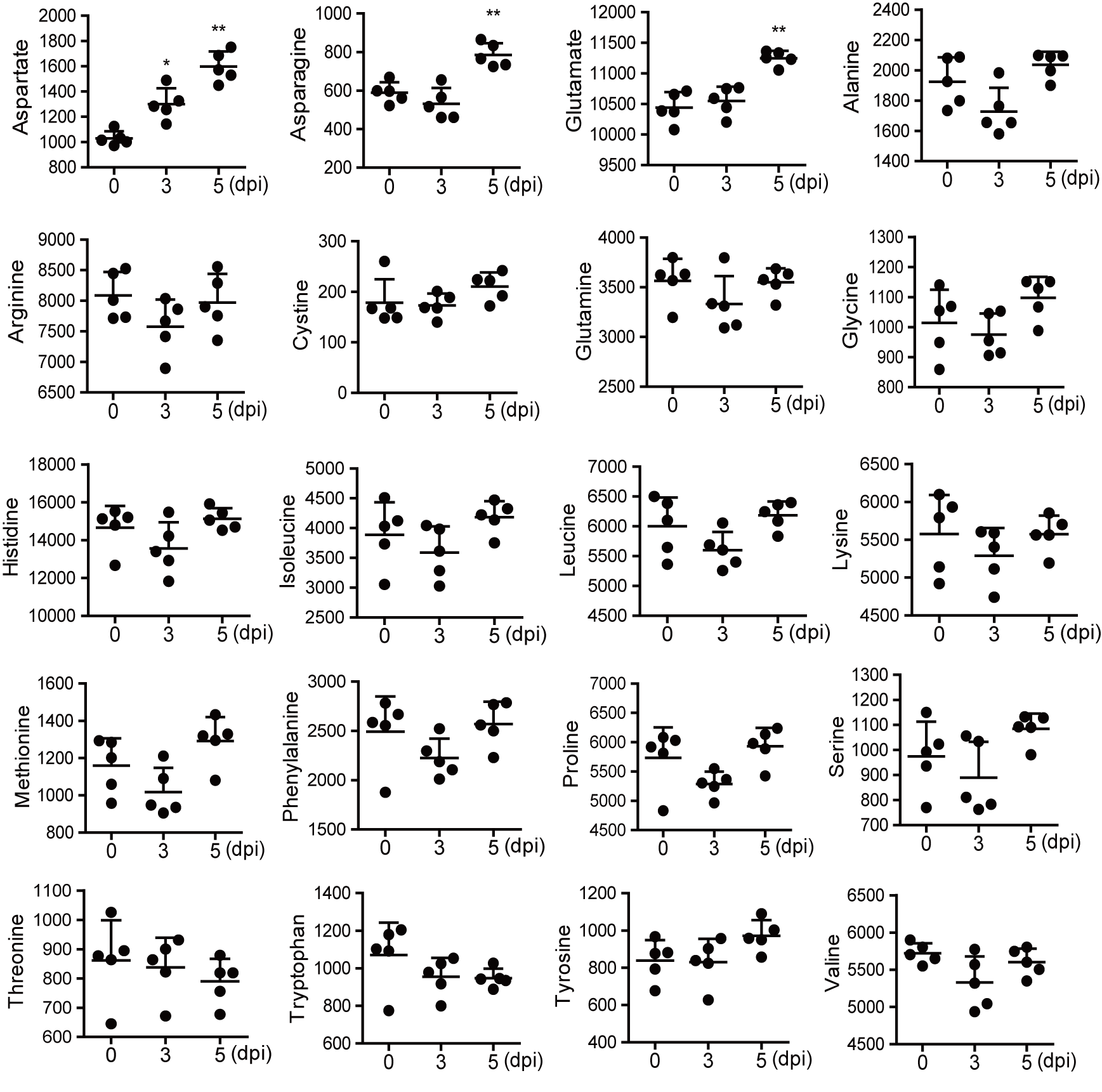

Supplement: S3 Fig — The tonsils in the FMDV-infected pigs were collected at 0, 3, and 5 dpi. The amount of amino acids in the tonsils was quantified by targeted analysis. Values are chromatographic peak area. n = 5 samples at each time point. (TIF) [file ppat.1011126.s003.tif]

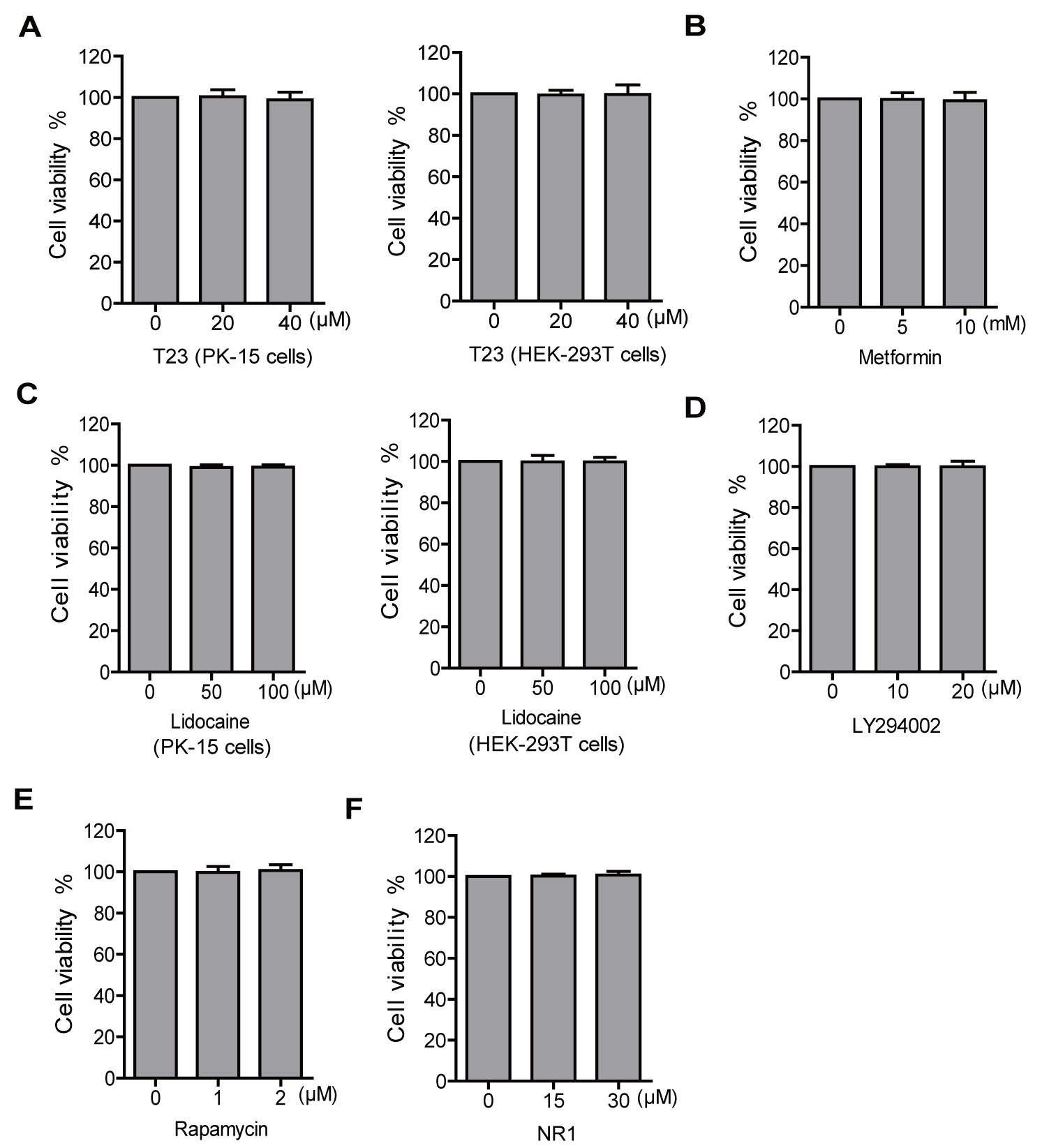

Supplement: S4 Fig — PK-15 and HEK-293T cells were seeded in six-well plates, and the monolayer cells were maintained in the presence or absence of the T23 and lidocaine for 24 h, and metformin, LY294002, rapamycin, or NR1 for 16 h, respectively. The cytotoxicity of the indicated doses of inhibitors was measured by CCK-8 assay. n = 8. Results represent two independent experiments. (TIF) [file ppat.1011126.s004.tif]

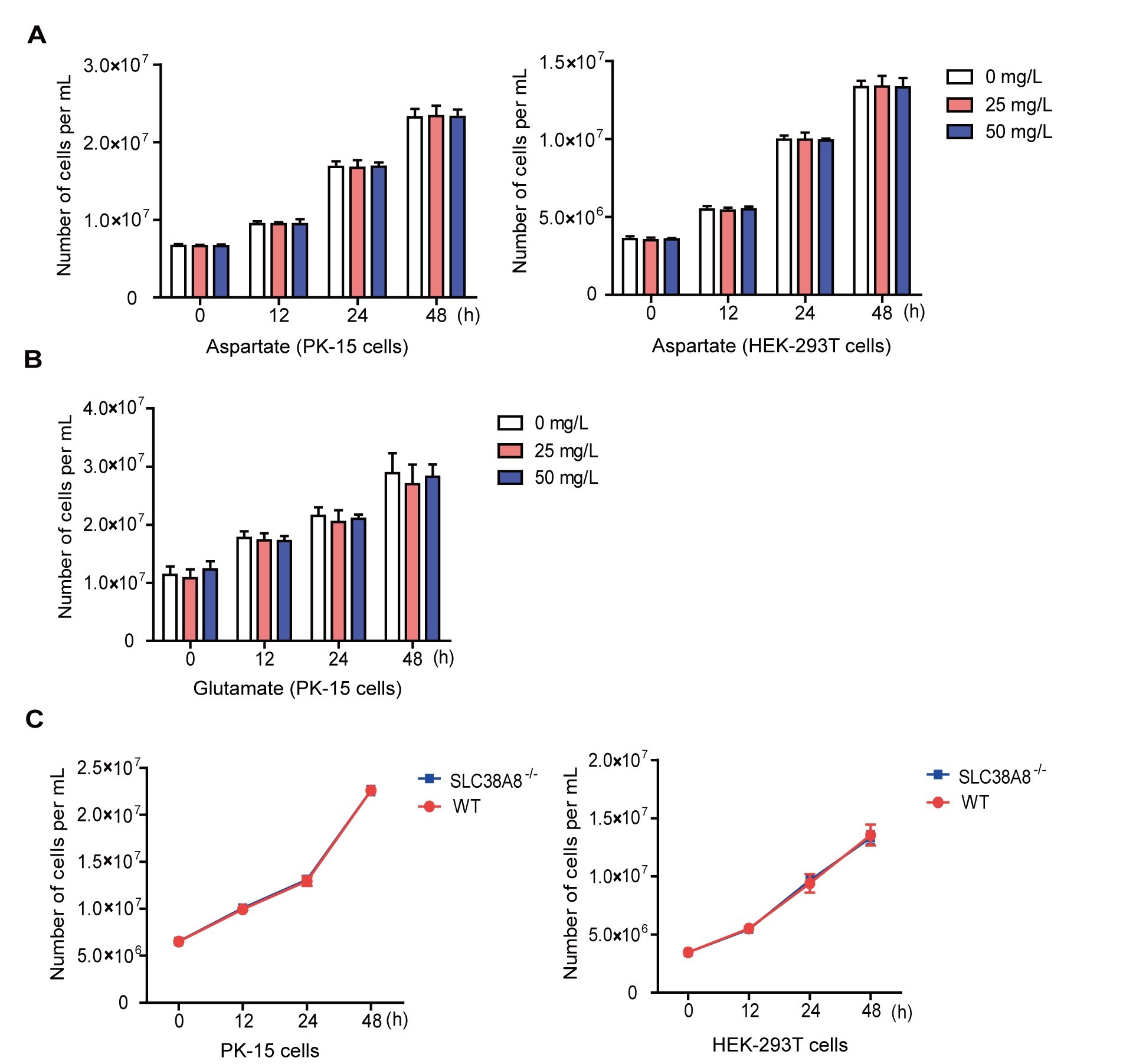

Supplement: S5 Fig — (A and B) PK-15 or HEK-293T cells were seeded in six-well plates, and the monolayer cells were maintained in the presence of aspartate or glutamate (0, 25, and 50 mg/L) for 0, 12, 24, and 48 h. The cell growth numbers were detected using an Automated Cell Counter. n = 6. Results represent two independent experiments. (C) WT and SLC38A8-/- PK-15 or HEK-293T cells were cultured for 0, 12, 24, and 48 h. The cell growth numbers were detected using an Automated Cell Counter. n = 6. Results represent two independent experiments. (TIF) [file ppat.1011126.s005.tif]

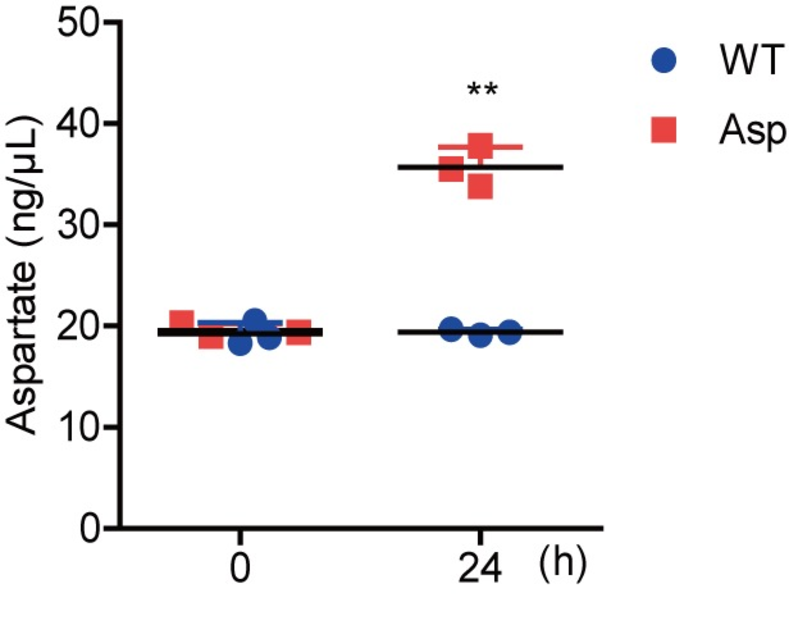

Supplement: S6 Fig — The two-day-old WT mice (n = 3 mice per group) were subcutaneously mock-injected or injected with 100 mg of aspartate for 24 h. The levels of aspartate in mice carcasses without the head, tail, limbs, and viscera were detected using an aspartate detection Kit. (TIF) [file ppat.1011126.s006.tif]

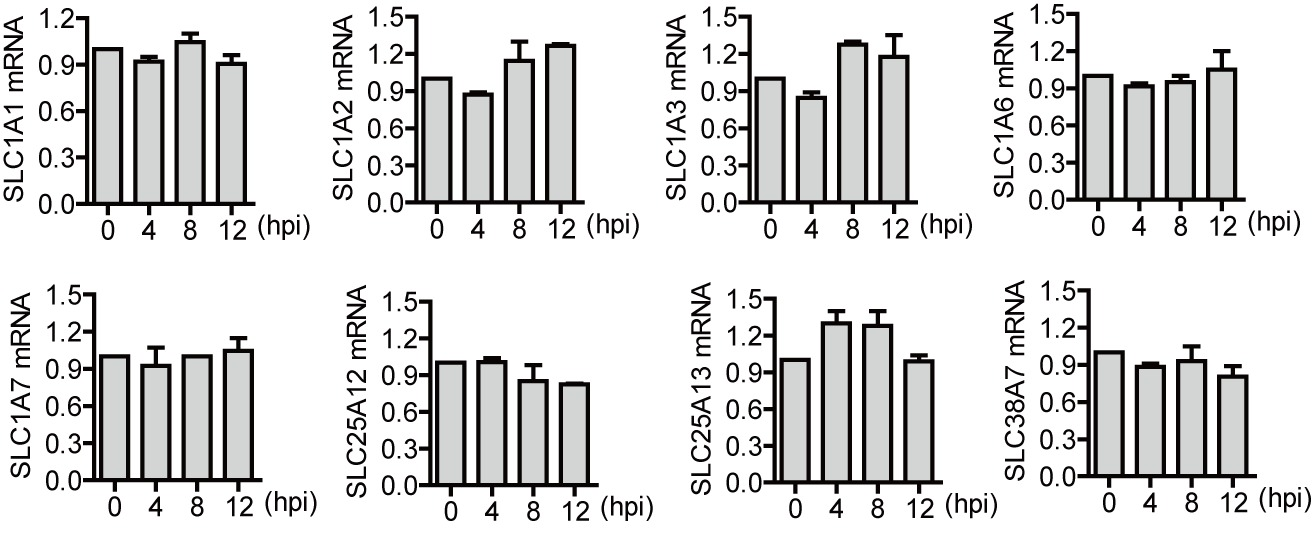

Supplement: S7 Fig — PK-15 cells were infected with FMDV (MOI 0.5) for 0, 4, 8, and 12 h. The cells were collected to extract mRNA. The mRNA levels of SLC1A1, SLC1A2, SLC1A3, SLC1A6, SLC1A7, SLC25A12, SLC25A13, and SLC38A7 were determined by qPCR. n = 3. Results represent two independent experiments. (TIF) [file ppat.1011126.s007.tif]

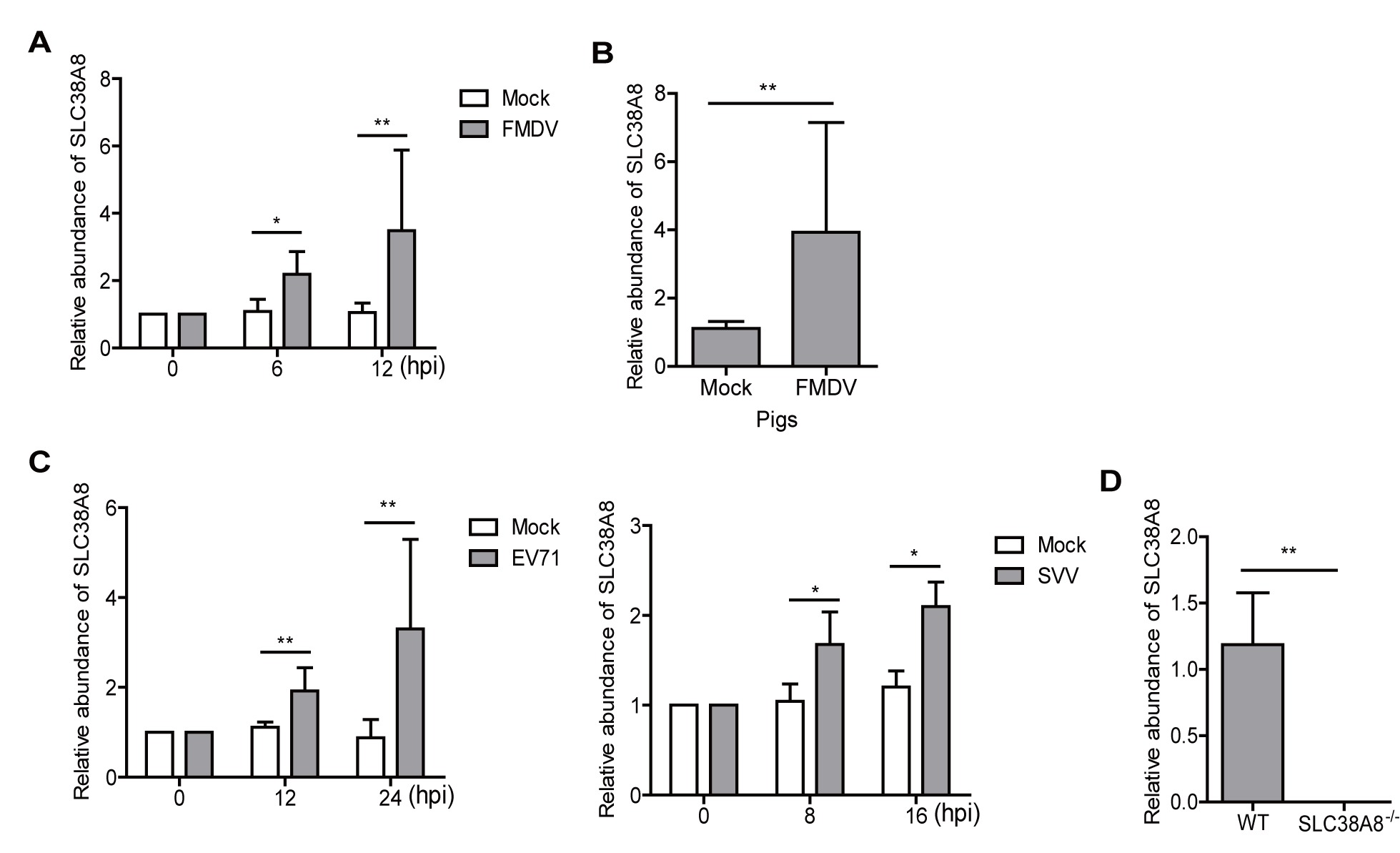

Supplement: S8 Fig — The abundance of SLC38A8 protein in Fig 6 and Fig 8 were quantified by densitometric analysis using ImageJ Software. n = 3. Results represent three independent experiments. A, B, and C represent the quantitative result of the B, C, and E of Fig 6, respectively. D represents the quantitative result of A of Fig 8. (TIF) [file ppat.1011126.s008.tif]

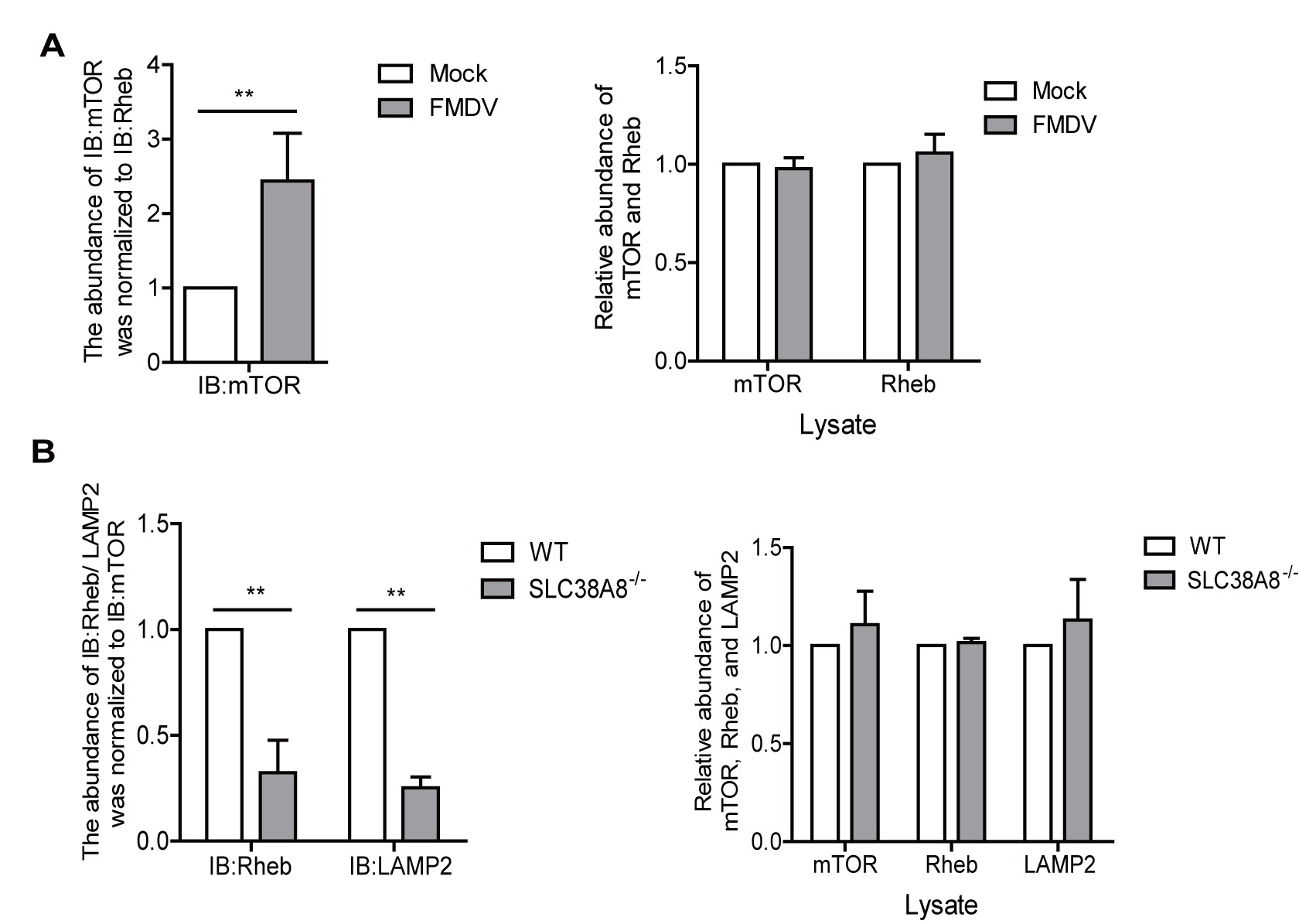

Supplement: S9 Fig — The abundance of the proteins in Fig 9 was quantified by densitometric analysis using ImageJ Software. n = 3. Results represent three independent experiments. A and B represent the quantitative result of C and D of Fig 9, respectively. (TIF) [file ppat.1011126.s009.tif]

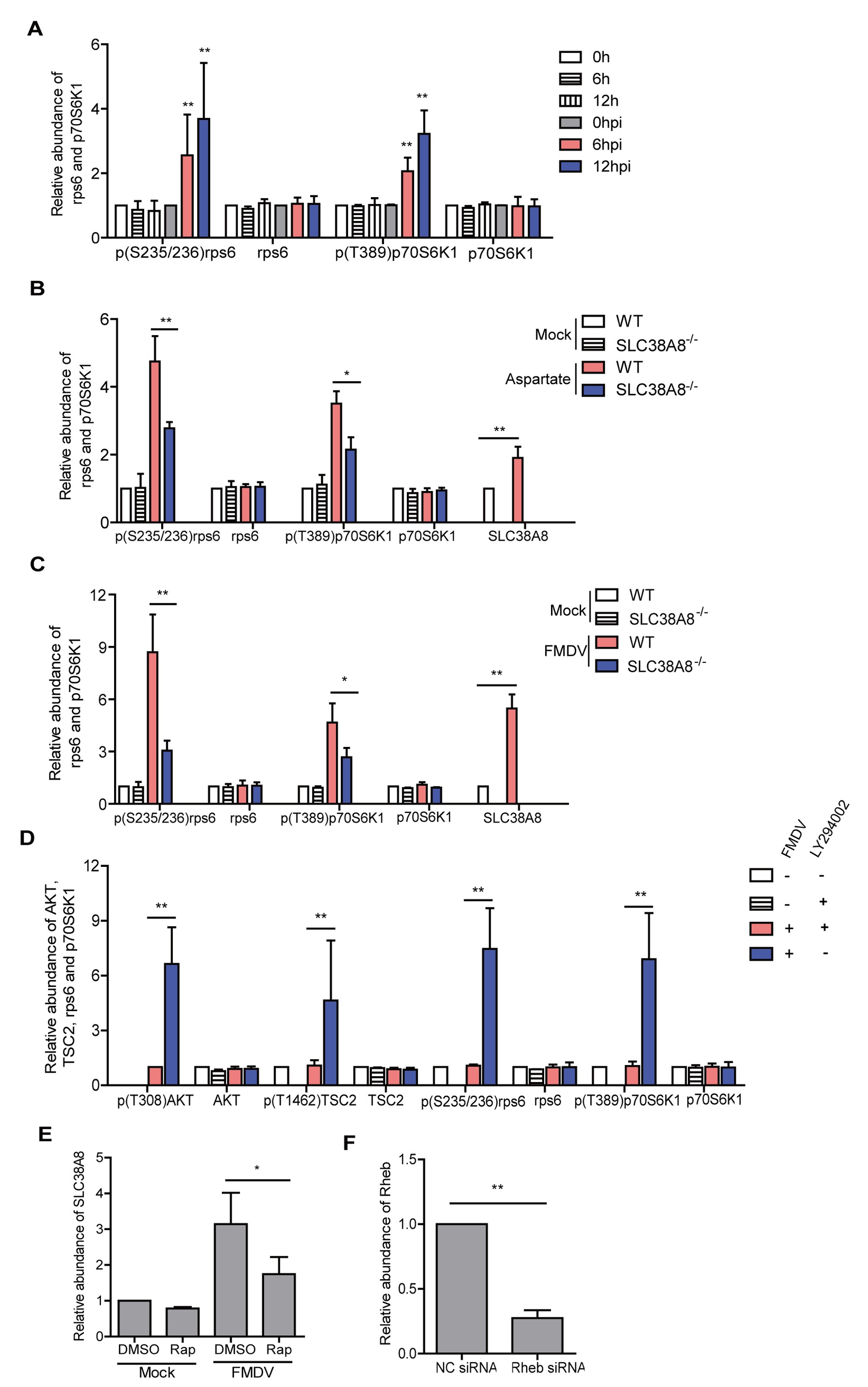

Supplement: S10 Fig — The abundance of rps6, p70S6K1, AKT, TSC2, Rheb, and SLC38A8 protein in Fig 10 was quantified by densitometric analysis using ImageJ Software. n = 3. Results represent three independent experiments. A, B, C, D, E, and F represent the quantitative result of A, B, C, D, E, and G of Fig 10, respectively. (TIF) [file ppat.1011126.s010.tif]

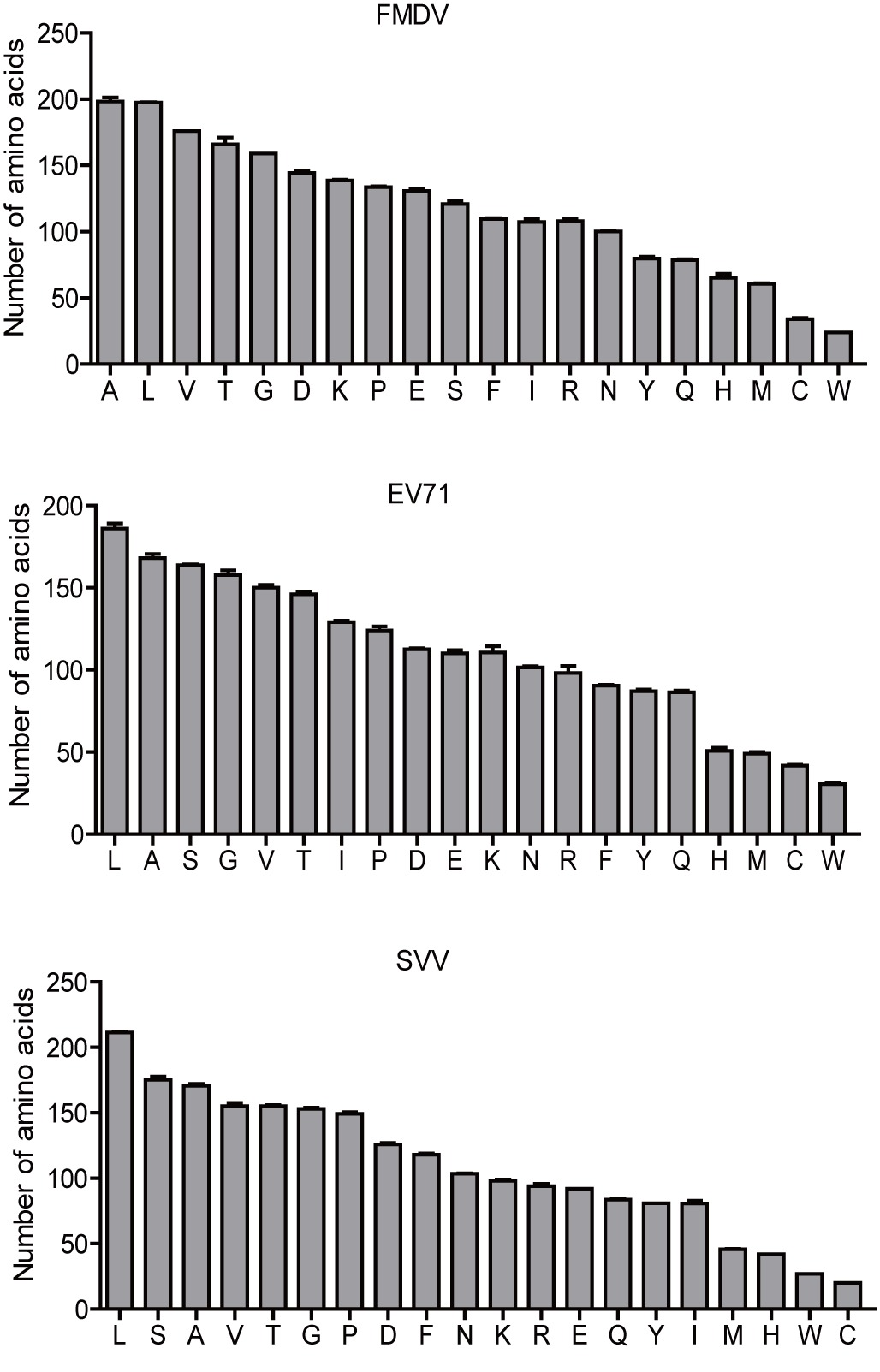

Supplement: S11 Fig — The amino acid composition of viral proteins of FMDV, EV71, and SVV was analyzed (n = 3), according to the amino acid sequence at NCBI (https://www.ncbi.nlm.nih.gov/). FMDV: GenBank No. JQ900581.1, KY234502.1, and MN389541.1. EV71: GenBank No. EU812515.1, AF302996.1, and HQ611148.1. SVV: GenBank No. MT457474.1, MK170054.1, and MK170056.1. A: Alanine, L: Leucine, V: Valine, T: Threonine, G: Glycine, D: Aspartate, K: Lysine, P: Proline, E: Glutamate, S: Serine, F: Phenylalanine, I: Isoleucine, R: Arginine, N: Asparagine, Y: Tyrosine, Q: Glutamine, H: Histidine, M: Methionine, C: Cystine, W: Tryptophan. (TIF) [file ppat.1011126.s011.tif]
