# Supplementary material for: Picornavirus infection enhances aspartate by the SLC38A8 transporter to promote viral replication
Source: PLoS Pathog. 2023 Feb 3;19(2):e1011126. doi: 10.1371/journal.ppat.1011126 (PMC9931120; doi:10.1371/journal.ppat.1011126)

Fig 6B

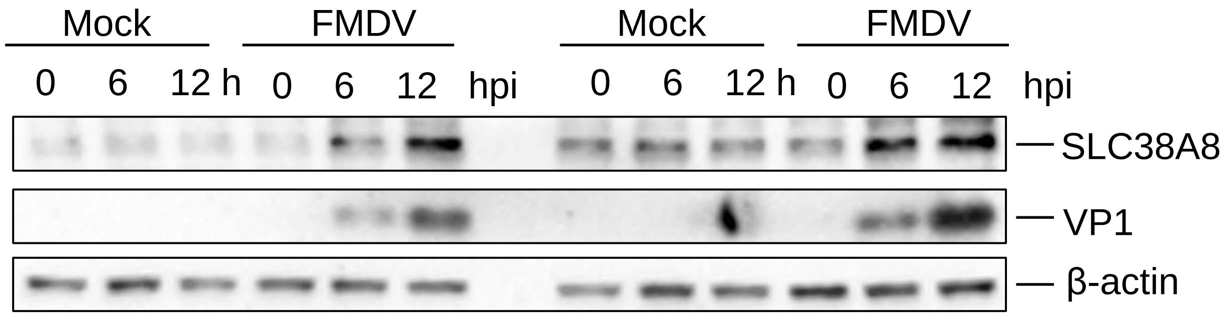

Mock
FMDV
Mock
FMDV
0 6 12 h 0 6 12 hpi
0 6 12 h 0 6 12 hpi
SLC38A8
VP1
β-actin

Fig 6C

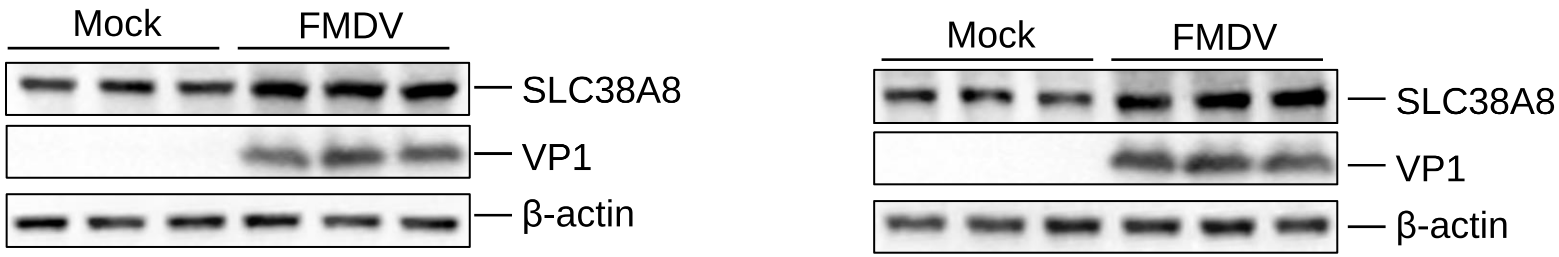

Mock
FMDV
SLC38A8
VP1
β-actin
Mock
FMDV
SLC38A8
VP1
β-actin

Fig 6E

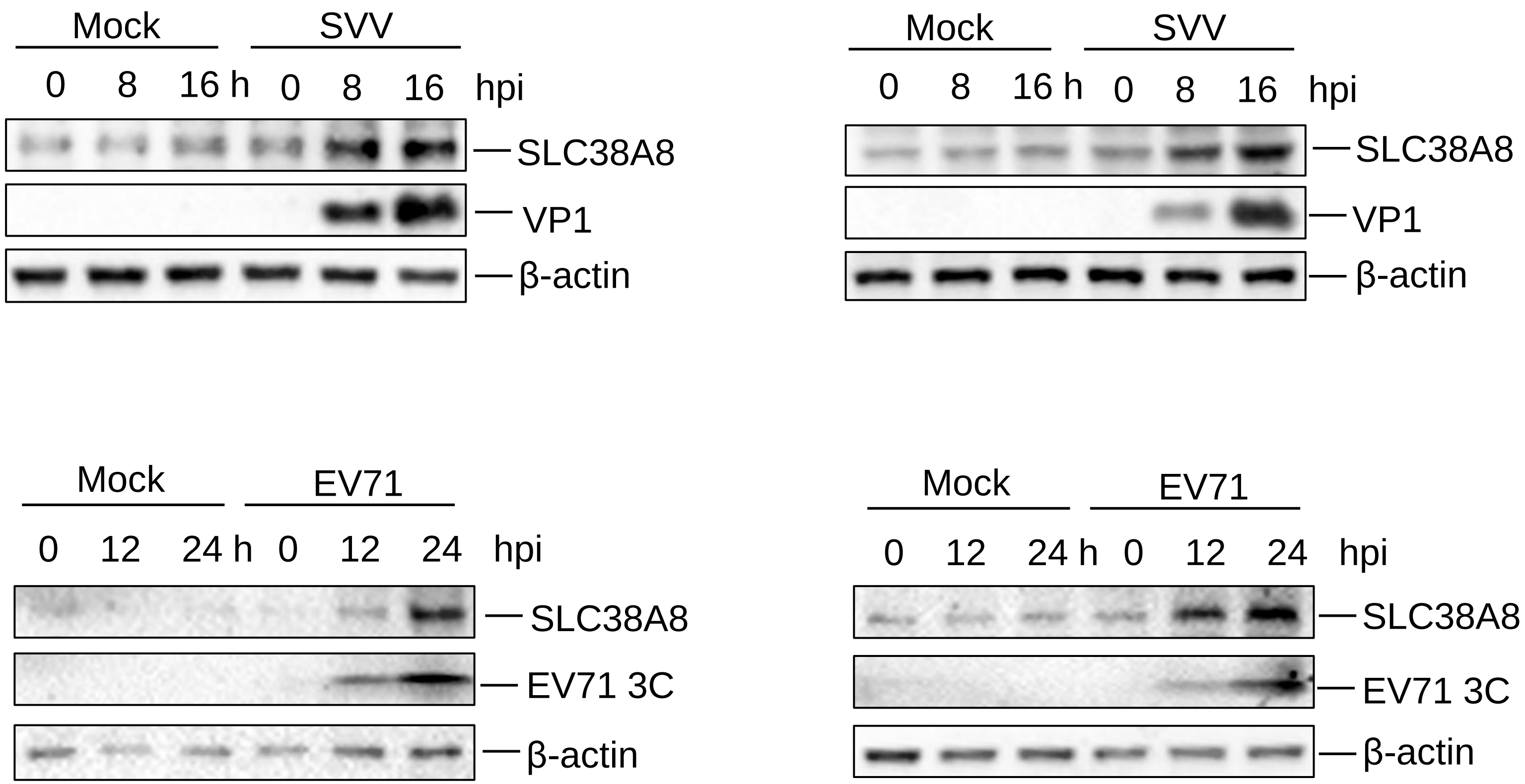
Mock
SVV
0 8 16 h 0 8 16 hpi
SLC38A8
VP1
β-actin
Mock
SVV
0 8 16 h 0 8 16 hpi
SLC38A8
VP1
β-actin
Mock
EV71
0 12 24 h 0 12 24 hpi
SLC38A8
EV71 3C
β-actin
Mock
EV71
0 12 24 h 0 12 24 hpi
SLC38A8
EV71 3C
β-actin

Fig 8A

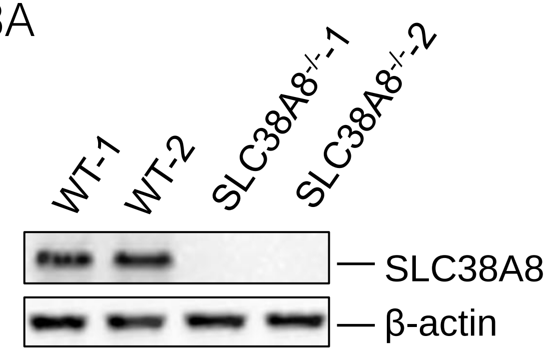

WT-1
WT-2
SLC38A8-/--1
SLC38A8-/--2
SLC38A8
β-actin

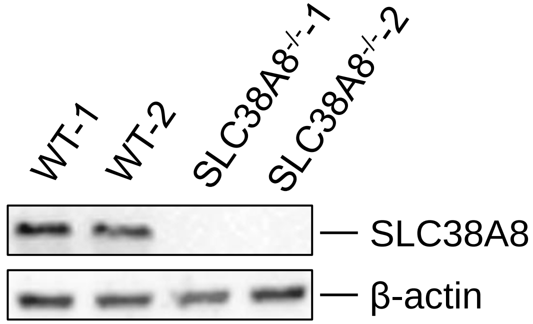

WT-1
WT-2
SLC38A8-/--1
SLC38A8-/--2
SLC38A8
β-actin

Fig 9C

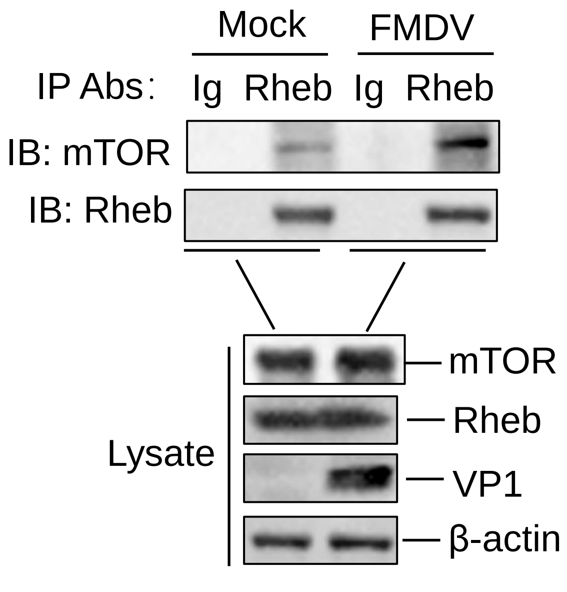
Mock
FMDV
IP Abs:
Ig
Rheb
Ig
Rheb
IB: mTOR
IB: Rheb
Lysate
mTOR
Rheb
VP1
β-actin

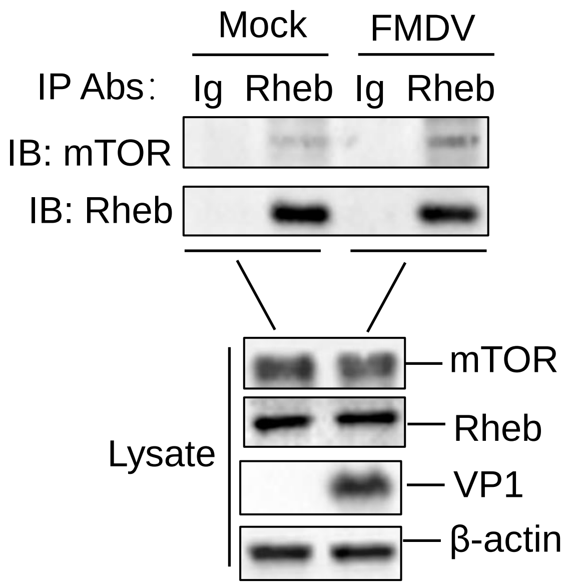
Mock
FMDV
IP Abs:
Ig
Rheb
Ig
Rheb
IB: mTOR
IB: Rheb
Lysate
mTOR
Rheb
VP1
β-actin

Fig 9D

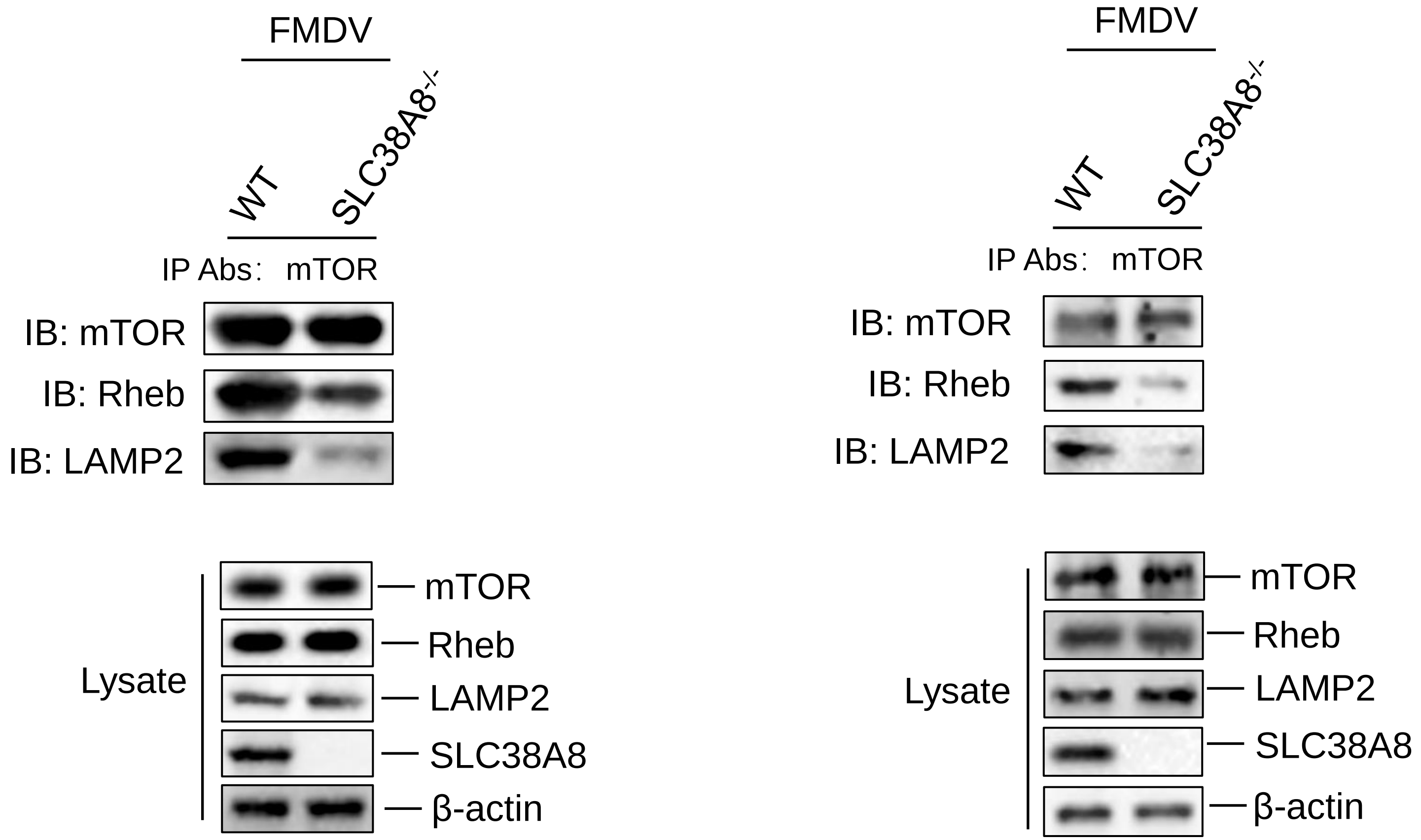
FMDV
WT
SLC38A8-/-
IP Abs: mTOR
IB: mTOR
IB: Rheb
IB: LAMP2
Lysate
mTOR
Rheb
LAMP2
SLC38A8
β-actin
FMDV
WT
SLC38A8-/-
IP Abs: mTOR
IB: mTOR
IB: Rheb
IB: LAMP2
Lysate
mTOR
Rheb
LAMP2
SLC38A8
β-actin

Fig 10A

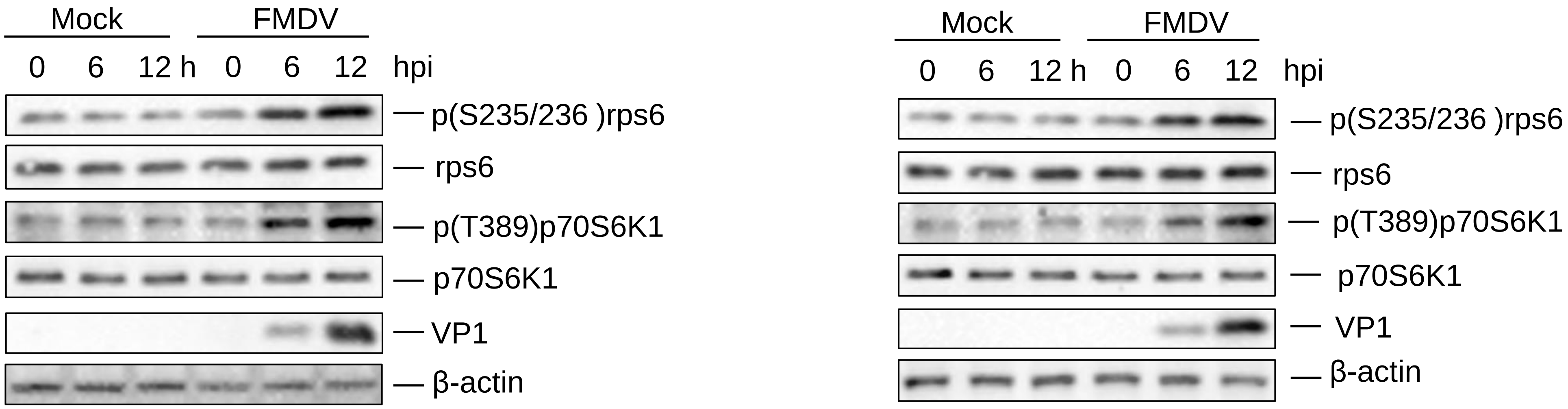
Mock
FMDV
0 6 12 h 0 6 12 hpi
p(S235/236)rps6
rps6
p(T389)p70S6K1
p70S6K1
VP1
β-actin
Mock
FMDV
0 6 12 h 0 6 12 hpi
p(S235/236)rps6
rps6
p(T389)p70S6K1
p70S6K1
VP1
β-actin

Fig 10B

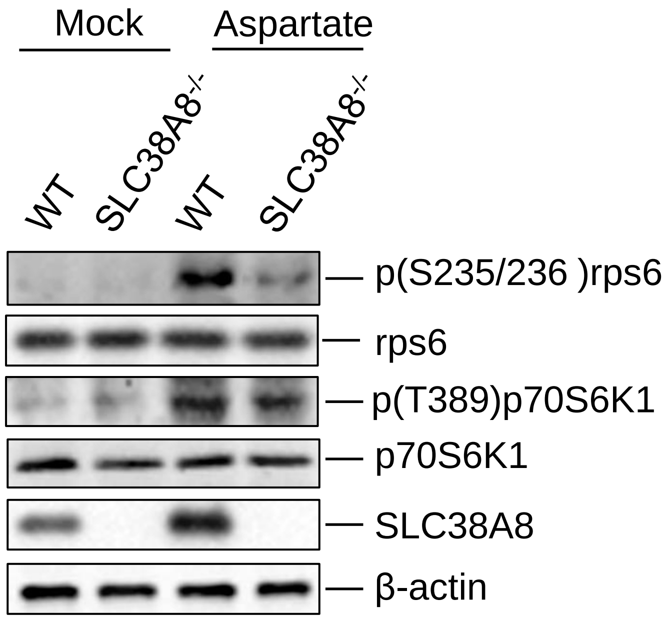

Mock
Aspartate
WT
SLC38A8-/-
WT
SLC38A8-/-
p(S235/236)rps6
rps6
p(T389)p70S6K1
p70S6K1
SLC38A8
β-actin

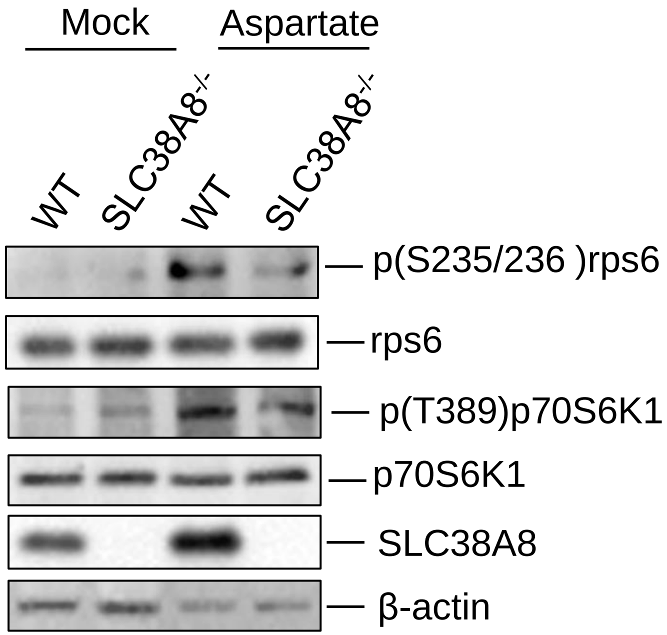

Mock
Aspartate
WT
SLC38A8-/-
WT
SLC38A8-/-
p(S235/236)rps6
rps6
p(T389)p70S6K1
p70S6K1
SLC38A8
β-actin

Fig 10C

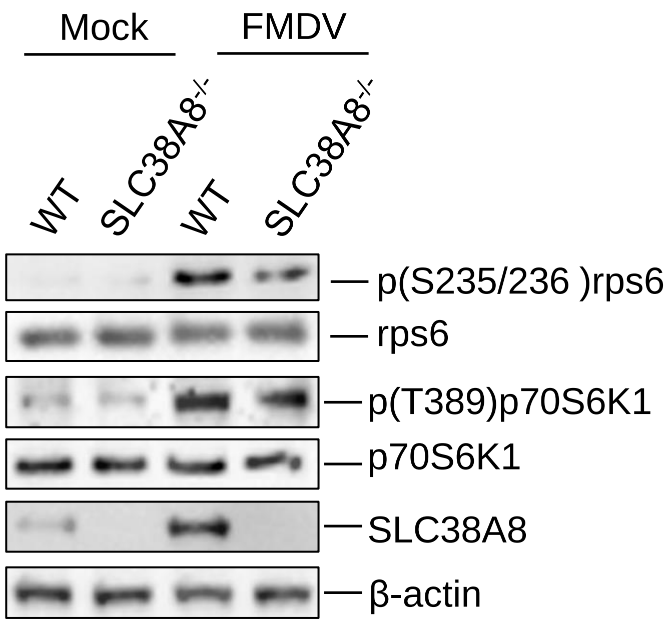
Mock
FMDV
WT
SLC38A8-/-
WT
SLC38A8-/-
p(S235/236)rps6
rps6
p(T389)p70S6K1
p70S6K1
SLC38A8
β-actin

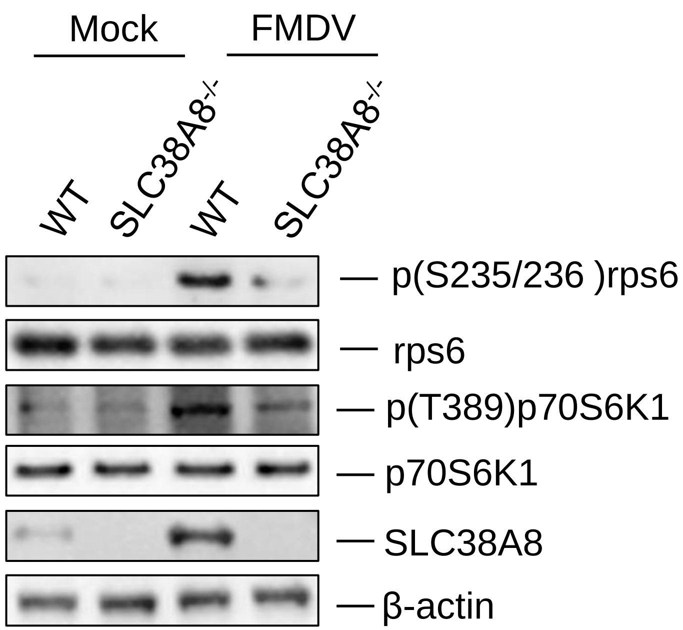
Mock
FMDV
WT
SLC38A8-/-
WT
SLC38A8-/-
p(S235/236)rps6
rps6
p(T389)p70S6K1
p70S6K1
SLC38A8
β-actin

Fig 10D

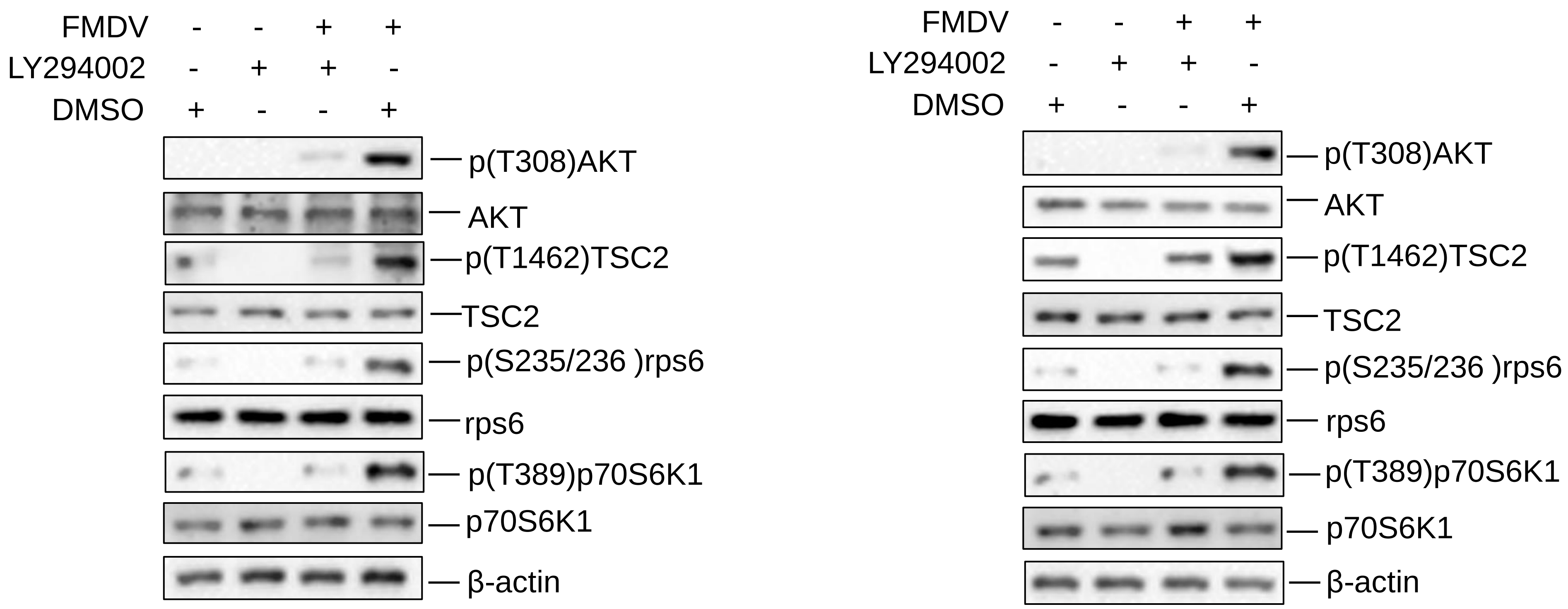
FMDV - - + +
LY294002 - + + -
DMSO + - - +
p(T308)AKT
AKT
p(T1462)TSC2
TSC2
p(S235/236)rps6
rps6
p(T389)p70S6K1
p70S6K1
β-actin
FMDV - - + +
LY294002 - + + -
DMSO + - - +
p(T308)AKT
AKT
p(T1462)TSC2
TSC2
p(S235/236)rps6
rps6
p(T389)p70S6K1
p70S6K1
β-actin

Fig 10E

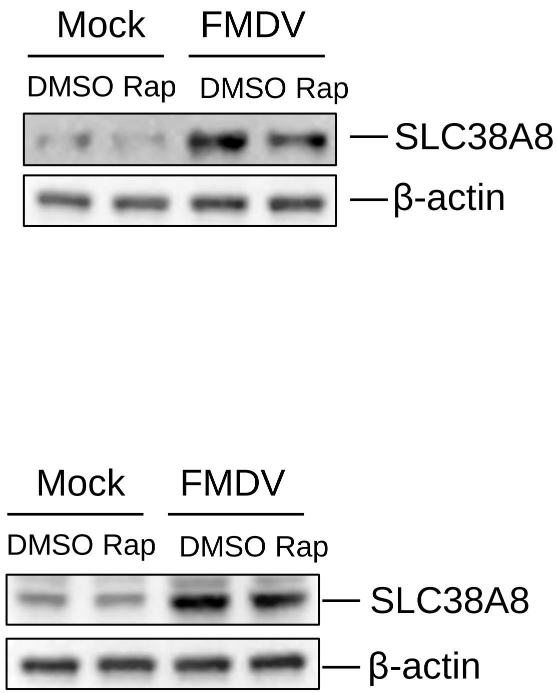

Fig 10G

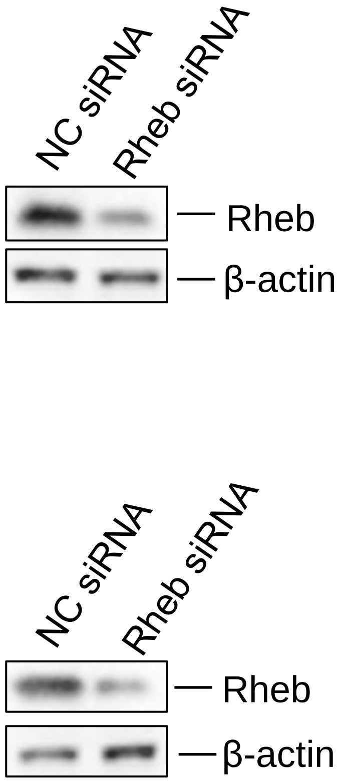

Supplement: S2 Data — (PDF) [file ppat.1011126.s013.pdf]
