# Supplementary material for: Picornavirus infection enhances aspartate by the SLC38A8 transporter to promote viral replication
Source: PLoS Pathog. 2023 Feb 3;19(2):e1011126. doi: 10.1371/journal.ppat.1011126 (PMC9931120; doi:10.1371/journal.ppat.1011126)
Supplement: S3 Data — (PDF) [file ppat.1011126.s014.pdf]

Fig 1A

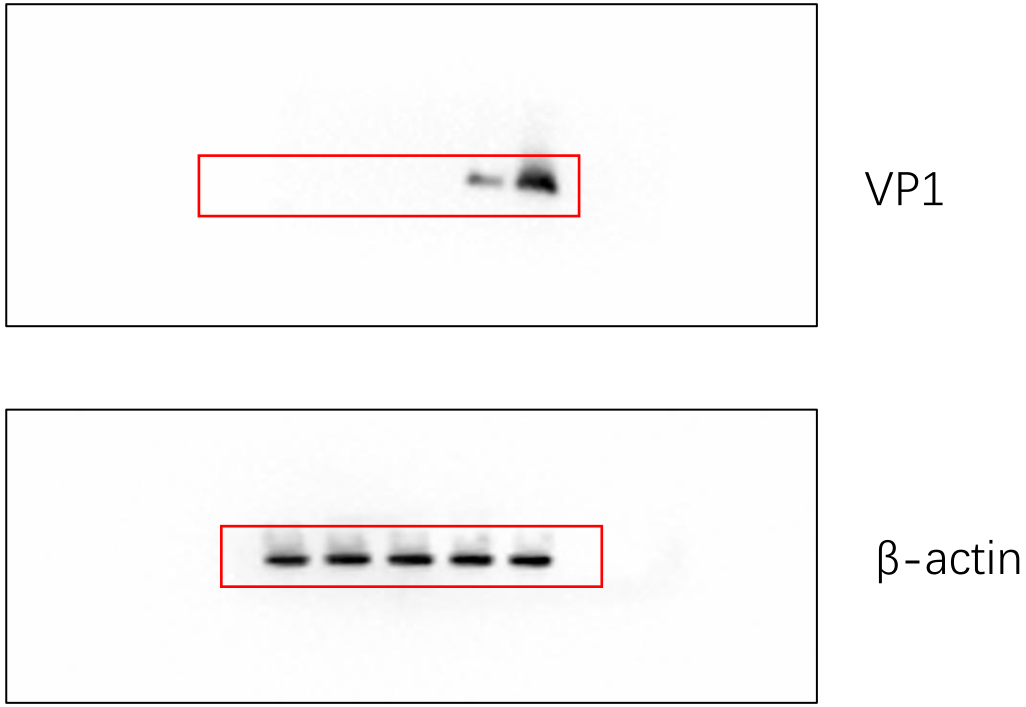

Fig 6B

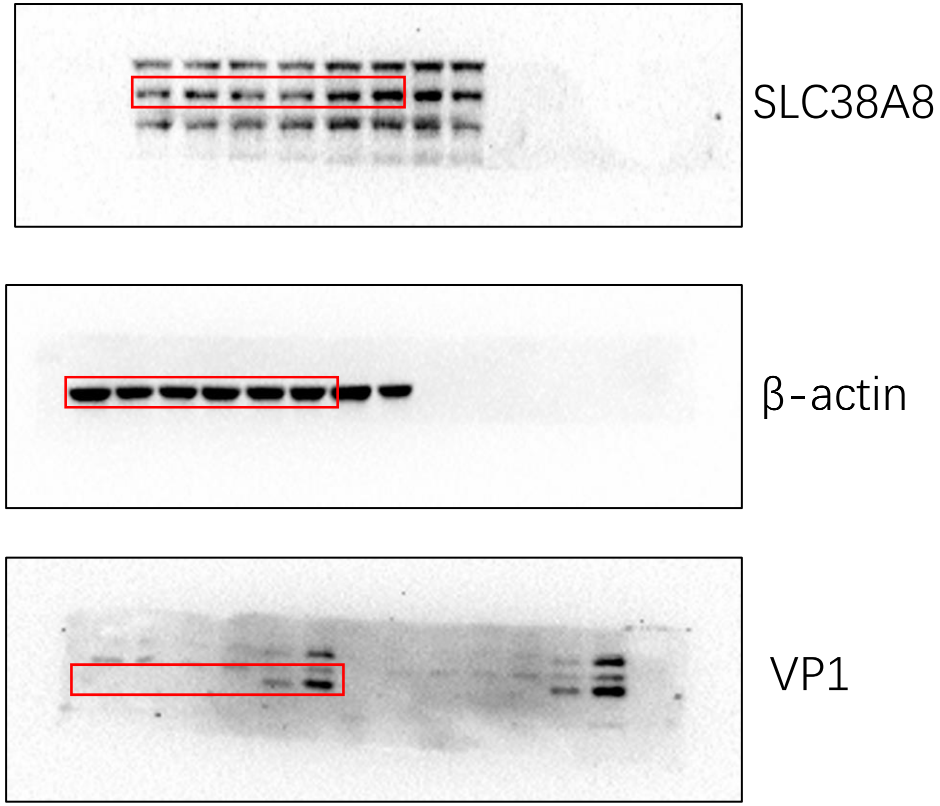

Fig 6C

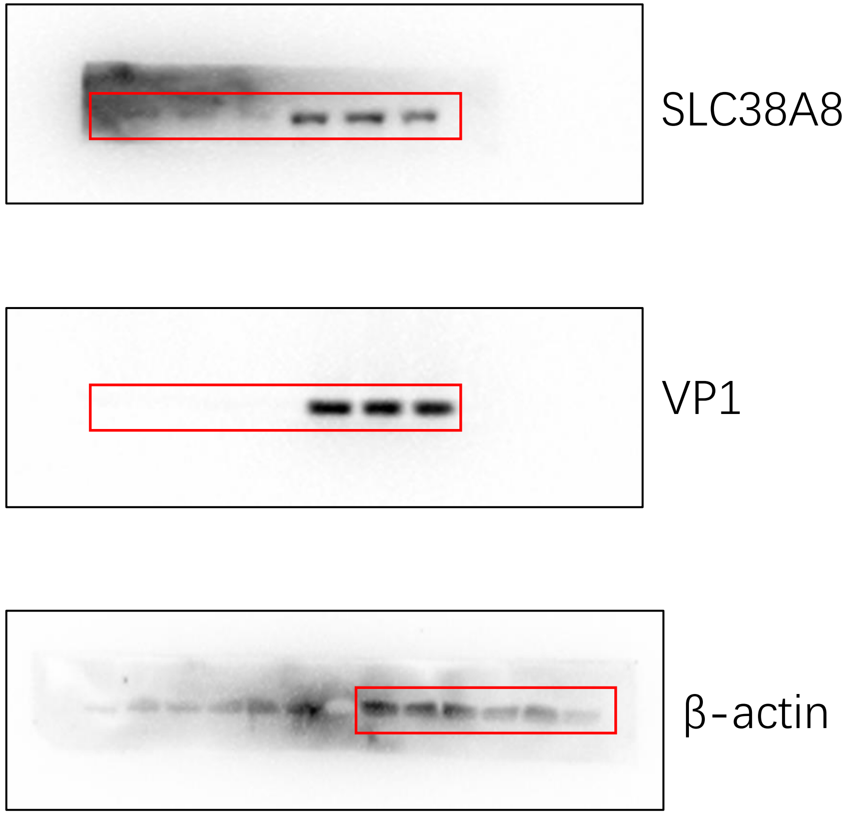

Fig 6E

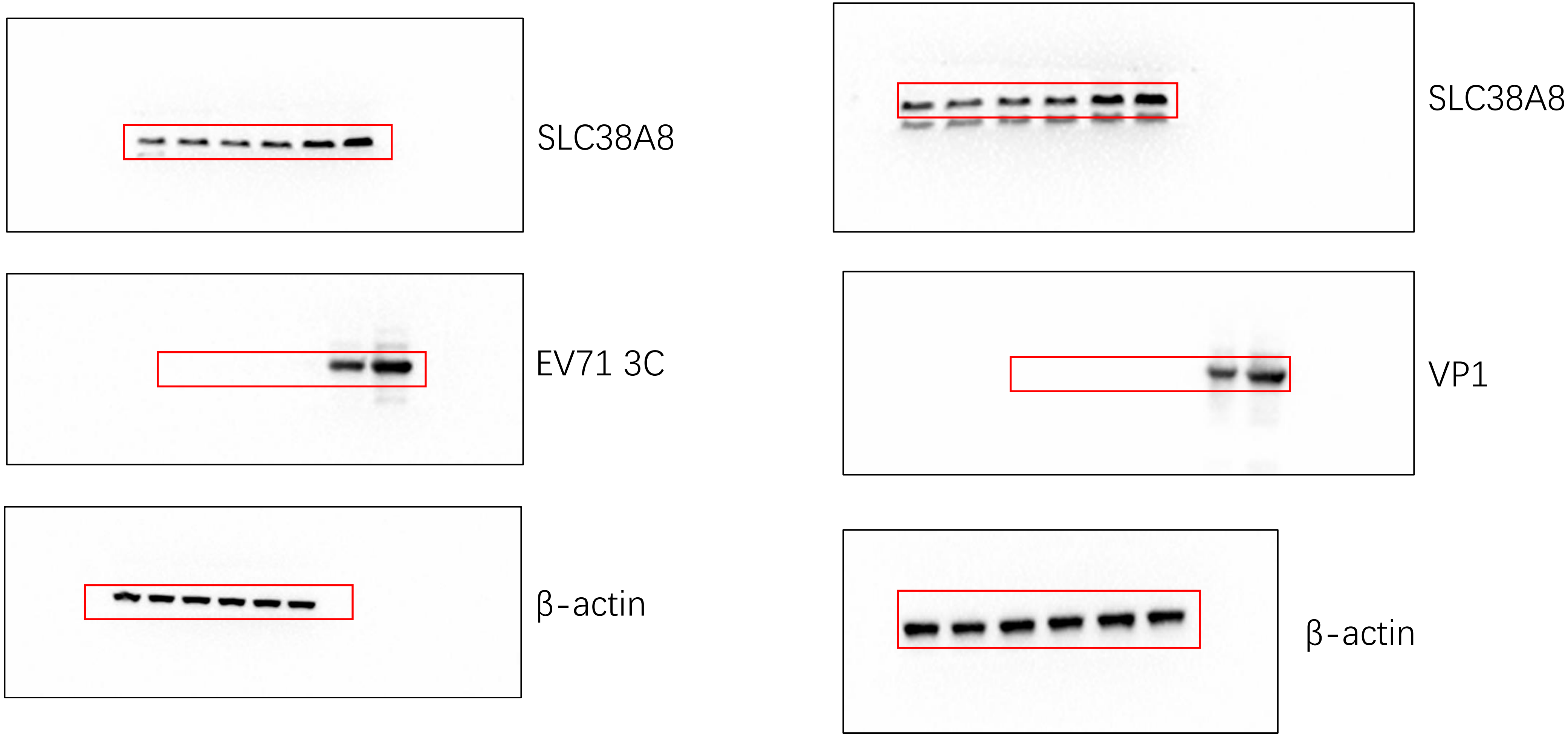

Fig 7A

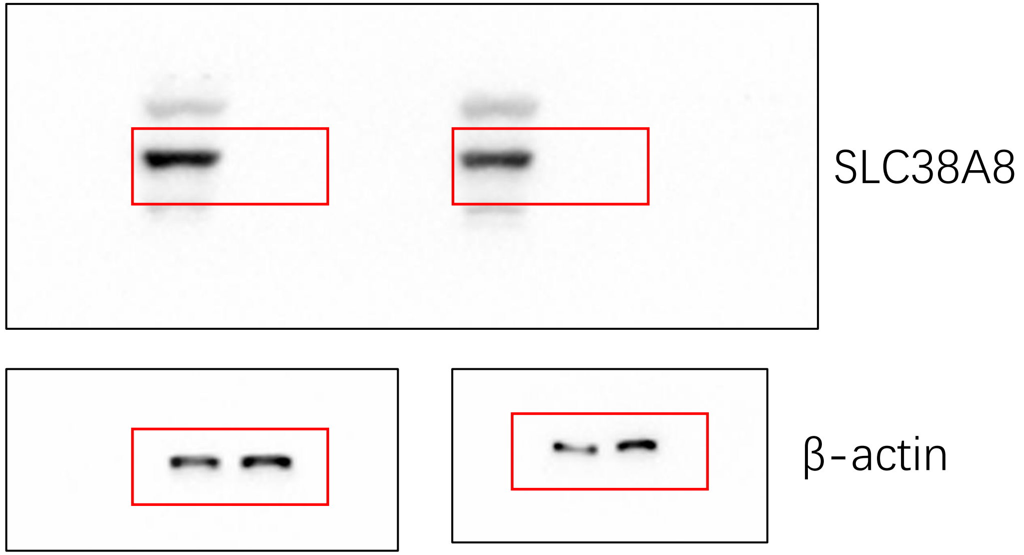

Fig 8A

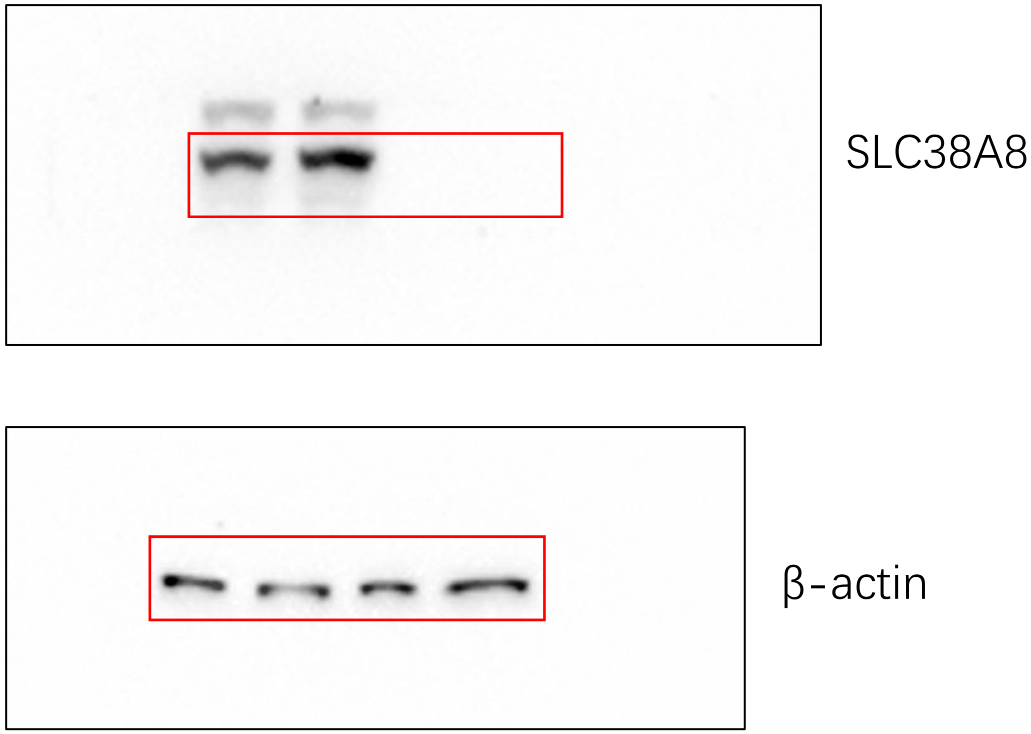

Fig 9C

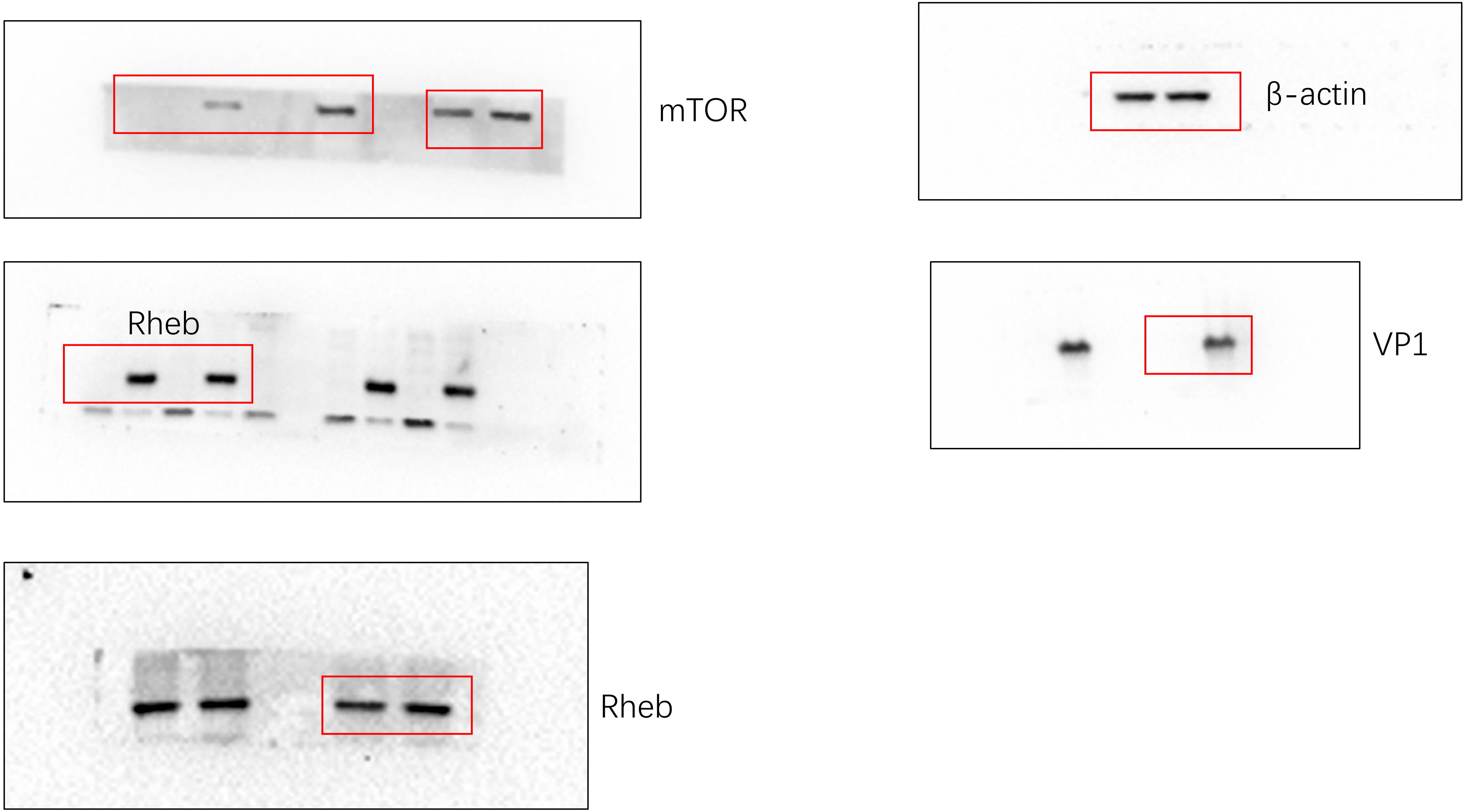

Fig 9D

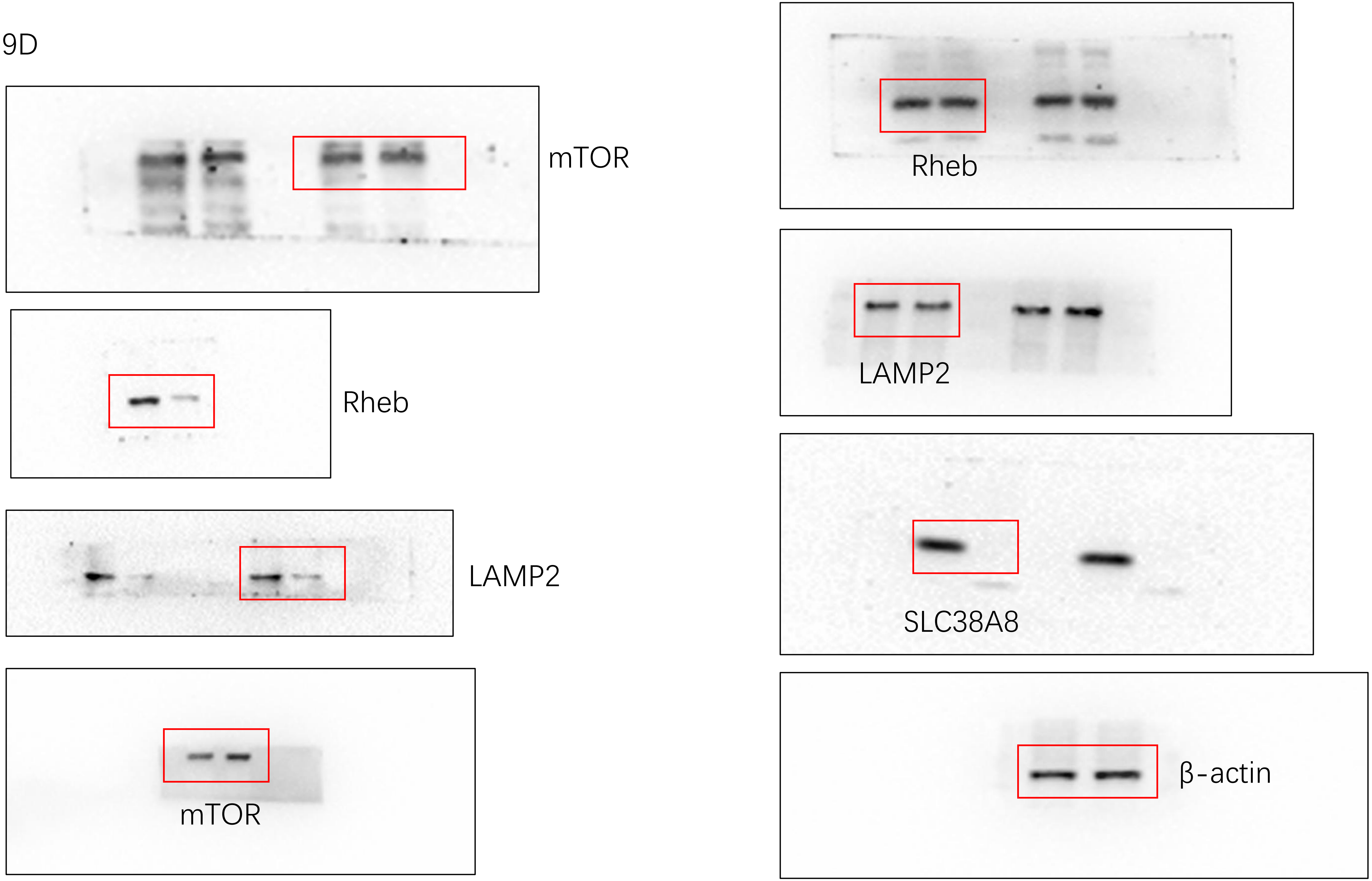

Fig 10A

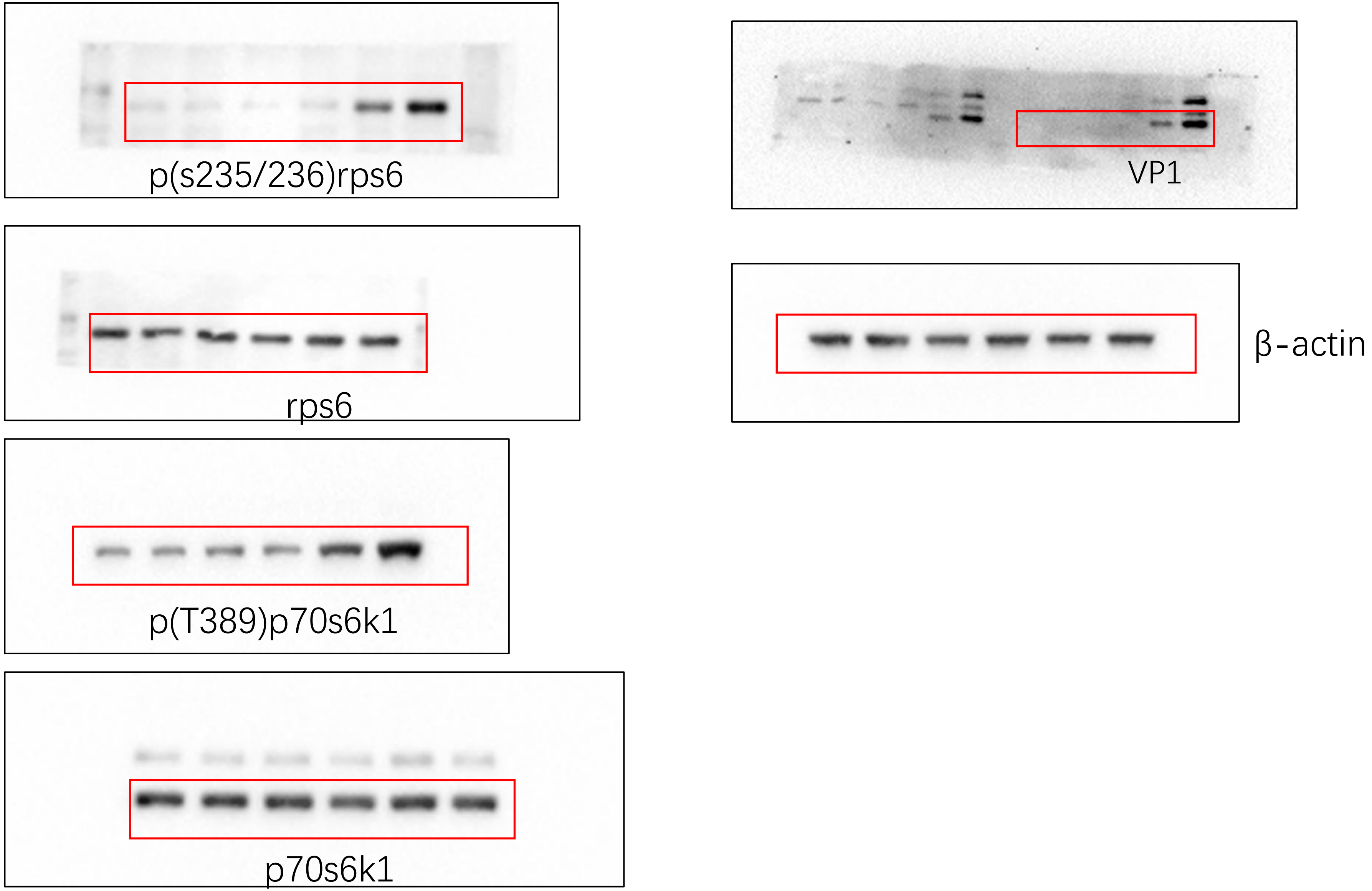

Fig 10B

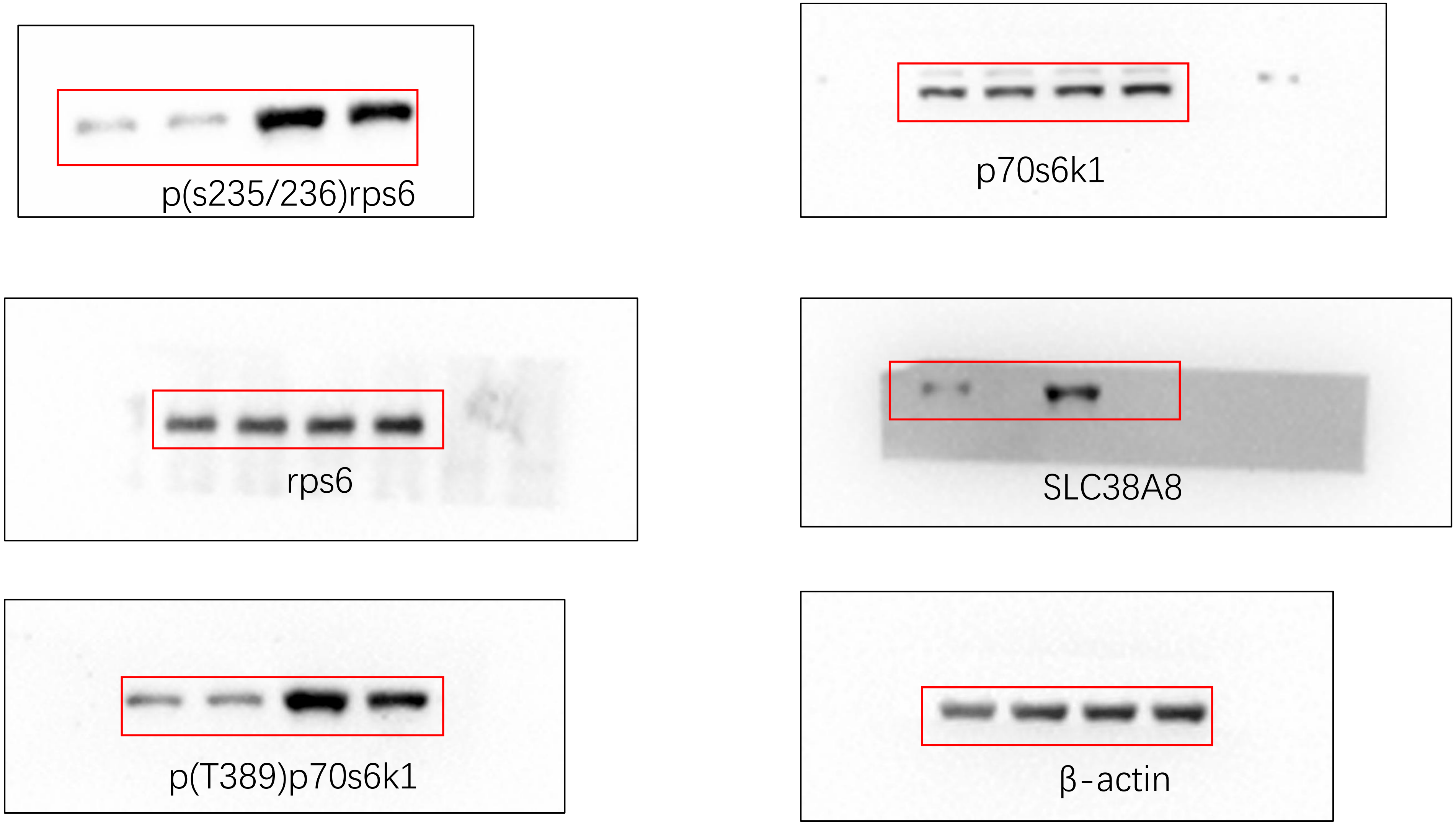

Fig 10C

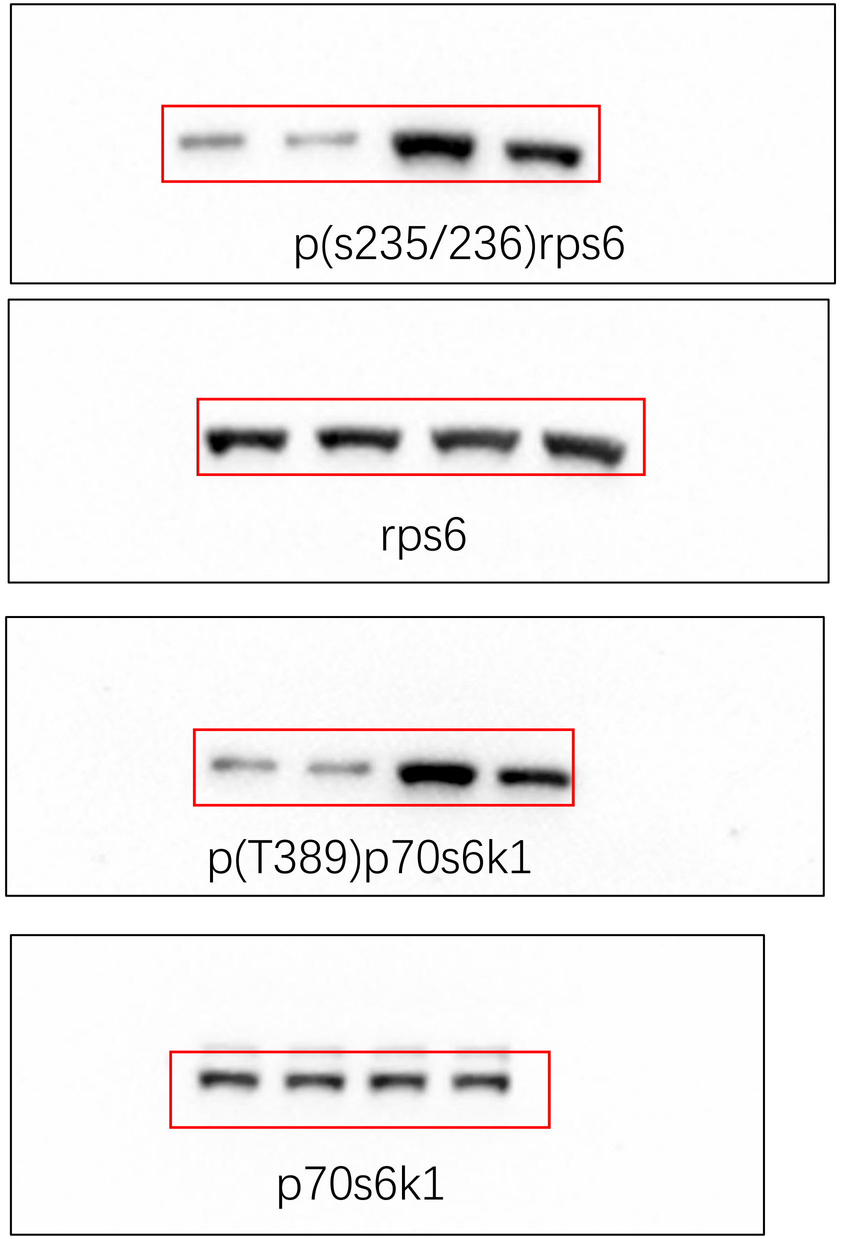

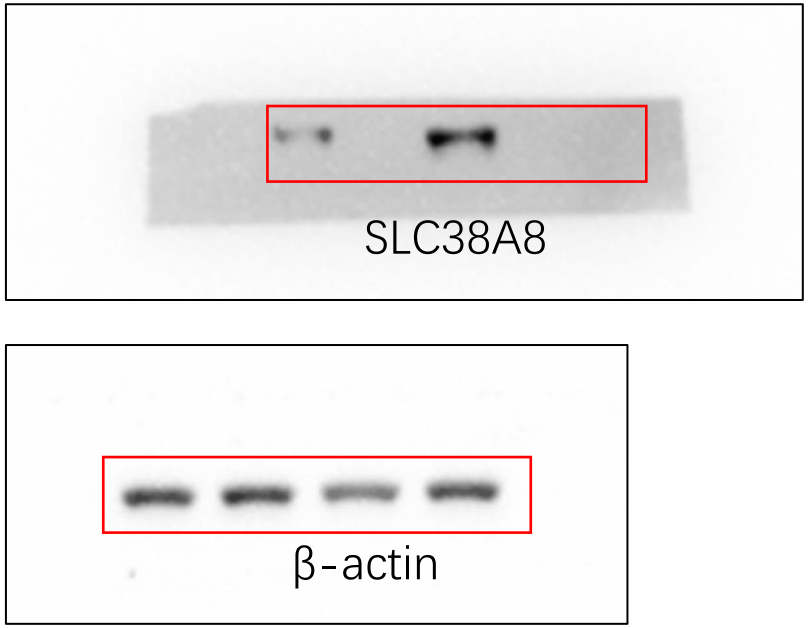

Fig 10E

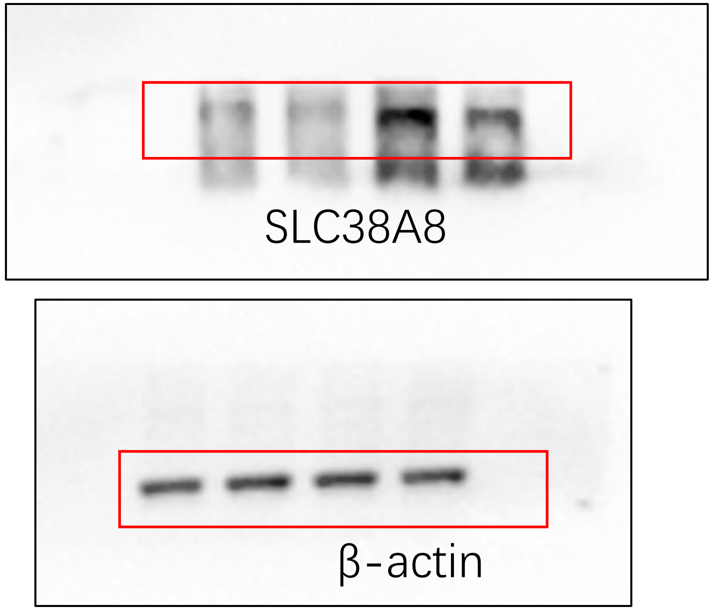

Fig 10G

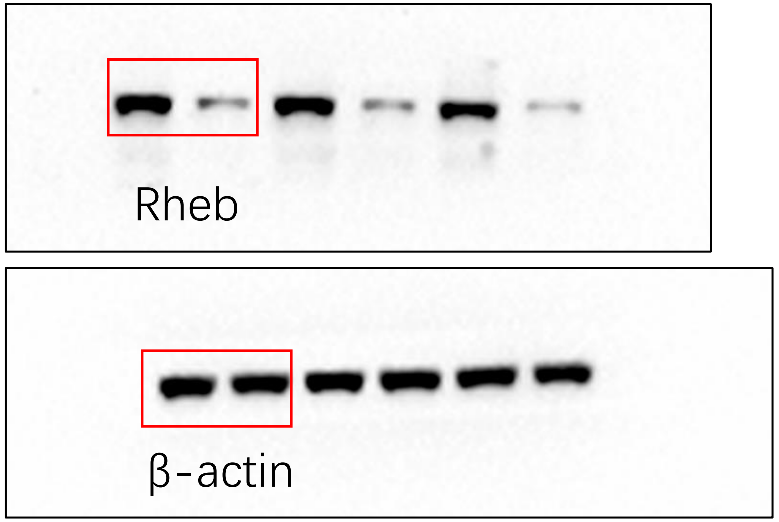

Fig 10D

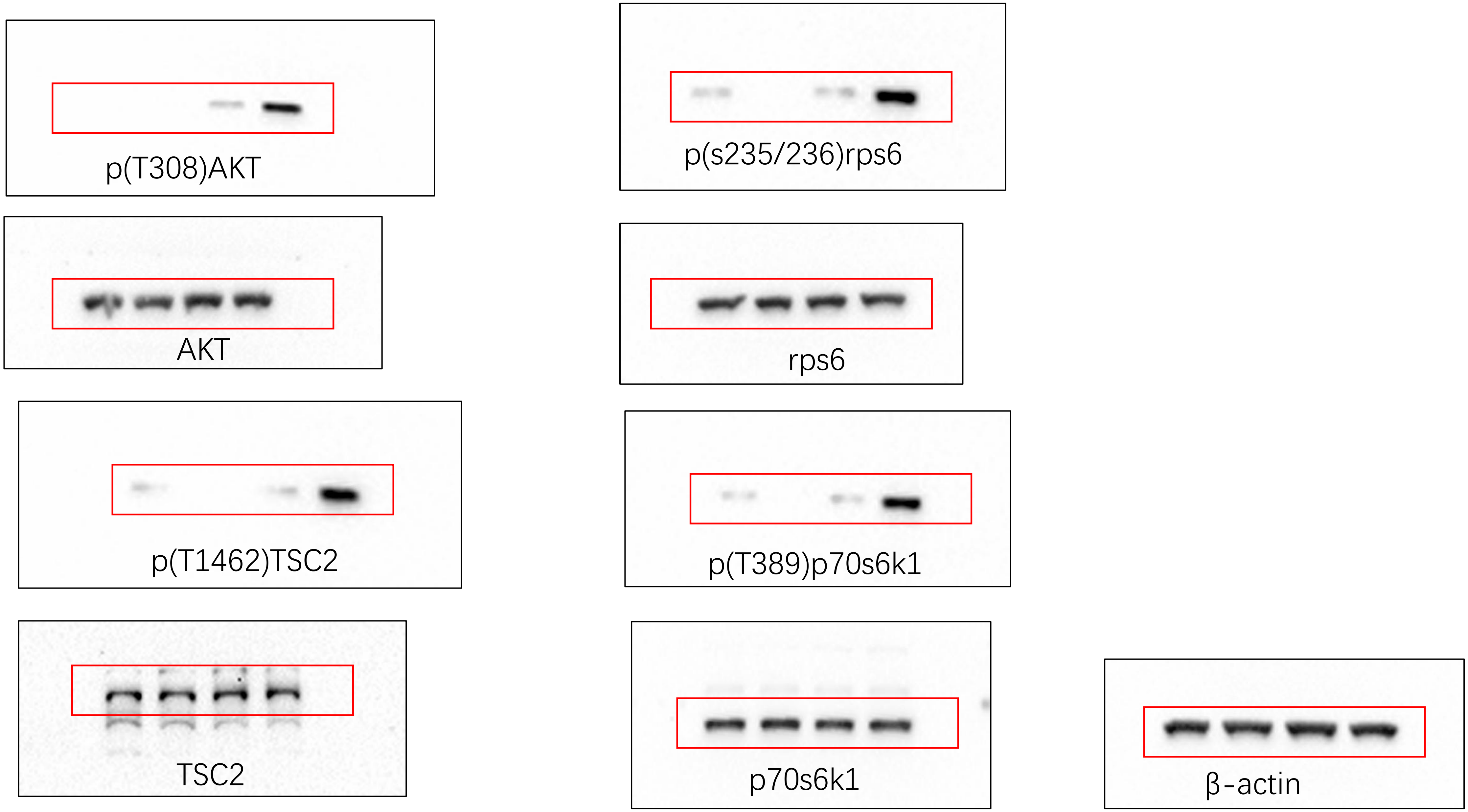
p(T308)AKT
p(s235/236)rps6
AKT
rps6
p(T1462)TSC2
p(T389)p70s6k1
TSC2
p70s6k1
β-actin
