# Supplementary material for: Picornavirus infection enhances aspartate by the SLC38A8 transporter to promote viral replication
Source: PLoS Pathog. 2023 Feb 3;19(2):e1011126. doi: 10.1371/journal.ppat.1011126 (PMC9931120; doi:10.1371/journal.ppat.1011126)

Fig 6B

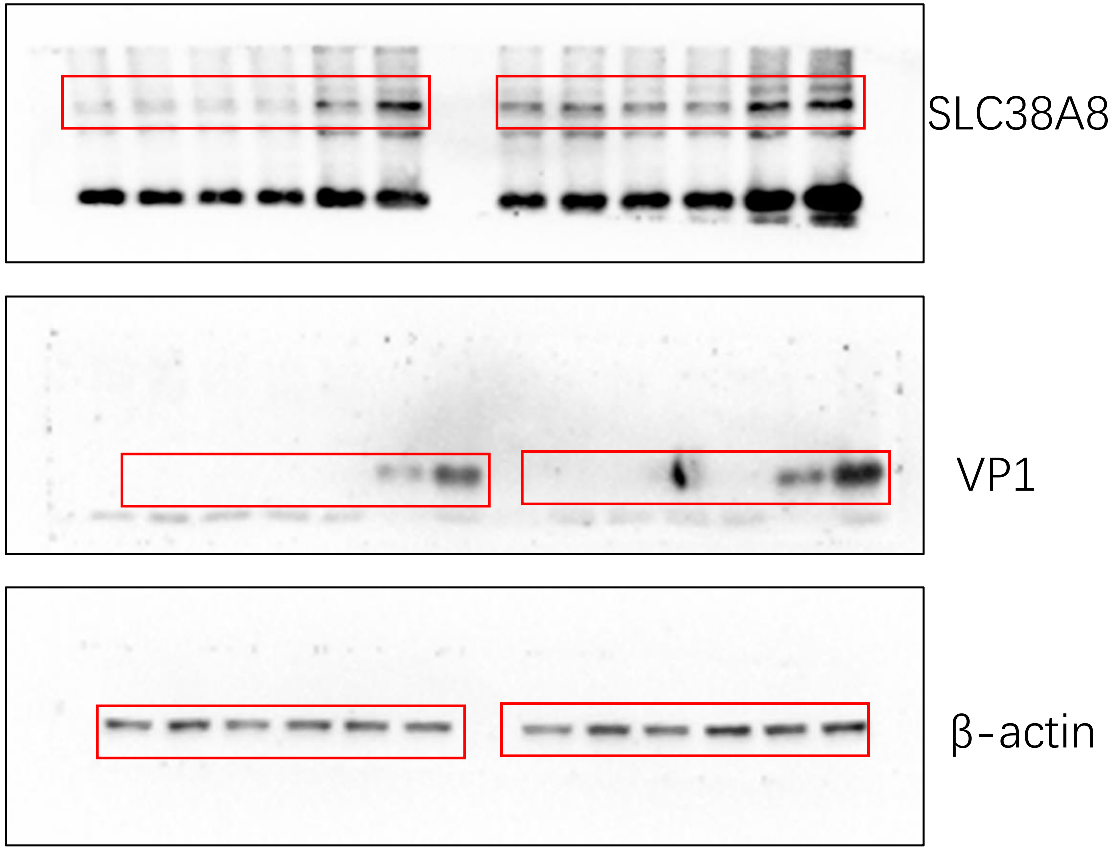

Fig 6C

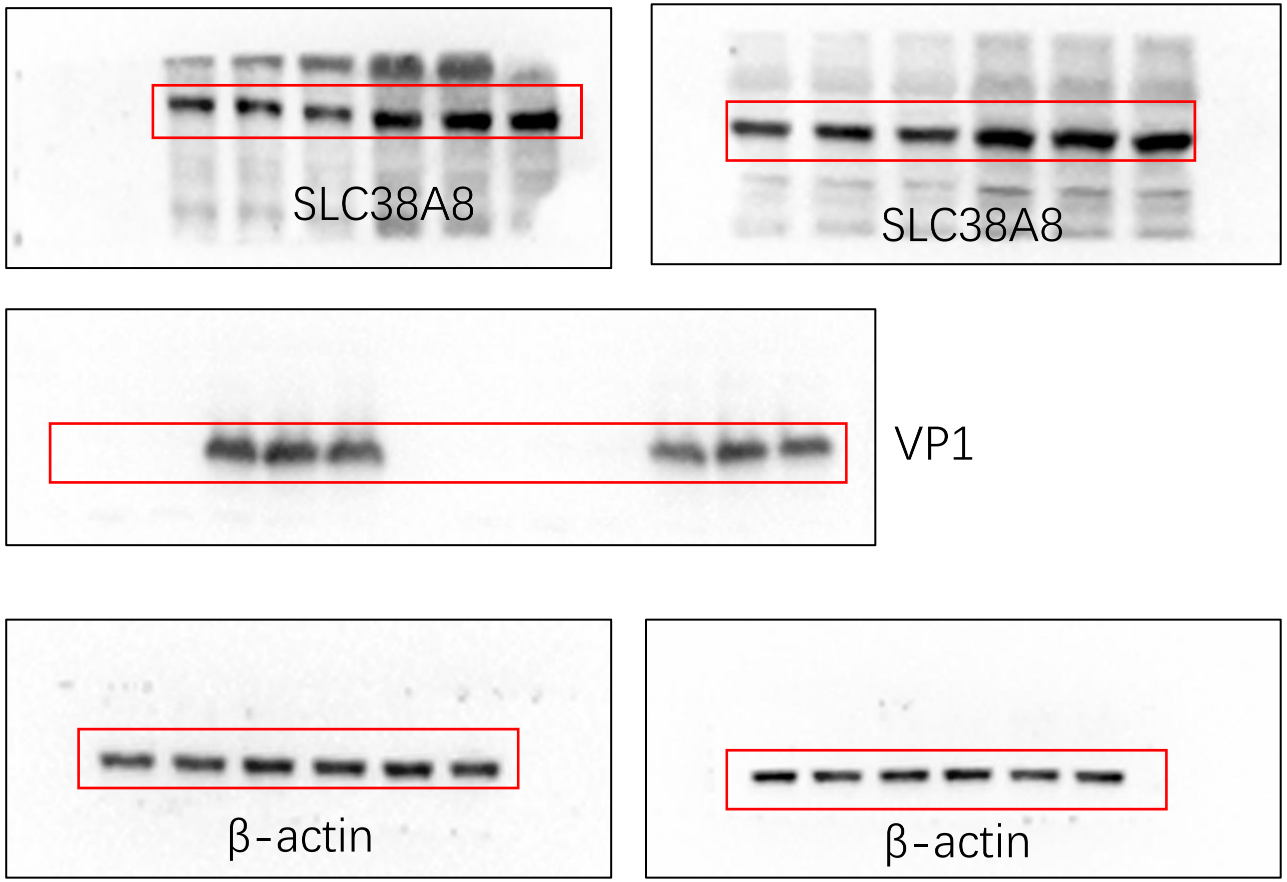

Fig 6E (EV71)

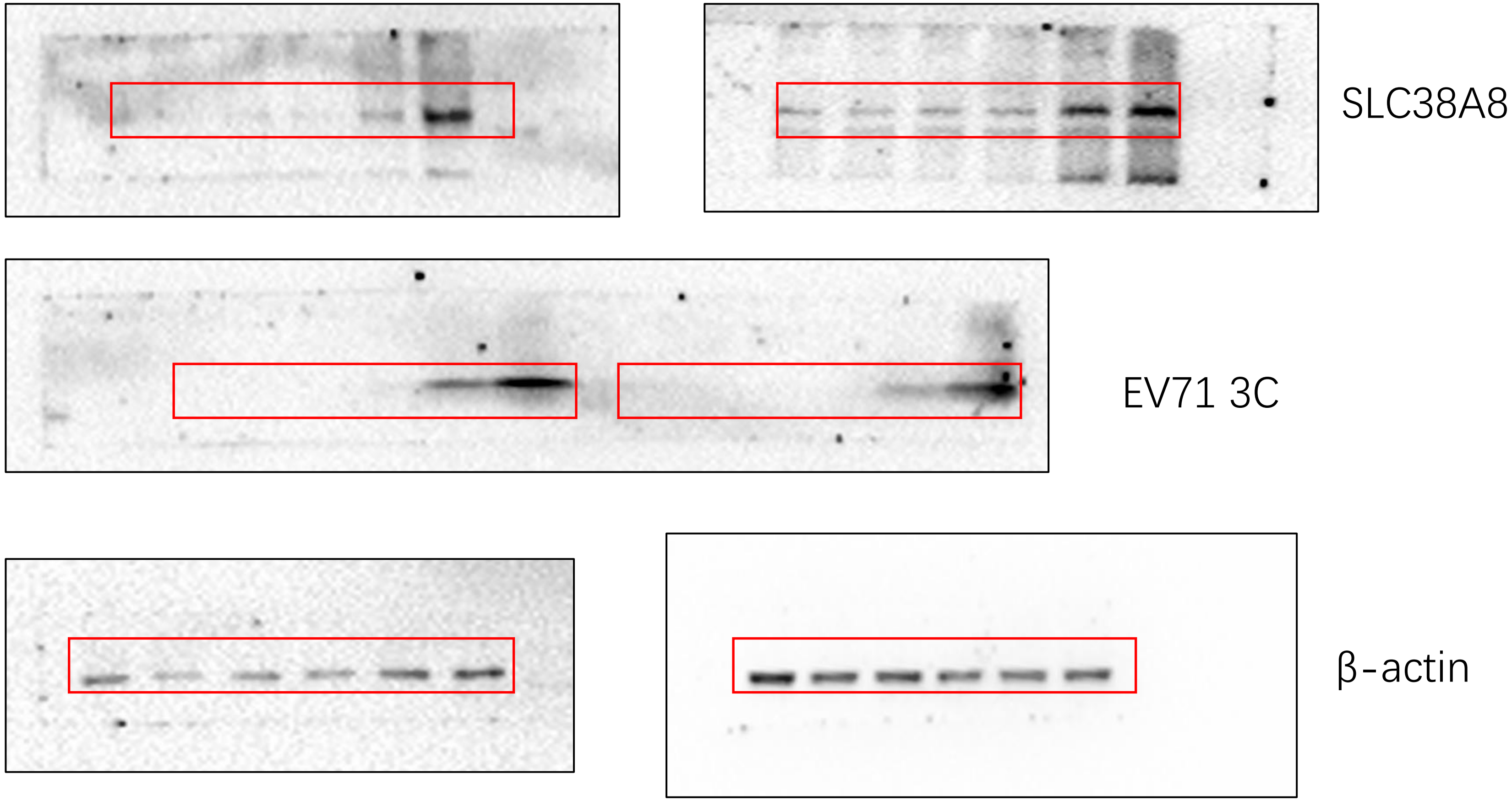

Fig 6E (SVV)

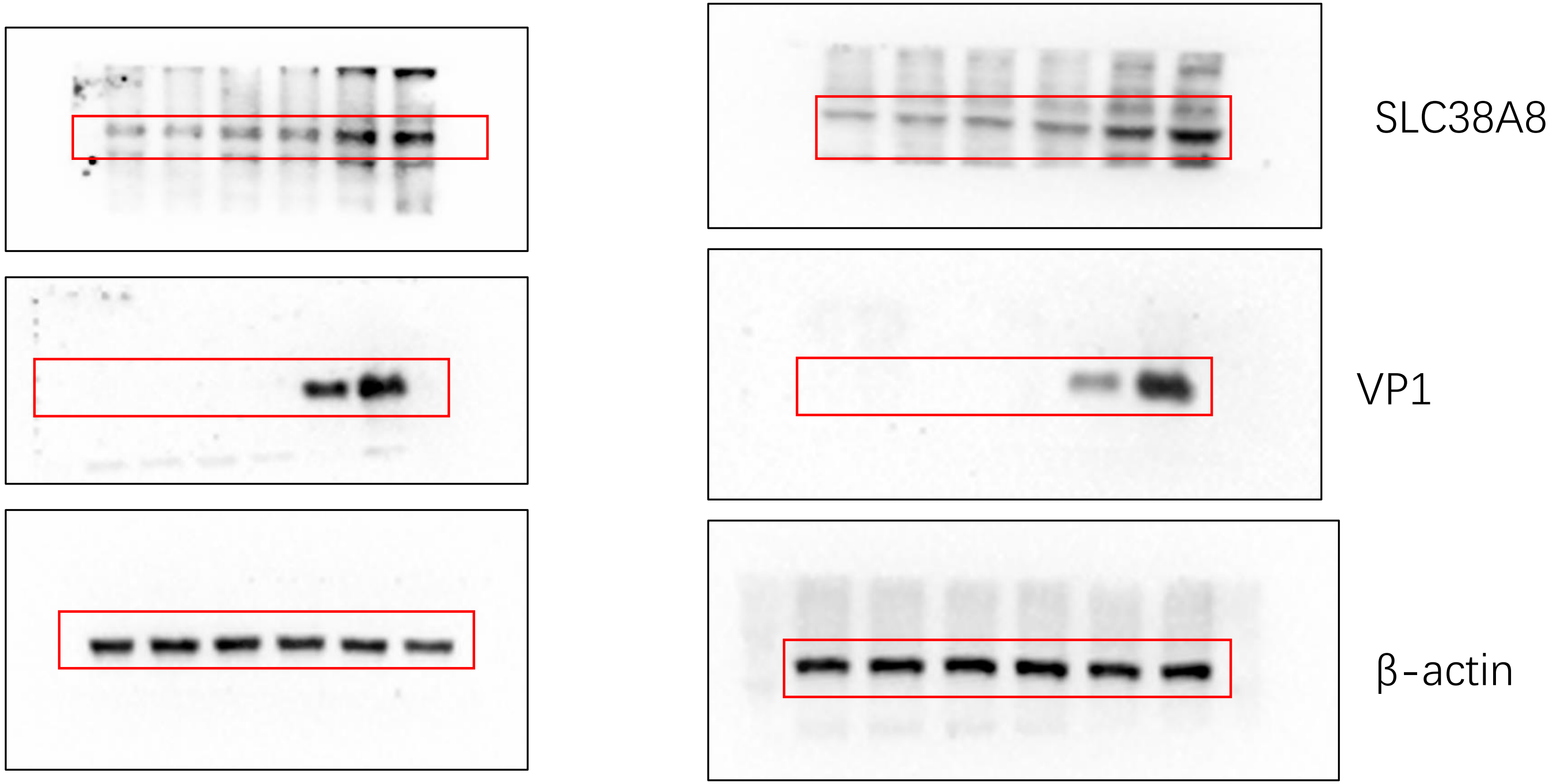

Fig 8A

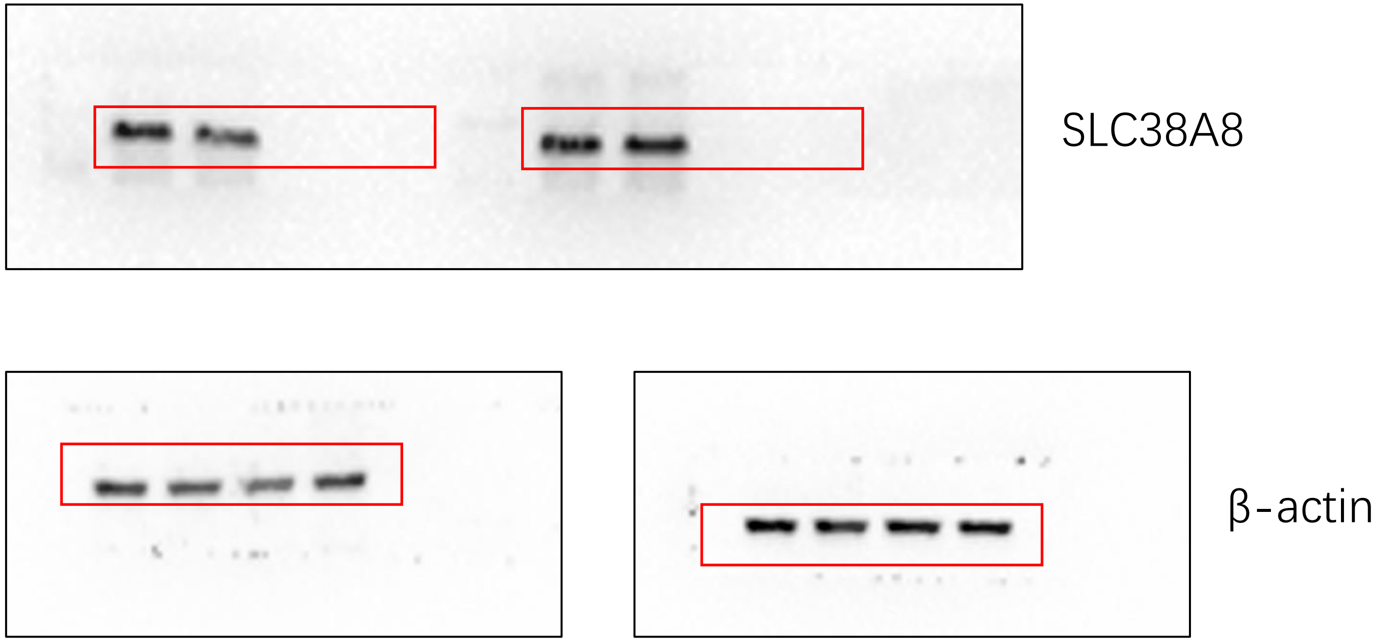

Fig 9C

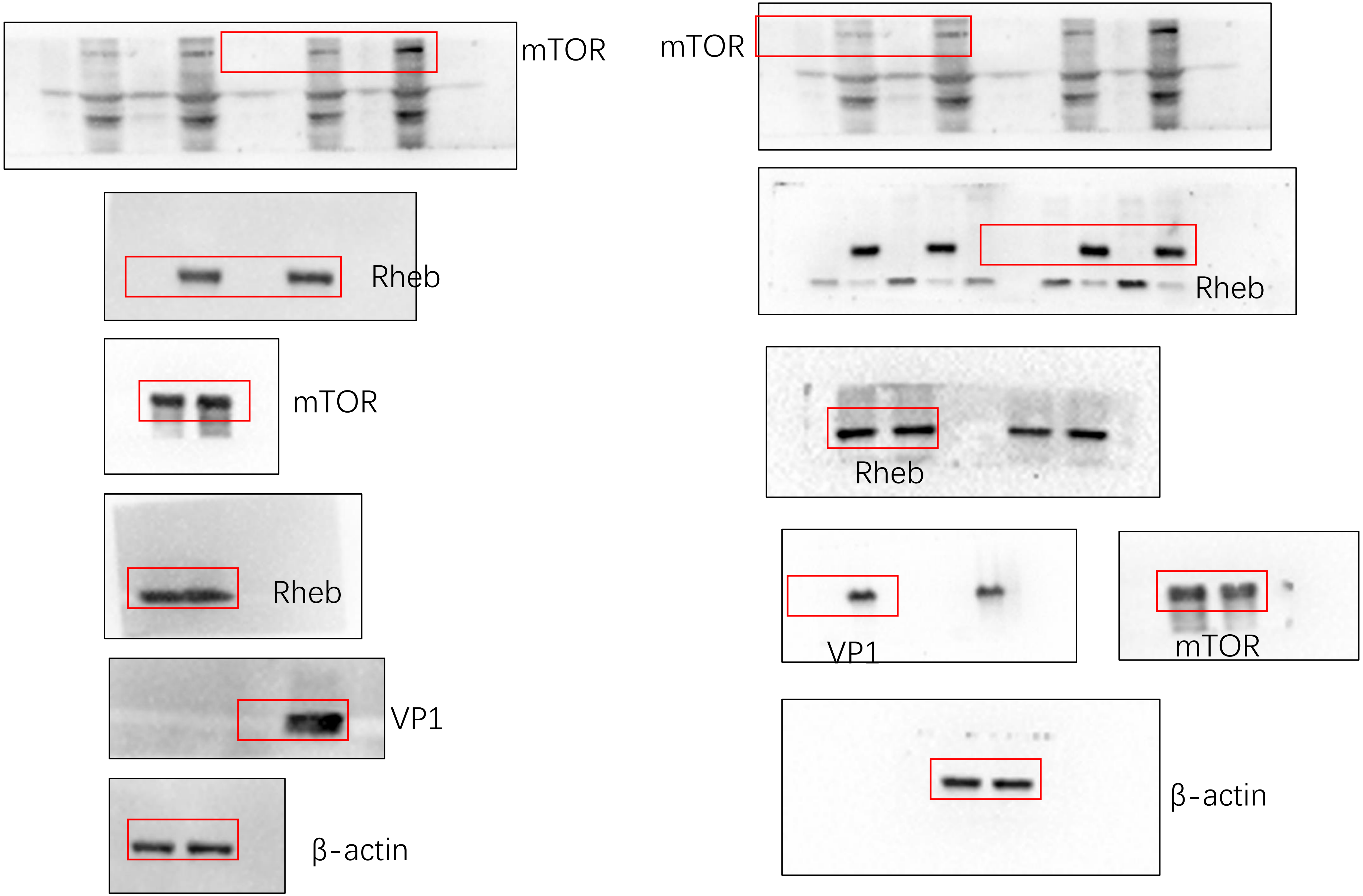
mTOR
Rheb
mTOR
Rheb
VP1
β-actin
mTOR
Rheb
Rheb
VP1
mTOR
β-actin

Fig 9D

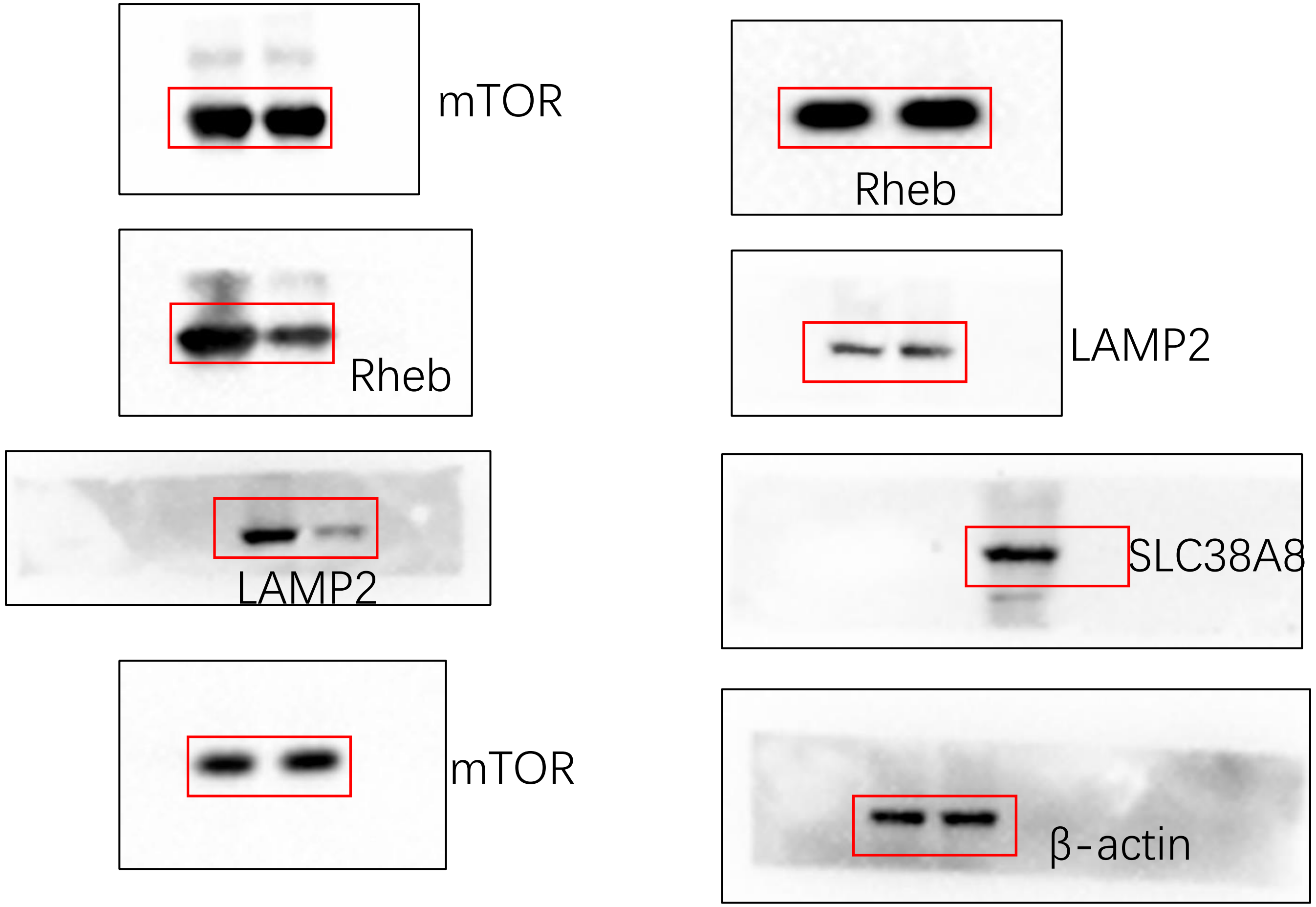

Fig 9D

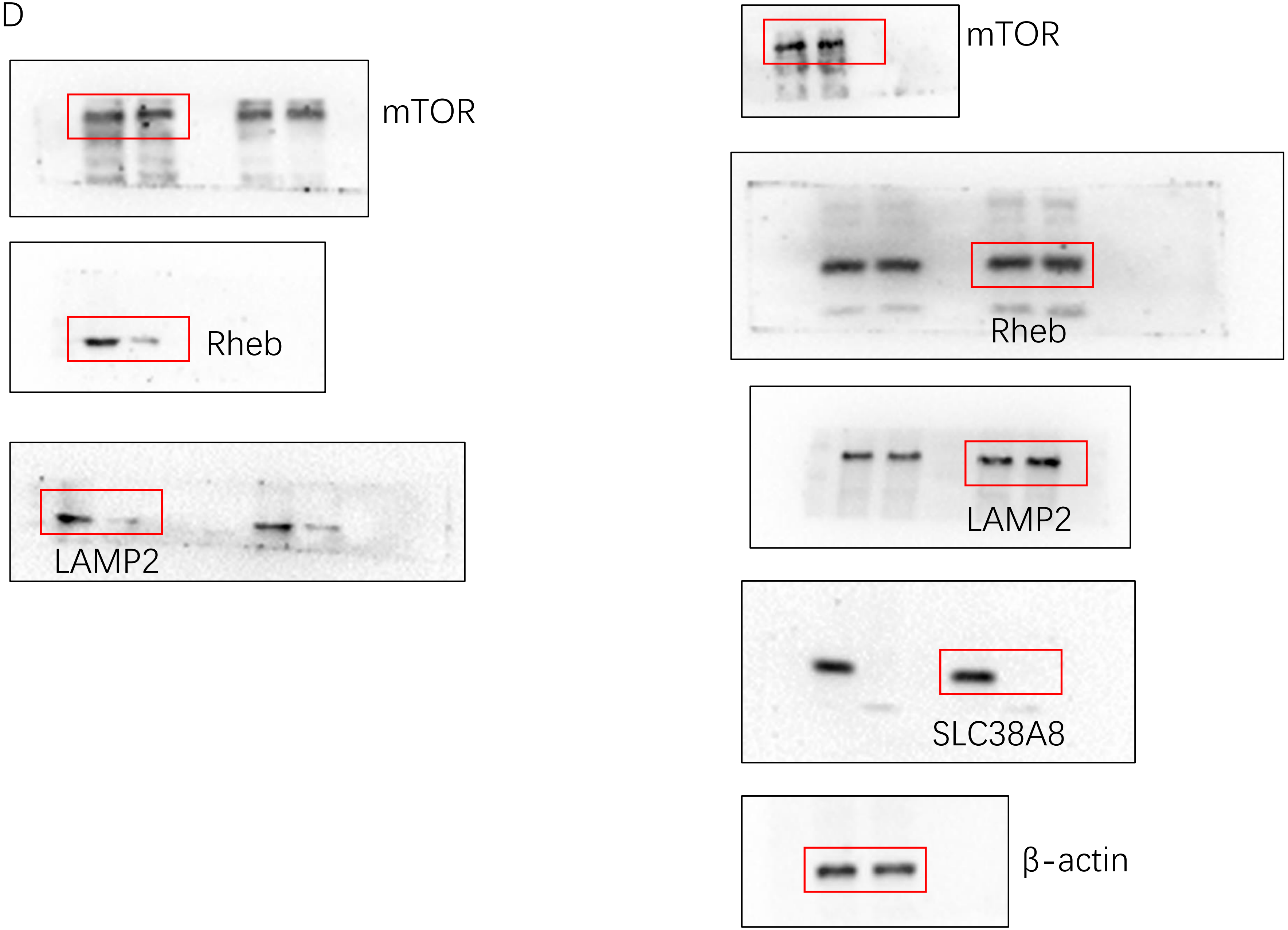

Fig 10A

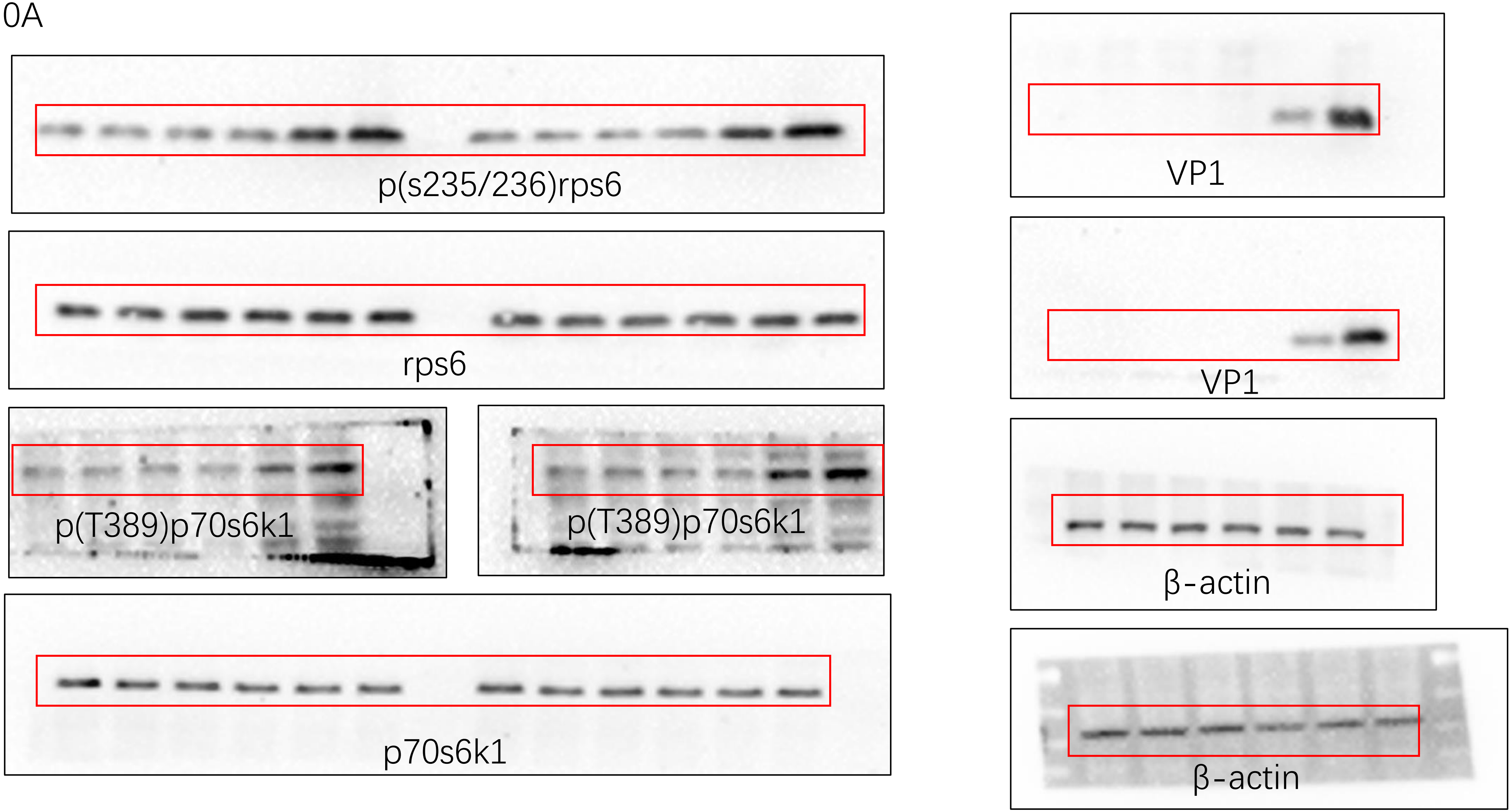

p(s235/236)rps6
rps6
p(T389)p70s6k1
p(T389)p70s6k1
p70s6k1
VP1
VP1
β-actin
β-actin

Fig 10B

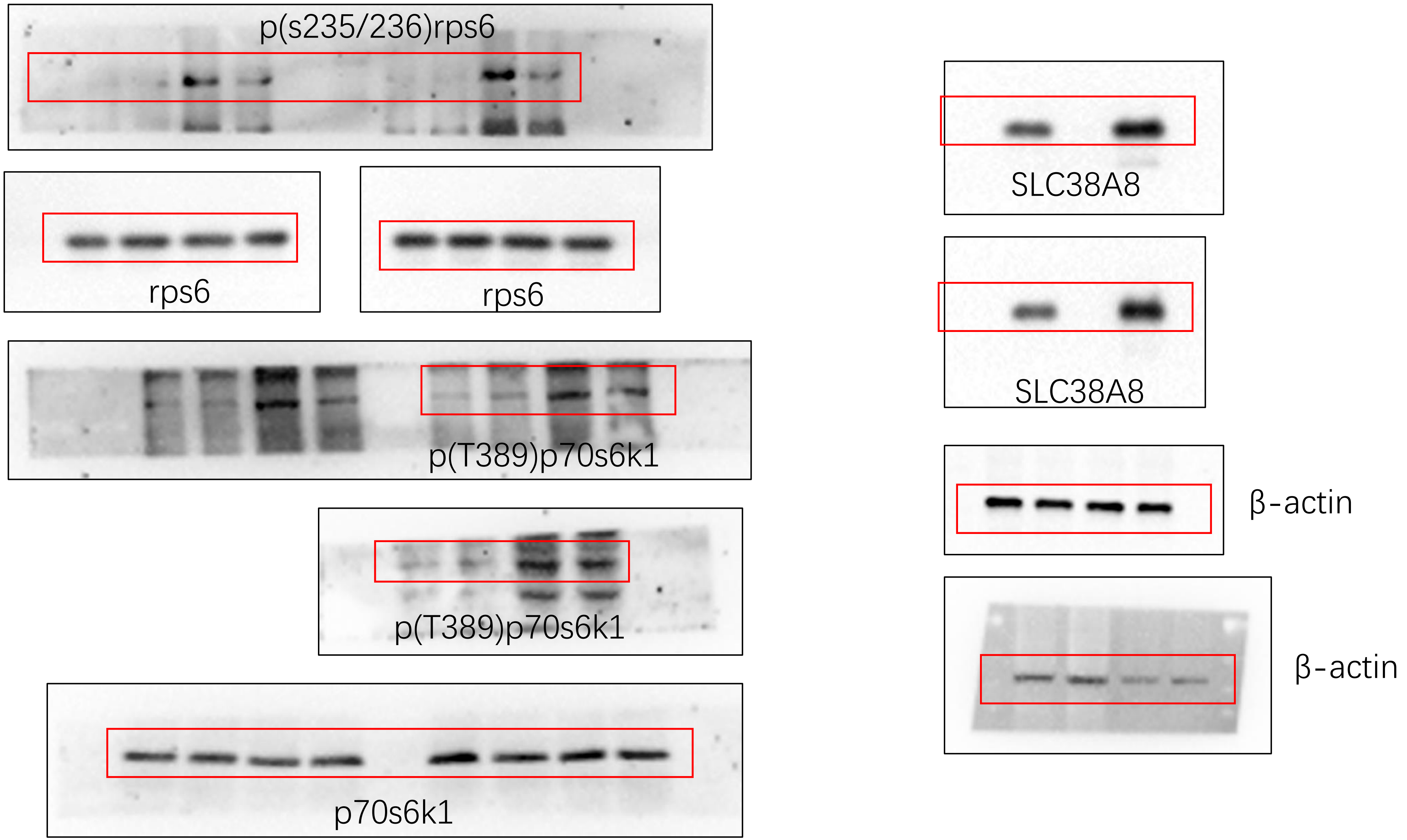
p(s235/236)rps6
rps6
rps6
p(T389)p70s6k1
p(T389)p70s6k1
p70s6k1
SLC38A8
SLC38A8
β-actin
β-actin

Fig 10C

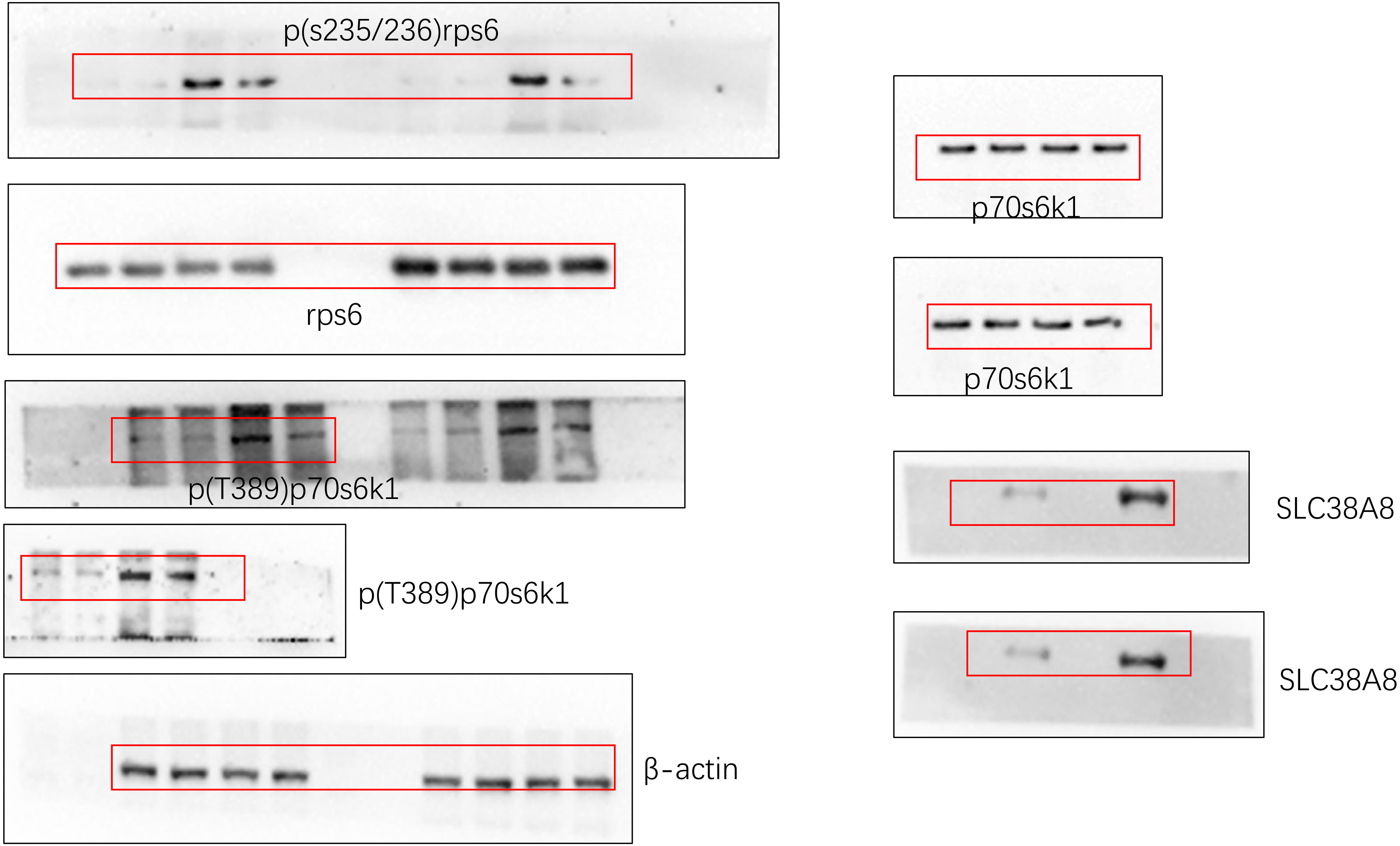
p(s235/236)rps6
rps6
p(T389)p70s6k1
p(T389)p70s6k1
β-actin
p70s6k1
p70s6k1
SLC38A8
SLC38A8

Fig 10D

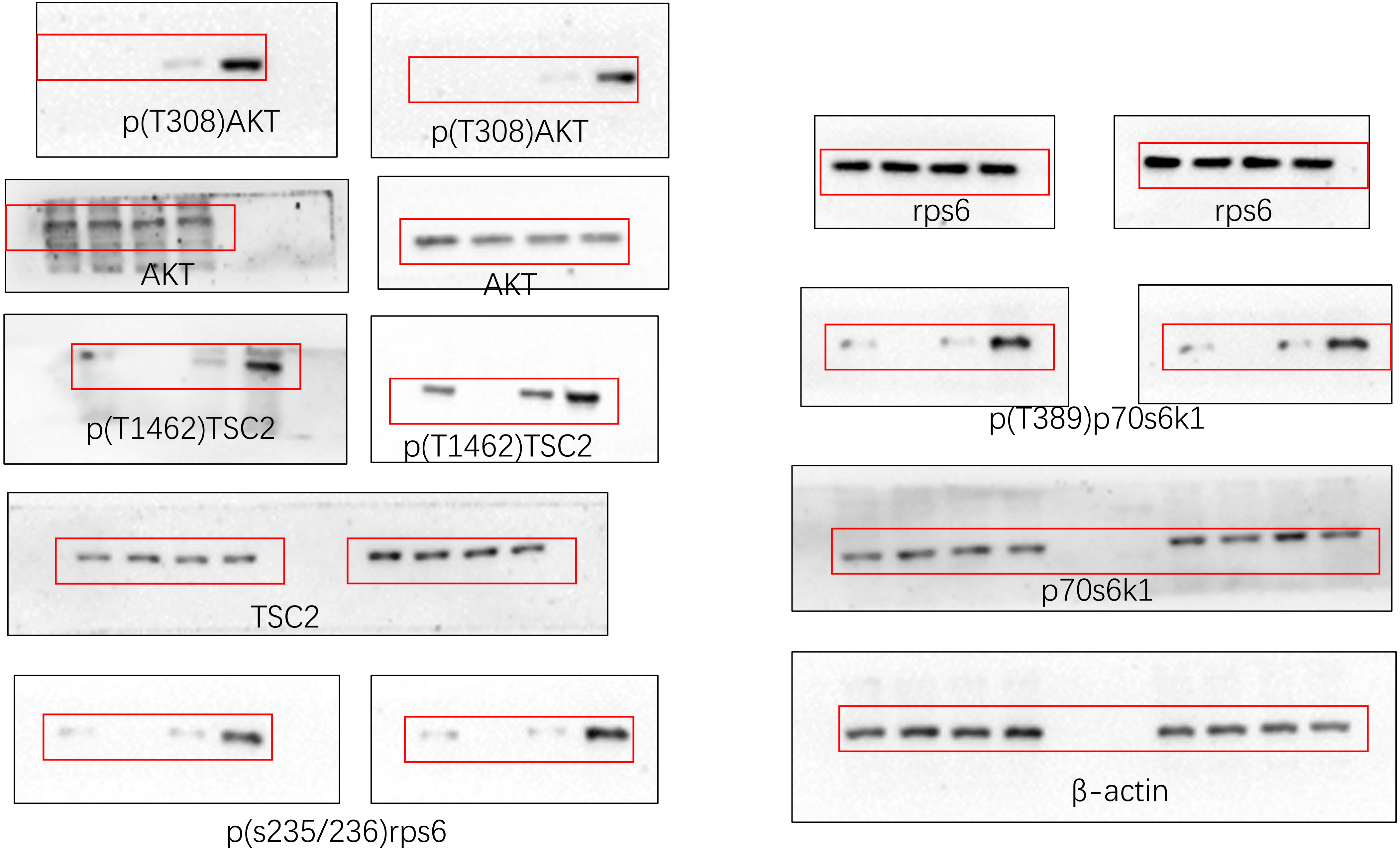
p(T308)AKT
p(T308)AKT
AKT
AKT
p(T1462)TSC2
p(T1462)TSC2
TSC2
p(s235/236)rps6
rps6
rps6
p(T389)p70s6k1
p70s6k1
β-actin

Fig 10E

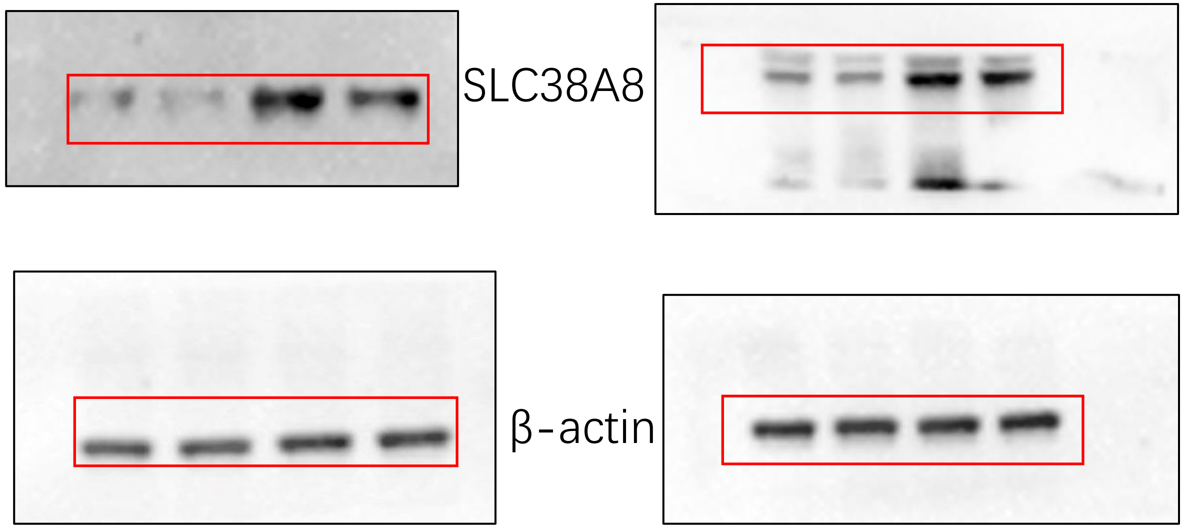

Fig 10G

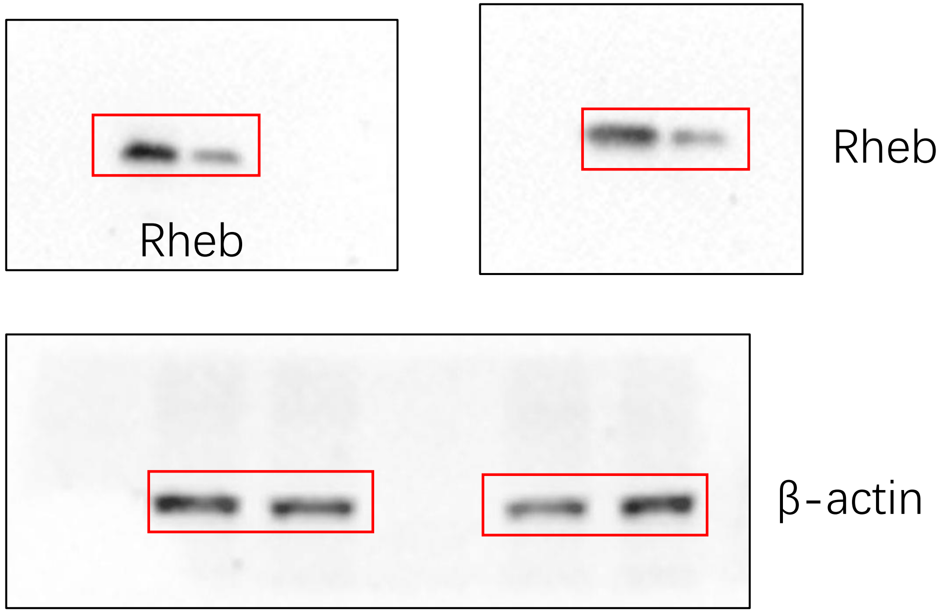

Supplement: S4 Data — (PDF) [file ppat.1011126.s015.pdf]
